# Supplementary material for: Can natural language processing models extract and classify instances of interpersonal violence in mental healthcare electronic records: an applied evaluative study
Source: BMJ Open. 2022 Feb 16;12(2):e052911. doi: 10.1136/bmjopen-2021-052911 (PMC8852656; doi:10.1136/bmjopen-2021-052911)
Supplement: Supplementary data [file bmjopen-2021-052911supp001.pdf]

Appendix 1

Keywords

Nouns

% abus%
% attack%
% beat%
% violenc%
% hit%
% rape%
% assault%

Verbs:

% fight%
% fought%
% slap%
% chok%
% push%
% punch%
% strangul%
% strangl%
% threw%
% struck%
