# Supplementary material for: Can natural language processing models extract and classify instances of interpersonal violence in mental healthcare electronic records: an applied evaluative study
Source: BMJ Open. 2022 Feb 16;12(2):e052911. doi: 10.1136/bmjopen-2021-052911 (PMC8852656; doi:10.1136/bmjopen-2021-052911)
Supplement: Supplementary data [file bmjopen-2021-052911supp002.pdf]

Appendix 2: Guidelines

Guidance for violence annotations

Development team: Aurelie Mascio, Sumithra Velupillai, Riley Botelle, Marcus Williams, Rob Stewart, Vishal Bhavsar, Giouliana Kadra

Annotations were made for:

a) *violence* status: **affirmed** or negated or irrelevant. When annotated as affirmed, further annotations were made for:

b) *patient* status: perpetrator and/or victim and/or witness and/or other, and

c) violence *type*: physical and/or sexual and/or domestic (or none of the above).

NOTE: there was not a sufficient amount of examples to develop a robust classification model for *witness*, thus there is no corresponding NLP application for this category.

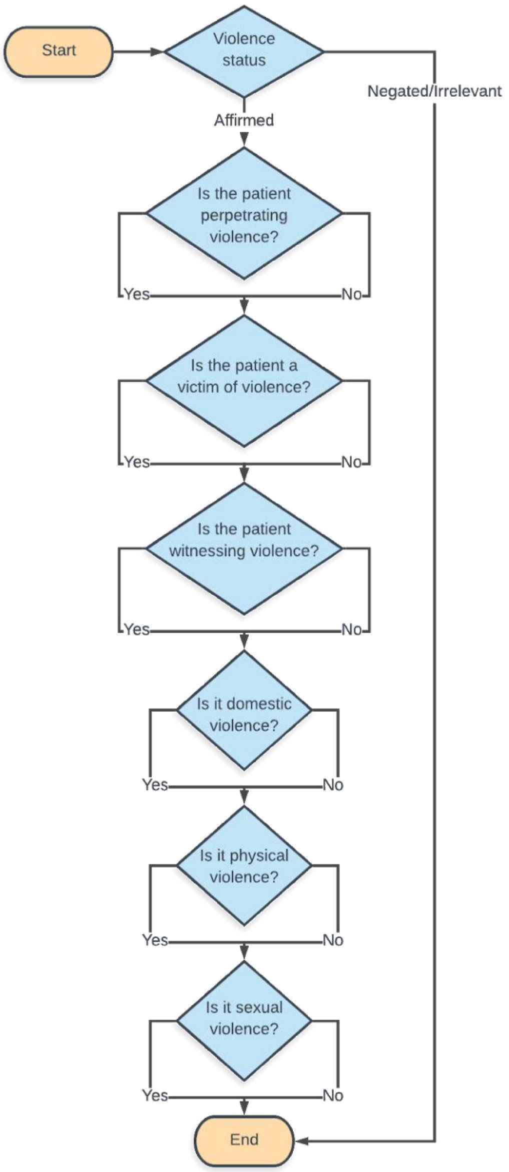

Violence status

Definition

Violence is the use of physical force or power, threatened or actual, against another person that results in - or has a high likelihood of resulting in - injury, death, psychological harm, maldevelopment, or deprivation. This does not just cover physical violence, but also violent threats, neglect, racial, digital, sexual abuse, financial abuse and coercion and control.

Our definitions of violence and sexual violence largely align with the WHO definition of violence (see also: WHO 2002 report), but with the criteria for intentionality removed due to an inability to determine this in most extracts. The violence definitions are specifically interpersonal, rather than including violence to self.

Annotation examples and criteria:

**Violence Status Decisions**		
**Examples**	**Classification**	**Explanation**
"They were *abused* in their childhood"	Affirmed	
"His brother would *beat* him up if he found out", "risk of *violence*"	Irrelevant	**Hypothetical scenarios** are irrelevant
"Expressed a lot of interest in *violence*, nazism"	Irrelevant	**Fantasies and ideological violence** is irrelevant
"Patient is *hitting* himself"	Irrelevant	**Self injury** is irrelevant
"Patient is *abusive* towards their pet"	Irrelevant	**Violence towards animals** is irrelevant
"Patient shows *violent* behaviour such as breaking objects and punching walls"	Irrelevant	**Violence directed towards objects**/property is irrelevant, affirmed only if clear intention to cause harm or fear to others.
"I have enclosed a leaflet for sexual abuse services" "referred to the domestic *violence* advisor"	Irrelevant	**Referrals** are irrelevant.
Learning difficulties/REM sleep behaviour disorder/violence without intent to harm	Affirmed	Violence is affirmed regardless of **intent**.
"Acquitted as accomplice to gang *rape* (negated) . . . convicted of ABH (affirmed)"	See explanation	When there are **multiple incidents** within the text use the keyword to determine if negated or affirmed.
"Denied the allegations he *hit* members of staff" "Questioned by police recently for allegedly *hit*ting his ex-girlfriend however he denied he was present." "Allegation made by patient of repeated sexual *assault* . . . on detailed questioning the patient denied being repeatedly assaulted"	See explanation	Denying incident = negated<br>Allegation = affirmed<br>Allegation and denial (**contrasting claims**) = affirmed<br>Not guilty = negated<br>Charges dropped = affirmed<br>Acquitted = negated<br>If confusing or unsure mark as irrelevant
"He told his mother he plans to hit them with a baseball bat"<br>"He made a gesture to attack staff"	Affirmed	**Plans** for violence are affirmed, similarly gestures or 'appearances' of violence are affirmed
"Patient was *abus*ing staff verbally"<br>"patient made threats to kill and *attack* staff"	Affirmed	**Verbal**/**threats** of violence are affirmed as violence status

		(but are not affirmed as physical violence, see below?)
"No *violence* or aggression noted"	Negated	
"Found the way they were brought to the hospital very traumatic and therefore felt very *abus*ed by mental health services"	Irrelevant	**Institutional and structural violence** is irrelevant. Interpersonal violence by individuals within institutions (e.g. beaten by police, abused by prison guards) would still be affirmed
"she repeatedly called the police making allegations of being posioned on the ward . . . she also made allegations of physical and sexual *abus*e by patients and staff"<br>"the voices . . . threaten to harm her and sometimes she feels them *hit* her"	Affirmed or Irrelevant	For allegations where the patient is clearly recorded as **hallucinating or delusional** within the fragment, mark as irrelevant. If unclear if delusional or a real event, mark as affirmed.
"Forced to *fight*" (in a war/army), "was not tortured but witnessed dead bodies and forced to move frequently due to *fight*ing"	Irrelevant	**Military service** should not be captured - irrelevant<br>Collective violence should not be captured - irrelevant
"Suffered financial abuse"	Affirmed	Financial, psychological, emotional and **other types of abuse** including deprivation and neglect are affirmed.
"He has a history of *violence*"<br>"patient used to *hit* her partner"<br>"Is not as verbally *abus*ive as she used to be"	Affirmed	**Historical abuse** is affirmed.

Patient status

Definition

The **perpetrator** is the person using physical force or power, the **victim** is the person this is used against. A **witness** is someone who directly observes acts of violence through sight and/or sound.

Annotation examples and criteria:

**Patient Status Decisions**		
**Examples**	**Classification**	**Explanation**
"he stabbed someone who had *attack*ed him",	Affirmed, perpetrator	Patient status may be any combination of victim and perpetrator and witness.

"Arrested following violence and fire setting . . . having been raped 3 years ago"	and/or victim and/or witness	
"They often argued and fought", "got into a fight", "became involved in a fight", "they had fought"	See explanation	Generally for vague **"involvement" in fights** patient status should be marked as "other". If there is clear initiation of the fight they would be the perpetrator, if there is explicit harm or intended harm to a party they would be the victim or perpetrator accordingly.
"tried to rob him . . . he *fought* back and they fled", "talked about his brother bullying him as a child until he *fought* back", "he felt another youth was threatening to him and *fought* back violently"	Perpetrator and victim	Where there is **retaliatory violence or self defence** mark the patient as both perpetrator and victim
"his son got *beat* up on the way home from school"	Other	**Indirect witnesses** or where it is unclear if they saw/heard/witnessed themselves should be marked as other.
"Watched his father being killed"	Witness	Clearly **direct witnesses** should be marked as witness.

Violence type

Definition

Domestic violence is violence between family members, intimate partners, ex-intimate partners and household members.

Physical violence is violence that uses physical force, or results in or has a high likelihood of resulting in physical injury to the victim.

Sexual violence includes any unwanted sexual act, unwanted sexual comments or advances, unwanted attempts to obtain a sexual act, directed acts against a person's sexuality using coercion and acts to traffic. It includes (but is not limited to) rape, sexual harrassment, sexual assault, forced marriage, FGM, and reproductive coercion and control.

Domestic violence is based on the UK government domestic abuse statutory guidance framework 2020, but adjusted to be applicable to all ages (rather than being restricted to over 16s) and includes people who are household members through choice (but not those in institutional, residential, detention or in-patient settings). Thresholds for considering violence physical are partially adapted from UK law, for instance "offences against the person" (1861 Act) describes crimes which are committed by direct physical harm or force and includes spitting as a form of assault and poisoning as a non-fatal, non-sexual offence.

Annotation examples and criteria:

**Violence Type Decisions**		
**Examples**	**Classification**	**Explanation**
**Violence Type Decisions (Physical)**		
“physical discipline” which was not perceived though as physical *abuse.*” “hit or made to kneel by father . . . did not see this as *abus*ive”	See explanation	Varies on keyword - these would be negated based on the negation of “abusive” keyword, but affirmed if the keyword was “physical discipline” or “hit”.
“false imprisonment”/”abduction”	Physical = yes	
“spitting”	Physical = yes	Legally considered physical assault
“spiked” (drink) or poisoning	Physical = yes	Legally considered assault by poisoning
“Tried to *hit*”, “Had to be stopped from *assaulting*”	Physical = yes	**Attempts to inflict physical violence** should be marked as physical
“*Rape*”, “sexual *assault*”	Physical = yes, sexual = yes	**Physical sexual violence** should be marked as both physical and sexual
**Violence Type Decisions (Domestic)**		
“Partner’s son”, “brother’s girlfriend”, “father”	Domestic = yes	**Familial relationships** (biological or adoptive) are considered domestic
“patient stabbed his roommate”	Domestic = yes	Those living together by choice (**households**) are domestic e.g. flatmates and cohabitees
“*abuse* she suffered whilst in care”	Domestic = no	**Residential and institutional settings** are not domestic including inpatient wards, prisons and care homes
“Hitting her ex-girlfriend”	Domestic = yes	**Partners and ex-partners** are considered domestic
**Violence Type Decisions (Sexual)**		

Sexual harassment	Sexual = yes, physical = no	**Sexual harrassment** is considered to be sexual violence.
"talked to female staff about his sexual fantasies", "indecent exposure"	Sexual = yes	Mark as sexual violence if interpersonal (e.g. directed at or caused harm to another person)
"Patient was sexually disinhibited", "Patient showed sexually inappropriate behaviour"	Irrelevant	Mark as irrelevant if not person-directed or no clear harm to another person.
