# Supplementary material for: Can natural language processing models extract and classify instances of interpersonal violence in mental healthcare electronic records: an applied evaluative study
Source: BMJ Open. 2022 Feb 16;12(2):e052911. doi: 10.1136/bmjopen-2021-052911 (PMC8852656; doi:10.1136/bmjopen-2021-052911)
Supplement: Supplementary data [file bmjopen-2021-052911supp003.pdf]

Appendix 3: Guideline development discussion

Definitions of violence

An affirmed instance of violence - what should this mean?

Our NLP definition: Violence is the use of physical force or power, threatened or actual, against another person that results in - or has a high likelihood of resulting in - harm.

Harm in this instance does not refer only to physical injury, but also covers abuses of power such as violent threats and intimidation, acts which result in emotional and psychological harm, neglect, sexual abuse, financial abuse and coercion and control. It does not include violence against the self.

This definition was developed from the WHO definition of violence (Krug, 2002), but adjusted using deductive reasoning based on the annotated extracts and guideline development discussions, and therefore differs in two key ways. The first is that it only identifies interpersonal violence, and the second is that it does not require the violence to be intentional in order to be affirmed. These two changes are because there are various limitations to how violence can be affirmed in a way that can be applied reliably to EHR data.

It must be interpersonal because violence that is perpetrated systemically, state violence and structural violence is harder to identify, and tends to be poorly recorded. For example the quotation that a patient "found the way they were brought to the hospital very traumatic and therefore felt very *abus*ed by mental health services" is marked as irrelevant in this approach, as there is no identifiable interpersonal violence, however "*beat*en by prison guards" would be affirmed. Violence against whole communities is also poorly captured without an interpersonal element. We chose not to include self-directed violence.

This definition excludes acts of violence that are hypothetical ("risk of *violence*") and those that are fantasies or ideological ("expressed a lot of interest in *violence*, nazism"). It includes plans for violence ("he told his mother he plans to *hit* them with a baseball bat") and attempts made at violence which are thwarted ("had to be stopped from *assaulting*"). Whilst traditional definitions of violence place importance on the concept of intentionality, in EHRs generally the data recorded is of incidents and histories, not motivations. Intentions may be repressed, denied, disagreed upon or not recorded at all. Therefore whilst intentionality is considered part of violence and hence plans and attempts are affirmed, it was considered not necessary for intentionality to be clear in order for the instance of violence to be affirmed in the NLP approach.

Annotation was additionally complicated by the mental status of patients and resultantly the reliability of the annotators (or even the original record authors) to determine whether a patient is hallucinating or delusional when reporting a violent event. A violent event cannot be assumed to be false because of its perceived improbability, and even when some aspects are impossible other aspects may be part of a real experience. Guidelines on this must choose whether to err on the side of affirming violence that did not happen against the risk of misassigning true experiences of patient-reported violence as irrelevant. The problem with the latter is that it may create a situation where patients with delusional or hallucinatory experiences are less likely to have instances of violence identified, and we considered that the resultant harms from this outweighed the overestimation of violence by including some false events. For instance, the example "she repeatedly called the police making allegations

of being posioned on the ward . . . she also made allegations of physical and sexual *abus*e by patients and staff" was not be assumed to be a delusional experience, as this risked masking real violent events. Contrastingly however "the voices . . . threaten to harm her and sometimes she feels them *hit* her" is explicitly a hallucinatory experience, and therefore not interpersonal and not affirmed.

Other difficulties with identifying violence reflect the entanglement of multiple viewpoints within the text. How should we classify a violent act that the author is asserting happened but the patient denies? Or similarly, an allegation by one party and a denial by another in the same text, or even an allegation and later denial by the same patient? (For instance, "allegation made by patient of repeated sexual *assault* . . . on detailed questioning the patient denied being repeatedly assaulted.") How should legal terms be interpreted - someone who is charged, or acquitted, or who's case against them is dropped? Our approach tended towards affirming the identification of violent acts where there was a degree of uncertainty or disagreement as to whether they occurred or not, and only excluding those where everyone was in agreement there had been no violence, for the same concerns about harms resulting from the classification of reported violence as false. This overall approach should lend itself to an output where the application is more likely to produce false positives than false negatives.

Thresholds for considering something as violence could also be challenging. Our definition includes actions or inaction that results in harm, but what may cause harm for one person may not cause harm to others. As a general rule the guidelines do not include violent acts against property or objects as these are not interpersonal, but destroying a valued object to punish someone or throwing something in order to incite fear are both interpersonal violent acts that should be identified and affirmed. Violence towards animals was similarly excluded from the definition for not being interpersonal. There was occasionally difficulty in differentiating between aggressive behaviour and violence. The majority of aggressive behaviour, if directed at another person, fulfils the definition of violence and was appropriately accounted for by these guidelines.

Defining patient status - who did what?

Our NLP definition: The **perpetrator** is the person using physical force or power, the **victim** is the person this is used against. A **witness** is someone who directly observes acts of violence through sight and/or sound.

Identifying the role of the patient (presupposing you can identify who the patient is in the text) was on average a straightforward process, and usually explicitly stated. There are two exceptions we found to this; instances where there was some level of mutuality in the violence, and instances where the violence was retaliatory or undertaken as self-defence. To resolve these problems, patients could be assigned both as the perpetrator and the victim in scenarios where they both enacted and received violence. It has been argued that self-defence should not be considered violence (Hamby, 2017) but extracts from EHRs often included disagreement as to whether an event was truly self-defence or not, and thus following the rules for disagreements between people or the authors of the texts, were marked as affirmed.

Types of violence

Our NLP definition of physical violence is violence that uses physical force, or results in or has a high likelihood of resulting in, physical injury to the victim.

Where there was uncertainty about whether an act should be considered physical violence, such as spitting and poisoning, an exemplar approach was utilised. These utilised categorisations adapted from UK law such as "offences against the person" (1861 Act) which describes crimes which are committed by direct physical harm or force and includes spitting as a form of assault and poisoning as a form of non-fatal, non-sexual offence. False imprisonment and abduction were agreed by discussion to be a form of physical violence due to the physically restricting nature of the act.

One difficulty with establishing whether physical violence should be affirmed is with acts of physical violence that were not considered to result in harm by the victim, such as "hit or made to kneel by father . . . did not see this as *abus*ive". There are two potential approaches here; either choosing which to prioritise between actions and harms, or using the specific keywords to make two separate judgements. With the latter approach, the example would appear twice, once as affirmed for "hit" and once as negated for "abusive".

Our NLP definition of domestic violence is violence between family members, intimate partners, ex-intimate partners and household members.

Our definition of domestic violence is designed to capture a relational aspect between the victim and perpetrator; that is it represents a family, intimate partner and/or domestic (household) relationship.

Some legal definitions of domestic violence (e.g. South African Domestic Violence Act 1998) include housemates and flatmates who share or have recently shared the same residence, whereas others only refer to previous or current intimate partners and family members. Our definition, whilst including household members, only includes those who live together through choice. Therefore it includes housemates and cohabitees but not those in care homes, prisons, residential or inpatient psychiatric settings.

The UK government definition does not include those under the age of 16 within their cross-governmental definition of domestic violence, but we chose to include children and adolescents in our approach, in part because it is not always possible to determine the age of the patient from the extract but also because we felt it was not appropriate to exclude them.

The rationale for identifying domestic violence specifically is that the interventions are distinct from those designed for community violence (violence perpetrated by strangers or acquaintances) (Krug, 2002).

Our NLP definition of sexual violence includes any unwanted sexual act, unwanted sexual comments or advances, unwanted attempts to obtain a sexual act and acts to traffic. It includes rape, sexual harrassment, sexual assault, forced marriage, stalking and reproductive coercion and control.

Forms of sexual violence that fulfil the definition for physical violence, such as rape and sexual assault, were recorded as both physical and sexual violence.

A challenge with defining sexual violence in psychiatric EHRs are the symptoms of sexual disinhibition and sexually inappropriate behaviour. Whilst the approach does not consider intentionality necessary for the affirmation of a violent act, these acts and behaviours were

not considered sexual violence unless they were directed towards another person (as opposed to generalised) or they explicitly caused harm to another person
