# Supplementary material for: Can natural language processing models extract and classify instances of interpersonal violence in mental healthcare electronic records: an applied evaluative study
Source: BMJ Open. 2022 Feb 16;12(2):e052911. doi: 10.1136/bmjopen-2021-052911 (PMC8852656; doi:10.1136/bmjopen-2021-052911)
Supplement: Author's manuscript [file bmjopen-2021-052911.draft_revisions.pdf]

BMJ Open is committed to open peer review. As part of this commitment we make the peer review history of every article we publish publicly available.

When an article is published we post the peer reviewers' comments and the authors' responses online. We also post the versions of the paper that were used during peer review. These are the versions that the peer review comments apply to.

The versions of the paper that follow are the versions that were submitted during the peer review process. They are not the versions of record or the final published versions. They should not be cited or distributed as the published version of this manuscript.

BMJ Open is an open access journal and the full, final, typeset and author-corrected version of record of the manuscript is available on our site with no access controls, subscription charges or pay-per-view fees (http://bmjopen.bmj.com).

If you have any questions on BMJ Open's open peer review process please email info.bmjopen@bmj.com

BMJ Open

Extracting and classifying violence in mental healthcare electronic records using natural language processing

Journal:	*BMJ Open*
Manuscript ID	bmjopen-2021-052911
Article Type:	Original research
Date Submitted by the Author:	05-May-2021
Complete List of Authors:	Botelle, Riley; Guy's King's and Saint Thomas' School of Medicine<br>Bhavsar, Vishal; King's College London, Section of Women's Mental Health, Department of Health Services and Population Research<br>Mascio, Aurelie; King's College London, Psychological Medicine, Institute of Psychiatry, Psychology and Neuroscience<br>Kadra-Scalzo, Giouliana; King's College London, Psychological Medicine, Institute of Psychiatry, Psychology and Neuroscience<br>Williams, Marcus; Guy's King's and Saint Thomas' School of Medicine<br>Roberts, Angus; King's College London, Biostatistics and Health Informatics; Health Data Research UK<br>Velupillai, Sumithra; King's College London, Psychological Medicine, Institute of Psychiatry, Psychology and Neuroscience<br>Stewart, Robert; King's College London, Psychological Medicine, Institute of Psychiatry, Psychology and Neuroscience; South London and Maudsley Mental Health NHS Trust
Keywords:	Health informatics < BIOTECHNOLOGY & BIOINFORMATICS, MENTAL HEALTH, PSYCHIATRY, PUBLIC HEALTH

SCHOLARONE™
Manuscripts

I, the Submitting Author has the right to grant and does grant on behalf of all authors of the Work (as defined in the below author licence), an exclusive licence and/or a non-exclusive licence for contributions from authors who are: i) UK Crown employees; ii) where BMJ has agreed a CC-BY licence shall apply, and/or iii) in accordance with the terms applicable for US Federal Government officers or employees acting as part of their official duties; on a worldwide, perpetual, irrevocable, royalty-free basis to BMJ Publishing Group Ltd ("BMJ") its licensees and where the relevant Journal is co-owned by BMJ to the co-owners of the Journal, to publish the Work in this journal and any other BMJ products and to exploit all rights, as set out in our licence.

The Submitting Author accepts and understands that any supply made under these terms is made by BMJ to the Submitting Author unless you are acting as an employee on behalf of your employer or a postgraduate student of an affiliated institution which is paying any applicable article publishing charge ("APC") for Open Access articles. Where the Submitting Author wishes to make the Work available on an Open Access basis (and intends to pay the relevant APC), the terms of reuse of such Open Access shall be governed by a Creative Commons licence – details of these licences and which Creative Commons licence will apply to this Work are set out in our licence referred to above.

Other than as permitted in any relevant BMJ Author's Self Archiving Policies, I confirm this Work has not been accepted for publication elsewhere, is not being considered for publication elsewhere and does not duplicate material already published. I confirm all authors consent to publication of this Work and authorise the granting of this licence.

For peer review only

Extracting and classifying violence in mental healthcare electronic records using natural language processing

Riley Botelle (1), Vishal Bhavsar (2), Giouliana Kadra-Scalzo (3), Aurelie Mascio (3), Marcus Williams (1), Angus Roberts (4,5), Sumithra Velupillai (3) and Robert Stewart (3,6)

1. GKT School of Medical Education, King's College London
2. Section of Women's Mental Health, Department of Health Services and Population Research, King's College London.
3. Psychological Medicine, Institute of Psychiatry, Psychology and Neuroscience, King's College London
4. Biostatistics and Health Informatics, King's College London
5. Health Data Research UK
6. South London and Maudsley NHS Foundation Trust, London

Corresponding author's details: riley.botelle@gmail.com

Funding: This project received no specific funding. The Clinical Record Interactive Search (CRIS) system was funded and developed by the National Institute for Health Research (NIHR) Biomedical Research Centre at South London and Maudsley NHS Foundation Trust and King's College London and by a joint infrastructure grant from Guy's and St Thomas' Charity and the Maudsley Charity (grant number BRC-2011-10035). RS, SV, AR and GKS receive salary support from the National Institute for Health Research (NIHR) Biomedical Research Centre at South London and Maudsley NHS Foundation Trust and King's College London. RS is a NIHR Senior Investigator. GKS has received support from the Early Career Research Award, funded by the NIHR Maudsley BRC. VB receives salary support from King's College London via a secondment to Lambeth Council. AR receives support from Health Data Research UK, an initiative funded by UK Research and Innovation, Department of Health and Social Care (England) and the devolved administrations, and leading medical research charities. The views expressed are those of the author(s) and not necessarily those of the NHS, the NIHR or the Department of Health. Funders had no role in the study design; in the collection, analysis and interpretation of the data; in the writing of the report; and in the decision to submit the paper for publication.

Competing Interests: RS has received research support in the last 36 months from Janssen, Takeda and GSK. GKS has received funding from Janssen and Lundbeck. SV has received funding from Janssen. AM has received funding from Takeda California, Inc.

Data Availability: The CRIS database has received ethical approval for secondary analysis: Oxford REC C, reference 18/SC/0372. On request, and after appropriate arrangements, the data and modelling employed in this study can be viewed within the secure system firewall.

Word count: 3437 words, 2 figures, 5 tables

For peer review only

Abstract

Objective: We developed and evaluated natural language processing (NLP) models for extracting clinical text referring to violence using electronic health records (EHRs) from a large mental healthcare provider.

Design: A multidisciplinary team iteratively developed guidelines for annotating clinical text referring to violence. Keywords were used to generate a dataset which was annotated (i.e. classified as affirmed, negated or irrelevant) for: presence of violence, patient status (i.e. as perpetrator, witness and/or victim of violence) and violence type (domestic, physical and/or sexual). An NLP approach using a pre-trained transformer model (BioBERT) was fine-tuned on the annotated dataset and evaluated using 10-fold cross-validation.

Setting: We used the Clinical Records Interactive Search (CRIS) database, comprising over 500,000 de-identified EHRs of patients receiving input from the South London and Maudsley NHS Foundation Trust, a specialist mental healthcare provider serving an urban geographic catchment area.

Participants: Searches of CRIS were carried out based on 17 pre-defined keywords. Randomly selected text fragments were taken from the results for each keyword, amounting to 3771 text fragments extracted from the records of 2832 patients.

Outcome measures: We estimated precision, recall and F1 score for each NLP model. We examined sociodemographic and clinical variables in patients giving rise to the text data, and frequencies for each annotated violence characteristic in the data.

Results: Binary classification models were developed for six labels (violence presence, perpetrator, victim, domestic, physical and sexual). Among annotations affirmed for the presence of any violence, 78% (1724) referred to physical violence, 61% (1350) referred to patients as perpetrator, and 33% (731) to domestic violence. NLP models' precision ranged from 89% (perpetrator) to 98% (sexual); recall ranged from 89% (victim, perpetrator) to 97% (sexual).

Conclusions: State of the art NLP models can extract and classify clinical text on violence from EHRs at acceptable levels of scale, efficiency and accuracy.

Key terms: natural language processing, NLP, violence, victimisation, perpetrator, electronic health records

Strengths and limitations of this study:

- Previous NLP models for extracting violence from mental health EHRs have focused on single forms of violence, rather than capturing violence more broadly.
- Six NLP models for extracting references to interpersonal violence information in mental health clinical text, including patient status (perpetrator, victim) and violence type (domestic, physical and sexual), were successfully developed.
- This article fills a gap where newer fine-tuned transformer-based NLP models such as Bio-BERT have not yet been extensively researched in mental health applications.
- For one planned NLP model, witness to violence, only small numbers of text extracts were available based on the keyword sampling approach, and this model could not be developed.
- NLP algorithms can estimate the occurrence of clinical references to violence in EHRs - they should not be used to estimate the prevalence of violence events without further assumptions.

For peer review only

For peer review only

Introduction

Interpersonal violence, defined as the intentional use of physical force or power, threatened or actual, against another person [1], causes significant mental and physical morbidity [2–4]. Interpersonal violence may further be distinguished as domestic, physical and sexual violence. By its definition, interpersonal violence involves one or more perpetrator(s), one or more victim(s), and may also involve witnesses.

People with mental illness are more likely to experience violent victimisation compared to the general population [5]. For example, women with pre-existing mental illness are significantly more likely to experience victimization compared to the general population with 15–45% of patients reporting experiences of victimization in the past year, and 40–90% reporting lifetime victimization [5,6]. Domestic violence victimisation is also more frequently reported by people with mental illness, with 27% of women and 13% of men with severe mental illnesses (SMI) reporting experiences of domestic violence in the past year, compared to 9% and 5% respectively in general population samples [6]. Individuals with established mental disorders also experience greater occurrence of community and sexual violence compared to the general population [7]. Evidence also suggests associations between a diagnosis of SMI and higher perpetration of violence, compared to the general population [8]. Literature examining witnessing of violence is sparse, nevertheless there are some evidence indicating greater rates of witnessed violence among people with mental illness, and a deterimental impact of witnessed violence on mental health [9,10]. Adults who have experienced victimization, and more specifically physical or sexual assault are at greater risk of mental disorders including PTSD, depression, and psychosis [11,12]. The impact of violence is mutlifold with significant economic, service and personal costs. For example, within mental health settings, violence occurs most frequently on inpatient psychiatric units, with an estimated cost of £20.5 million per year [13].

Because of the consistent correlation between violence and psychiatric morbidity, mental health services are important settings for understanding and improving societal responses to violence. In particular, a large proportion of individuals in contact with mental health services have a history of violence exposure, including through victimisation, perpetration, and/or witnessing interpersonal violence. Despite this, health services' data on interpersonal violence (including the role of patient as perpetrator, victim, or witness, whether violence being referred to is domestic, physical and/or sexual) are inconsistent. Not all forms of violence are enquired about routinely by professionals, and some forms of violence are not routinely assigned diagnostic codes [14] and so are not easily identifiable in electronic records.

Electronic health records kept by mental health services offer a valuable resource to understand how and why interpersonal violence occurs in this population and examine how

For peer review only

services respond to violence presentation (both as a victim and perpetrator) in relation to treatment and support. Improved understanding of interpersonal violence experienced by people using mental health services, and the response of professionals to violence, could improve care quality and patient safety. Mental health services can collect and record data on interpersonal violence, but structured data (e.g. on violent incidents) are predominantly collected on individuals in inpatient settings, and not all forms of violence experienced and reported by patients may be recorded in this way [15]. We have previously employed text-processing rules to extract violence information from unstructured clinical text from electronic health records, with a focus on physical violence [16].

Natural language processing (NLP) methods offer a flexible automated approach to extracting text data from large bodies of unstructured text [17,18]. NLP models have been developed for the extraction of information on diagnosis, symptoms, and treatment from clinical text [15–17]. Early NLP investigations using systems such as MedLEE relied on pattern matching and logical rules [20]. Developments in machine learning led to further advances and broad coverage applications, such as cTAKES [21] and CLAMP [22]. In mental health research, NLP models have been developed to extract constructs such as phenotype mentions [23], and symptoms of severe mental illness [22]. Recent advances in deep neural network algorithms for NLP modelling, and particularly in transformer-based language models such as BERT [24] have shown promising results, also in the biomedical domain with, for example, the BioBERT model [25], as shown in comparative analyses [26,27]. However, while these state-of-the-art approaches generate greater accuracy, they have not yet been extensively researched in mental health services.

Previous NLP approaches to capturing violence in mental health clinical text have had significant limitations such as focusing on single forms forms of violence i.e. physical assault victimisation [28], rather than capturing a broad range of possibly co-existing violence characteristics (e.g. perpetration, victimisation, witnessing), forms of harm (physical, sexual), and the nature of the relationship between victim and perpetrator (domestic, community) in the same NLP approach. Additionally, previous literature has utilised limited data e.g. only inpatient data. Unstructured data is challenging to examine due to complexity and volume, as evidenced by paucity of usage in previous studies.

Objective

To develop and evaluate natural language processing models for the extraction and classification of references to interpersonal violence from clinical text drawn from EHRs at a large mental health provider.

Methods

For peer review only

Data source

Data were drawn from the Clinical Record Interactive Search (CRIS), a database of de-identified EHRs from the South London and Maudsley (SLaM) NHS Foundation Trust. SLaM provides specialist mental health services to around 1.3 million residents of four boroughs in South-East London (Lambeth, Southwark, Lewisham and Croydon). The CRIS database was developed in 2008 and allows researchers to access structured data (such as demographic data from forms) and unstructured data (such as free text entered by clinicians based on clinical encounters) for mental health research [28–31]. Currently there are over 500,000 patient records represented in CRIS, spanning 2007-present. CRIS applies a robust data security and governance model for data access and has been approved as a database for secondary analysis (Oxford Research Ethics Committee, reference 18/SC/0372).

Patient and Public Involvement statement

The CRIS database was developed with extensive service user involvement and adheres to strict governance frameworks managed by service users. Data are used in an entirely de-identified and secure format and all patients have the choice to opt-out of their de-identified data being used [32]. Each research project is reviewed by a service-user led oversight committee of the National Institute of Health Research Biomedical Research Centre.

Methodological framework for annotation

Figure 1: Process of annotation, development and evaluation of NLP models

Figure 1 summarises the annotation process. To generate annotation data for NLP model development we generated a list of violence-related keywords based on the literature, clinical experience, and informatics expertise (Appendix 1). For each keyword (17 in total; 7 nouns and 10 verbs), CRIS text containing that keyword was randomly sampled, extracting fragments containing the keyword and 300 text characters either side. We aimed to select 300 text fragments for each noun keyword, and 200 for each verb keyword. These text fragments were then annotated into labels for reference to violence by two clinical medical students (RB and MW). Initial label definitions, results and queries were reviewed and discussed by a multidisciplinary team which met weekly to formulate a set of further labels for NLP development based on the violence and mental health literature. Final labels are described further below. Weekly meetings also adjudicated on disagreements between annotators. We developed annotation guidelines (Appendix 2) which were iteratively developed based on discussion and queries raised by annotation. Discussions and rationale are detailed in Appendix 3. Inter-annotator agreement was estimated on a subset, using % agreement and Cohen's kappa.

For peer review only

Labels used for annotating text fragments

Annotations were carried out for seven labels which were developed based on the WHO definition of interpersonal violence [1]. For each text label described below and exemplified in Table 1, annotations classified text fragments as follows:

1. *Violence presence*: we annotated for the presence of any reference to **violence** in the text fragment, classifying fragments into: affirmed (where the characteristic was present), negated (characteristic absent), or irrelevant (where the keyword was employed in the text to refer to a context other than violence).
2. *Patient status*: we assigned three non-exclusive labels for the status of the patient within the text fragment, classified into whether the patient was perpetrator, victim, and/or witness, to the text fragments where violence presence was annotated as affirmed. The label **perpetrator** was affirmed where the patient was referred to as the person using physical force or power, and the label **victim** was affirmed where the fragment referred to the patient as the person violence was used against. The label **witness** was affirmed where the fragment referred to the patient as having observed violence through seeing or hearing violence occurring and this was the primary description, i.e. they were not a victim or perpetrator of that violence.
3. *Violence type*: we also assigned labels classifying violence by forms of harm (physical, sexual), and based on the relationship between the victim and perpetrator (domestic). The label **physical** was affirmed where the text fragment referred to violence which used physical force, or resulted in or had a high likelihood of resulting in physical injury. The label **sexual** was affirmed where the text fragment referring to violence included unwanted sexual acts, unwanted sexual comments or advances, or unwanted attempts to obtain a sexual act. This included references to rape, sexual harrassment, sexual assault, forced marriage, stalking and reproductive coercion and control. The label **domestic** was affirmed where text referred to violence between family members, intimate partners, ex-intimate partners and household members. As with *patient status*, these labels were also non-exclusive, so that text fragments could include any combination of affirmed labels listed above.

Table 1: Examples of text fragments, with keywords italicised, extracted for annotation in this study, alongside corresponding labels and assigned annotations.

For peer review only

Example of text fragment	Label	Annotation
"They were *abused* in their childhood"	Violence presence, victim	Affirmed
"Patient used to *hit* her partner"	Violence presence, perpetrator; physical, domestic	Affirmed
"Patient stabbed his roommate"	Violence presence, perpetrator; physical, domestic	Affirmed
"Expressed a lot of interest in *violence*, nazism"	Violence presence	Irrelevant
"No *violence* or aggression noted"	Violence presence	Negated

The labels listed above were annotated in the following steps. First, each text extract was annotated for the violence presence label, classifying the fragment of text as affirmed, negated or irrelevant. If this label was affirmed, the fragment was further annotated with labels for patient status (victim, perpetrator, and/or witness) and violence type (domestic, physical, sexual). This is represented in Figure 2.

Figure 2: Flowchart of extract annotation process

NLP model development

For the development of NLP models, we used the pre-trained BioBERT model [25] and fine-tuned it on the annotated dataset. We aimed to produce seven binary classification models for each annotated label. We evaluated the models with 10-fold cross validation, comprising 10% annotated text extracts for testing, and 90% text extracts in training, in each fold. We estimated standard markers of NLP performance: precision (or positive predictive value), recall (or sensitivity) and F1 score (the harmonic mean of precision and recall), using weighted averages to take into account the dataset's imbalance (i.e. differing numbers of extracts generated for each keyword). Reported scores corresponded to the mean across the 10 test sets. A separate test was run on 1411 sentences extracted randomly from the CRIS database (sentences not used to train the model or test the development). Annotations from medical students were compared to the model results.

For descriptive purposes we examined sociodemographic and diagnostic characteristics of patients whose records gave rise to the text extracts used for NLP development, and also assessed two way overlap of affirmed non-exclusive annotation labels.

For peer review only

Results

Sampling of text for NLP development resulted in 14 of the 17 keywords being sampled as planned. Three keywords generated a smaller number of selected fragments: 'rape' 188; 'fought' 124 and 'strangul' 59, resulting in a final annotation dataset of 3771 text extracts. Fine-tuning of the pre-trained BioBERT model using the annotation dataset resulted in six binary classification models - for one annotation label (witness) we were unable to generate a model due to insufficient data size (n=53). The performance of each of the six NLP models is reported in Table 2. Two types of testing were carried out to evaluate the model's suitability. A 10 fold cross validation was conducted on the annotated dataset (comprising 3771 text extracts), for which the average performance on the test sets is reported. Precision ranged from 89% (for the perpetrator label) to 98% (sexual) and recall ranged from 89% (victim, perpetrator) to 97% (sexual). Inter-annotator agreement was high: 82-96% (60-85% Cohen's kappa) for the six annotation labels. Additionally, a separate blind test consisting of 1000 newly annotated sentences not used for model training or fine-tuning was assessed. For this blind test we used a confidence level of 90% (meaning only sentences that the model classified with 90% or above confidence were included, in order to eliminate "confusing statements", which made up 1% of the dataset). This 90% threshold was then used for application deployment.

Table 2: NLP model performances on the annotated dataset (3,771 text extracts) and well as a blind test set with a 90% probability threshold (100 sentences) for the six labels.

	**Training set (average score on 10-fold cross-validation)**			**Blind test set**
**Annotation label**	**Precision**	**Recall**	**F1-score**	**F1-score**
**Violence presence**	93%	93%	93%	95%
**Patient status: Perpetrator**	89%	89%	89%	85%
**Patient status: Victim**	91%	89%	91%	90%
**Violence type: Domestic**	94%	94%	94%	93%
**Violence type: Physical**	91%	92%	91%	98%
**Violence type: Sexual**	98%	97%	97%	93%

Table 3: Proportion of each label in the annotation dataset – affirmed or negated/irrelevant. Each text extract was first annotated for the violence presence label, then if this was affirmed, further annotated for the other labels related to patient status and violence type (see figure 1 for further details). Therefore denominator totals for the violence presence label is larger than that for the other labels.

For peer review only

Annotation label	Affirmed, N (%)	Negated or irrelevant, N (%)	Total
**Violence presence**	2199 (58)	1572 (42)	3771
**Patient status: Perpetrator**	1350 (61)	849 (39)	2199
**Patient status: Victim**	731 (33)	1468 (67)	2199
**Violence type: Domestic**	723 (33)	1476 (67)	2199
**Violence type: Physical**	1724 (78)	475 (22)	2199
**Violence type: Sexual**	353 (16)	1846 (84)	2199

Table 3 shows the breakdown of annotation labels across the annotated dataset at an annotation level. Overall, 58% (2,199) of the text extracts were affirmed for violence presence. Of these, the proportion of affirmed examples for patient status ranged from 33% (victim) to 61% (perpetrator), and for violence type from 16% (sexual) to 78% (physical). Some affirmed labels overlapped, because a fragment of text could have contained multiple references to different types of violence (e.g. both physical and sexual violence) and different patient statuses (e.g. as both perpetrator and victim). Table 4 describes these overlaps. For instance, the number of examples where *perpetrator* was affirmed (n=1350) overlapped with affirmed for *physical* violence in almost 80% of the examples (n=1078), while the overlap with *victim* was rarer (8%, n=113).

Table 4: Overlap of labels present in affirmed annotations, showing the number and percentage of annotations that shared different labels.

	Perpetrator, N (%)	Victim, N (%)	Sexual, N (%)	Physical, N (%)
**Perpetrator**	-	-	-	-
**Victim**	113 (8.4)	-	-	-
**Sexual**	150 (11.1)	199 (27.2)	-	-
**Physical**	1078 (79.9)	616 (84.3)	304 (86.1)	-
**Domestic**	331 (24.5)	318 (43.5)	104 (29.5)	593 (34.4)
Column total	*1350 (100.0)*	*731 (100.0)*	*353 (100.0)*	*1724 (100.0)*

Demographic features of the patients included in the annotated dataset sample are presented in Table 5. The sample represented in total 2,832 patients, of whom 57% were female, 45% were aged 40-60 at the time the fragment was extracted, 66% were single, 52% were of White ethnic background, and 36% were diagnosed with psychotic disorders

For peer review only

(ICD-10 codes F20-29) [33]. This NLP approach captures references to violence occurring in a large body of patients presenting to a mental health service who are not necessarily presenting for violence-related reasons.

Table 5: Characteristics of patients whose text extracts were annotated as part of this study.

	Frequency, N (%)
**Age**	
**<20**	167 (5.9)
**20<40**	791 (27.9)
**40<60**	1273 (45.0)
**60<80**	458 (16.2)
**80<**	141 (5.0)
**Missing**	2 (0.1)
**Gender**	
**Male**	1216 (42.9)
**Female**	1614 (57.0)
**Missing**	2 (0.1)
**Marital status**	
**Single**	1865 (65.9)
**Married/cohabiting**	344 (12.2)
**Divorced/separated**	262 (9.3)
**Widowed**	85 (3.0)
**Missing**	276 (9.8)
**Ethnicity**	
**White**	1482 (52.3)
**Black**	104 (3.7)
**Asian**	160 (5.7)
**Mixed**	885 (31.3)
**Other**	90 (3.2)
**Missing**	111 (3.9)
**ICD-10 Diagnosis**	
**F0-9: Organic, including symptomatic, mental disorders**	185 (6.5)
**F10-19: Mental and**	94 (3.3)

For peer review only

behavioural disorders due to psychoactive substance use	
F20-29: Schizophrenia, schizotypal and delusional disorders	1031 (36.4)
F30-39: Mood [affective] disorders	451 (15.9)
F40-49: Neurotic, stress-related and somatoform disorders	203 (7.2)
F50-59: Behavioural syndromes associated with physiological disturbances and physical factors	18 (0.6)
F60-69: Disorders of adult personality and behaviour	236 (8.3)
F70-79: Mental retardation	53 (1.9)
F80-89: Disorders of psychological development	98 (3.5)
F90-99: Behavioural and emotional disorders with onset usually occurring in childhood and adolescence and unspecified mental disorder	211 (7.6)
No axis 1 diagnosis	25 (0.9)
G: Diseases of the nervous system, X: Intentional self-harm, assault or Z: Factors influencing health status and contact with health services*	163 (5.8)
Missing	64 (2.3)
**Total**	**2832** (100.0)

*ICD-10 categories G and X were combined with ICD-10 category Z due to small numbers of participants (n<10) in these categories, in order to limit identification of participants.

For peer review only

Discussion

To our knowledge, this is the first study that has used a NLP approach to code free-text data from a large and diverse source of electronic mental health records to ascertain violence according to presence, agent and type. There is limited previous research examining NLP to extract violence-related information in EHRs but includes utilising text rules to ascertain violent behaviour as antecedents to supervised confinement [16] and the employment of a bag of words machine learning approach to extract information on physical assault victimisation in CRIS data [28]. Our approach captures a much broader range of experiences. We successfully developed an annotated dataset of clinical text references to interpersonal violence, using a multidisciplinary clinical academic group. We used this dataset to develop binary classification NLP models for extracting and classifying clinical text fragments referring to interpersonal violence in mental health EHRs, including for patient status (perpetrator, victim) and violence type (domestic, physical, sexual). Models were developed with a state-of-the-art NLP algorithm (fine-tuned BioBERT) and displayed very good performance based on accepted evaluation criteria. A planned NLP model for extracting references to witnessed violence was not successfully developed due to an insufficient sample size.

This study had several limitations that need to be borne in mind when interpreting the findings. Although agreement between annotators was generally good, some disagreements occurred. Examples of disagreements included ascertaining the status of the patient as perpetrator/victim/witness in a text fragment and whether “fighting” was considered interpersonal violence or a colloquial term for verbal arguments. As our focus was on interpersonal violence, we did not annotate or develop models for individuals forced to fight in armed conflict, for example. Keywords used in this study were selected to capture as many instances of interpersonal violence in clinical text as possible but may not have captured all violence categories of “hidden violence”. For instance, keywords related to female genital mutilation, forced marriage, trafficking, neglect, sensory deprivation, harrassment, stalking, or reproductive coercion and control were not included, and would need separate analysis for NLP development. These specific forms of violence could, however, be readily addressed using an “add-on” to the approach presented here. Similarly, emotional and psychological violence were not included in the list of keywords, and capturing these characteristics through NLP is likely to be more complex because of the broader way in which this is likely to be described in clinical text. Annotation was carried out on the basis of the meaning and sense of the text fragments sample in this study, rather than entire EHR documents. It is possible that annotations based on entire documents would

For peer review only

have delivered slightly different results, but is likely to have been unfeasible given the size of possible text data available. Lastly, we present an NLP approach to extracting clinical text fragments which refer to violence from mental health records. Given the sufficiently accurate performance reported in this study, this approach can extract references to violence where it is written down in clinical records, but is restricted to these recorded instances, and cannot be considered a method for measuring prevalence of all experienced violence (without further assumptions which are likely to depend on the situation). This is likely to continue to require asking patients themselves, or linking data from other sources such as hospital data or crime records. Indeed, these approaches could be helpful in understanding the processes by which violence is identified by clinicians and recorded in clinical notes.

This study had some specific advantages. Annotations were derived from rich and diverse free-text data from service-users' clinical notes, as opposed to structured data. The data are derived from progress notes entered by wide array of clinical groups and professionals, therefore increasing the chances of detecting violence information. Furthermore, given the longitudinal nature of our dataset, information was recorded over a sixteen-year period (2007-2019), which further increases our ability to detect experiences of violence, if recorded in the clinical notes. All annotations were coded using human annotators, with high inter-annotator agreement, which support the robustness of our approach. Lastly, we used a state-of-the-art NLP pre-trained transformer model, BioBERT, which has been shown to outperform methods more traditionally used for symptoms detection such as support vector machine [26] and this allowed us to develop fine-tuned models with very promising results.

NLP offers a reliable, automated, scalable approach to extracting summary information from EHRs. Reasonably accurate methods for extracting clinical text referring to interpersonal violence can support further research on correlates of clinically-identified violence, and how professionals and services respond to violence. Evaluation of NLP-derived violence indicators in relation to linked external data on hospital admissions, GP registers, and crimes data could allow some assessment of the reliability with which clinicians record violence when it occurs. Based on the inter-operability of NLP algorithms across different EHR formats, models developed in this study could be feasibly applied to mental health EHRs in other sites (including CRIS databases elsewhere in the UK) and other free-text containing EHRs.

There is also a significant clinical need for methods which can accurately summarise relevant historic information for clinicians to review and use in decision making at the point of care. This is particularly relevant in the arena of violence reduction and mental health, where serious incident reviews and charitable organizations have called for renewed attention to

For peer review only

the need for accurate summaries of previous violence, and improved information sharing with other agencies [34]. By summarising previous exposure to violence, clinical information systems might improve the efficiency of clinical encounters, e.g. by reducing clinical time taken up with collecting information on previous violence. Future research might also consider the clinical benefits of accurate summaries of previous interpersonal violence which might be reported to treating clinicians in real time, to aid decision making. We suggest that attention is warranted into the ethical and regulatory challenges of translating NLP methods for violence into practice.

We were unable to model witnessing violence using NLP in this study. This could form the focus of future research based on the possible impact of witnessing violence on health [35]. The infrequency of witnessed violence during the annotation process may reflect that clinicians are not frequently enquiring about these experiences, or they are not being recorded.

We did not consider temporality of violence mentions, but this is something that we hope to integrate in future to improve the usability of the models.

Conclusion

We have demonstrated that it is possible to use state-of-the-art NLP methods to extract clinical text referring to violence (including distinguishing patients as perpetrators and victims, as well as violence types such as physical, domestic, and sexual) at scale with acceptable accuracy in mental health EHRs. This could support further research into the pathways by which violence is identified in clinical practice, and the effectiveness of systems for identifying, assessing and managing interpersonal violence. However, while NLP approaches might offer a sufficiently accurate method for summarising violence-related information to aid clinical decision making, considerable ethical and regulatory questions remain.

Author contributions:

RB drafted the first version of the manuscript. RB and MW annotated the dataset. AM developed the NLP models. VB, GKS, RS provided clinical and epidemiological input in relation to the motivation for the work, annotation guideline development and definitions. SV and AR provided technical input and guidance in relation to the NLP model development. All authors drafted and/or critically revised the manuscript and approved the final version of the manuscript.

For peer review only

References

1 Krug EG, Mercy JA, Dahlberg LL, *et al.* The world report on violence and health. *The Lancet* 2002;**360**:1083–8. doi:10.1016/S0140-6736(02)11133-0

2 Reza A, Mercy JA, Krug E. Epidemiology of violent deaths in the world. *Injury Prevention* 2001;**7**:104–11. doi:10.1136/ip.7.2.104

3 Olofsson N, Lindqvist K, Shaw BA, *et al.* Long-term health consequences of violence exposure in adolescence: a 26–year prospective study. *BMC Public Health* 2012;**12**:411. doi:10.1186/1471-2458-12-411

4 López-Martínez AE, Serrano-Ibáñez ER, Ruiz-Párraga GT, *et al.* Physical Health Consequences of Interpersonal Trauma: A Systematic Review of the Role of Psychological Variables. *Trauma, Violence, & Abuse* 2018;**19**:305–22. doi:10.1177/1524838016659488

5 Maniglio R. Severe mental illness and criminal victimization: a systematic review. *Acta Psychiatrica Scandinavica* 2009;**119**:180–91. doi:https://doi.org/10.1111/j.1600-0447.2008.01300.x

6 Khalifeh H, Moran P, Borschmann R, *et al.* Domestic and sexual violence against patients with severe mental illness. *Psychol Med* 2015;**45**:875–86. doi:10.1017/S0033291714001962

7 Khalifeh H, Johnson S, Howard LM, *et al.* Violent and non-violent crime against adults with severe mental illness. *British Journal of Psychiatry* 2015;**206**:275–82. doi:10.1192/bjp.bp.114.147843

8 Fazel S, Gulati G, Linsell L, *et al.* Schizophrenia and violence: Systematic review and meta-analysis. *PLoS Medicine* 2009;**6**. doi:10.1371/journal.pmed.1000120

9 Kadra G, Dean K, Hotopf M, *et al.* Investigating Exposure to Violence and Mental Health in a Diverse Urban Community Sample: Data from the South East London Community Health (SELCoH) Survey. *PLOS ONE* 2014;**9**:e93660. doi:10.1371/journal.pone.0093660

10 Bhavsar V, Dean K, Hatch SL, *et al.* Psychiatric symptoms and risk of victimisation: a population-based study from Southeast London. *Epidemiology and Psychiatric Sciences* 2019;**28**:168–78. doi:10.1017/S2045796018000537

11 Resnick HS, Acierno R, Kilpatrick DG. Health impact of interpersonal violence. 2: Medical and mental health outcomes. *Behav Med* 1997;**23**:65–78. doi:10.1080/08964289709596730

12 Raune D, Bebbington P, Dunn G, *et al.* Event attributes and the content of psychotic experiences in first-episode psychosis. *PSYCHOL MED* 2006;**36**:221–30.

13 Flood C, Bowers L, Parkin D. Estimating the costs of conflict and containment on adult acute inpatient psychiatric wards. *Nurs Econ* 2008;**26**:325–30, 324.

14 Walby S. Improving the statistics on violence against women. *Statistical Journal of the United Nations ECE* 2005;**22**:193–216. doi:10.3233/SJU-2005-223-402

15 Robson D, Spaducci G, McNeill A, *et al.* Effect of implementation of a smoke-free policy on physical violence in a psychiatric inpatient setting: an interrupted time series analysis. *The Lancet Psychiatry* 2017;**4**:540–6. doi:10.1016/S2215-0366(17)30209-2

16 Cullen AE, Bowers L, Khondoker M, *et al.* Factors associated with use of psychiatric intensive care and seclusion in adult inpatient mental health services. *Epidemiology and Psychiatric Sciences* 2018;**27**:51–61. doi:10.1017/S2045796016000731

17 Ford E, Carroll JA, Smith HE, *et al.* Extracting information from the text of electronic medical records to improve case detection: a systematic review. *Journal of the American Medical Informatics Association* 2016;**23**:1007–15. doi:10.1093/jamia/ocv180

For peer review only

18 Kreimeyer K, Foster M, Pandey A, *et al.* Natural language processing systems for capturing and standardizing unstructured clinical information: A systematic review. *Journal of Biomedical Informatics* 2017;**73**:14–29. doi:10.1016/j.jbi.2017.07.012
19 Wang Y, Wang L, Rastegar-Mojarad M, *et al.* Clinical information extraction applications: A literature review. *Journal of Biomedical Informatics* 2018;**77**:34–49. doi:10.1016/j.jbi.2017.11.011
20 Friedman C, Alderson PO, Austin JH, *et al.* A general natural-language text processor for clinical radiology. *J Am Med Inform Assoc* 1994;**1**:161–74.
21 Savova GK, Masanz JJ, Ogren PV, *et al.* Mayo clinical Text Analysis and Knowledge Extraction System (cTAKES): architecture, component evaluation and applications. *J Am Med Inform Assoc* 2010;**17**:507–13. doi:10.1136/jamia.2009.001560
22 Soysal E, Wang J, Jiang M, *et al.* CLAMP – a toolkit for efficiently building customized clinical natural language processing pipelines. *J Am Med Inform Assoc* 2017;**25**:331–6. doi:10.1093/jamia/ocx132
23 Wu H, Hodgson K, Dyson S, *et al.* Efficient Reuse of Natural Language Processing Models for Phenotype-Mention Identification in Free-text Electronic Medical Records: A Phenotype Embedding Approach. *JMIR Med Inform* 2019;**7**:e14782. doi:10.2196/14782
24 Devlin J, Chang M-W, Lee K, *et al.* BERT: Pre-training of Deep Bidirectional Transformers for Language Understanding. In: *Proceedings of the 2019 Conference of the North American Chapter of the Association for Computational Linguistics: Human Language Technologies, Volume 1 (Long and Short Papers)*. Minneapolis, Minnesota: : Association for Computational Linguistics 2019. 4171–86. doi:10.18653/v1/N19-1423
25 Lee J, Yoon W, Kim S, *et al.* BioBERT: a pre-trained biomedical language representation model for biomedical text mining. *Bioinformatics* 2020;**36**:1234–40. doi:10.1093/bioinformatics/btz682
26 Mascio A, Kraljevic Z, Bean D, *et al.* Comparative Analysis of Text Classification Approaches in Electronic Health Records. *arXiv:200506624 [cs]* Published Online First: 8 May 2020.http://arxiv.org/abs/2005.06624 (accessed 18 Jan 2021).
27 Si Y, Wang J, Xu H, *et al.* Enhancing clinical concept extraction with contextual embeddings. *Journal of the American Medical Informatics Association : JAMIA* 2019;**26**. doi:10.1093/jamia/ocz096
28 Bhavsar V, Sanyal J, Patel R, *et al.* The association between neighbourhood characteristics and physical victimisation in men and women with mental disorders. *BJPsych open* 2020;**6**:e73. doi:10.1192/bjo.2020.52
29 Jackson RG, Patel R, Jayatilleke N, *et al.* Natural language processing to extract symptoms of severe mental illness from clinical text: the Clinical Record Interactive Search Comprehensive Data Extraction (CRIS-CODE) project. *BMJ Open* 2017;**7**:e012012. doi:10.1136/bmjopen-2016-012012
30 Viani N, Botelle R, Kerwin J, *et al.* A natural language processing approach for identifying temporal disease onset information from mental healthcare text. *Scientific Reports* 2021;**11**:757. doi:10.1038/s41598-020-80457-0
31 Perera G, Broadbent M, Callard F, *et al.* Cohort profile of the South London and Maudsley NHS Foundation Trust Biomedical Research Centre (SLaM BRC) Case Register: current status and recent enhancement of an Electronic Mental Health Record-derived data resource. *BMJ Open* 2016;**6**:e008721. doi:10.1136/bmjopen-2015-008721
32 Clinical Record Interactive Search (CRIS). https://www.slam.nhs.uk/quality-and-research/clinical-record-interactive-search-cris/ (accessed 2 Feb 2021).

For peer review only

33 World Health Organization, editor. *International statistical classification of diseases and related health problems*. 10th revision, 2nd edition. Geneva: : World Health Organization 2004.
34 Effectiveness of anonymised information sharing and use in health service, police, and local government partnership for preventing violence related injury: experimental study and time series analysis | The BMJ. https://www.bmj.com/content/342/bmj.d3313 (accessed 28 Jan 2021).
35 Clark C, Ryan L, Kawachi I, *et al.* Witnessing Community Violence in Residential Neighborhoods: A Mental Health Hazard for Urban Women. *J Urban Health* 2008;**85**:22–38. doi:10.1007/s11524-007-9229-8

For peer review only

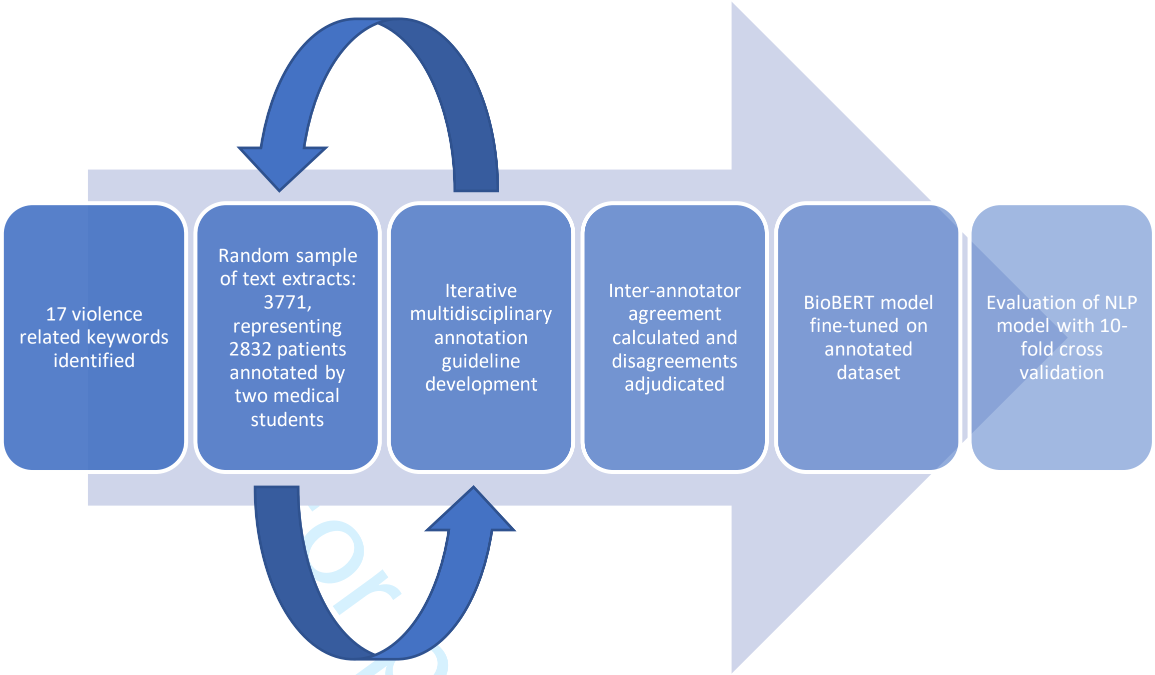

For peer review only

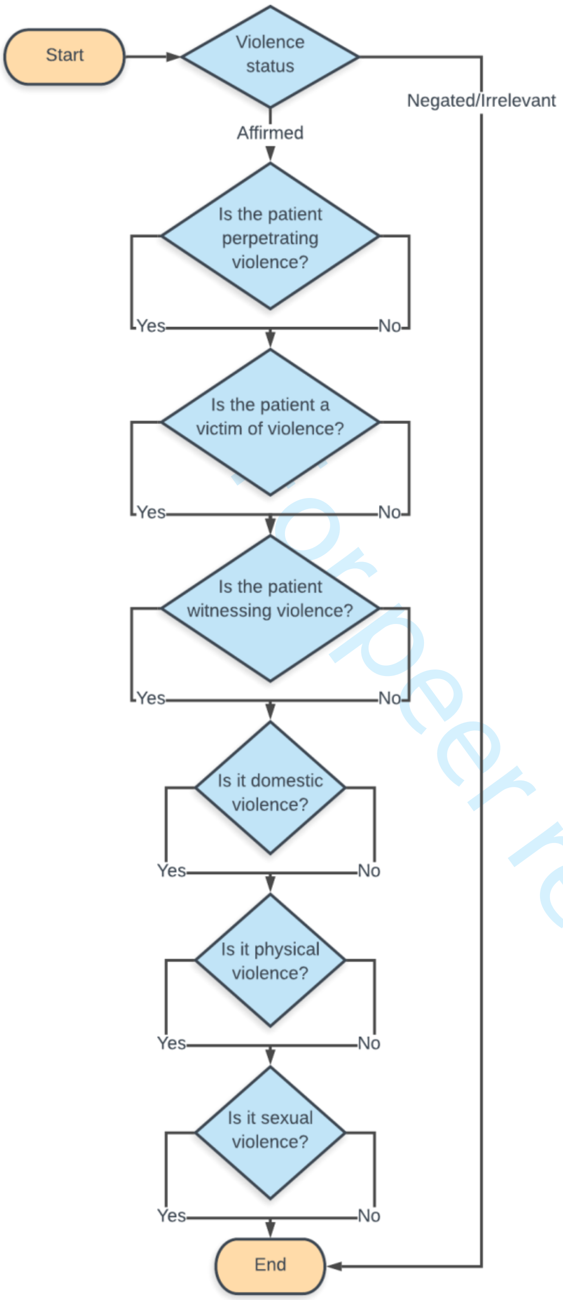

Figure 2: Flowchart of extract annotation process

Appendix 1

Keywords

Nouns

% abus%
% attack%
% beat%
% violenc%
% hit%
% rape%
% assault%

Verbs:

% fight%
% fought%
% slap%
% chok%
% push%
% punch%
% strangul%
% strangl%
% threw%
% struck%

For peer review only

Appendix 2: Guidelines

Guidance for violence annotations

Development team: Aurelie Mascio, Sumithra Velupillai, Riley Botelle, Marcus Williams, Rob Stewart, Vishal Bhavsar, Giouliana Kadra

Annotations were made for:

a) *violence* status: **affirmed** or negated or irrelevant. When annotated as affirmed, further annotations were made for:

b) *patient* status: perpetrator and/or victim and/or witness and/or other, and

c) violence *type*: physical and/or sexual and/or domestic (or none of the above).

NOTE: there was not a sufficient amount of examples to develop a robust classification model for *witness*, thus there is no corresponding NLP application for this category.

For peer review only

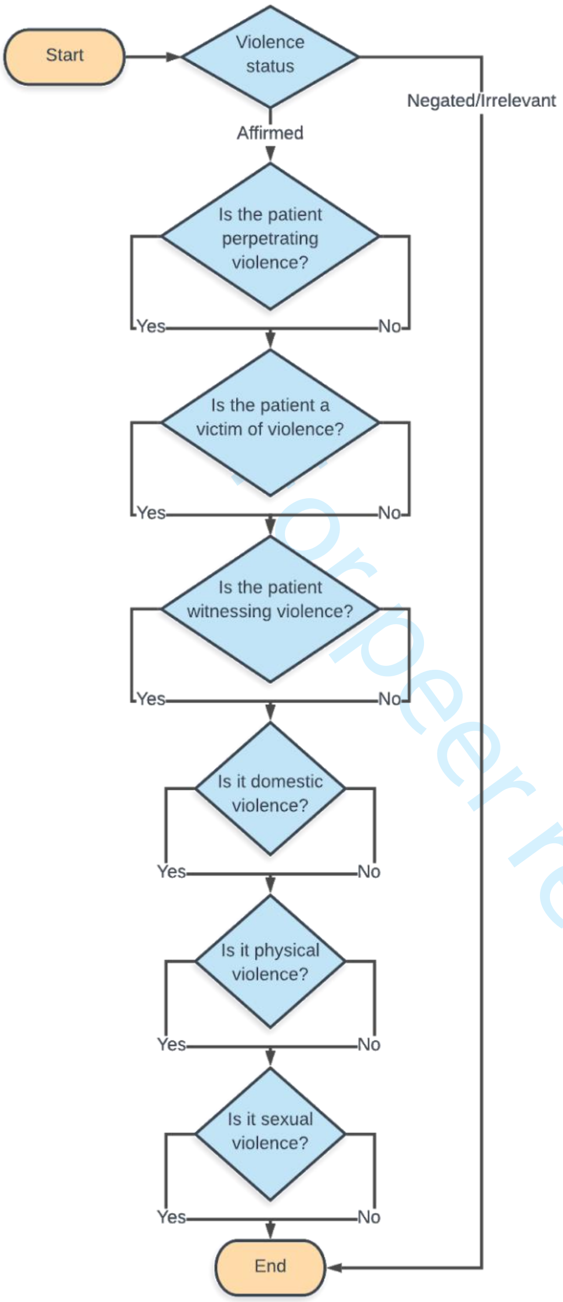

Violence status

Definition

Violence is the use of physical force or power, threatened or actual, against another person that results in - or has a high likelihood of resulting in - injury, death, psychological harm, maldevelopment, or deprivation. This does not just cover physical violence, but also violent threats, neglect, racial, digital, sexual abuse, financial abuse and coercion and control.

Our definitions of violence and sexual violence largely align with the WHO definition of violence (see also: WHO 2002 report), but with the criteria for intentionality removed due to an inability to determine this in most extracts. The violence definitions are specifically interpersonal, rather than including violence to self.

Annotation examples and criteria:

For peer review only

Violence Status Decisions		
**Examples**	**Classification**	**Explanation**
"They were *abused* in their childhood"	Affirmed	
"His brother would *beat* him up if he found out", "risk of *violence*"	Irrelevant	**Hypothetical scenarios** are irrelevant
"Expressed a lot of interest in *violence*, nazism"	Irrelevant	**Fantasies and ideological violence** is irrelevant
"Patient is *hitting* himself"	Irrelevant	**Self injury** is irrelevant
"Patient is *abusive* towards their pet"	Irrelevant	**Violence towards animals** is irrelevant
"Patient shows *violent* behaviour such as breaking objects and punching walls"	Irrelevant	**Violence directed towards objects**/property is irrelevant, affirmed only if clear intention to cause harm or fear to others.
"I have enclosed a leaflet for sexual abuse services" "referred to the domestic *violence* advisor"	Irrelevant	**Referrals** are irrelevant.
Learning difficulties/REM sleep behaviour disorder/violence without intent to harm	Affirmed	Violence is affirmed regardless of **intent**.
"Acquitted as accomplice to gang *rape* (negated) . . . convicted of ABH (affirmed)"	See explanation	When there are **multiple incidents** within the text use the keyword to determine if negated or affirmed.
"Denied the allegations he *hit* members of staff" "Questioned by police recently for allegedly *hit*ting his ex-girlfriend however he denied he was present." "Allegation made by patient of repeated sexual *assault* . . . on detailed questioning the patient denied being repeatedly assaulted"	See explanation	Denying incident = negated<br>Allegation = affirmed<br>Allegation and denial (**contrasting claims**) = affirmed<br>Not guilty = negated<br>Charges dropped = affirmed<br>Acquitted = negated<br>If confusing or unsure mark as irrelevant
"He told his mother he plans to hit them with a baseball bat"<br>"He made a gesture to attack staff"	Affirmed	**Plans** for violence are affirmed, similarly gestures or 'appearances' of violence are affirmed
"Patient was *abus*ing staff verbally" "patient made threats to kill and *attack* staff"	Affirmed	**Verbal/threats** of violence are affirmed as violence status

For peer review only

		(but are not affirmed as physical violence, see below?)
"No *violence* or aggression noted"	Negated	
"Found the way they were brought to the hospital very traumatic and therefore felt very *abus*ed by mental health services"	Irrelevant	**Institutional and structural violence** is irrelevant. Interpersonal violence by individuals within institutions (e.g. beaten by police, abused by prison guards) would still be affirmed
"she repeatedly called the police making allegations of being posioned on the ward . . . she also made allegations of physical and sexual *abuse* by patients and staff"<br>"the voices . . . threaten to harm her and sometimes she feels them *hit* her"	Affirmed or Irrelevant	For allegations where the patient is clearly recorded as **hallucinating or delusional** within the fragment, mark as irrelevant. If unclear if delusional or a real event, mark as affirmed.
"Forced to *fight*" (in a war/army), "was not tortured but witnessed dead bodies and forced to move frequently due to *fight*ing"	Irrelevant	**Military service** should not be captured - irrelevant<br>Collective violence should not be captured - irrelevant
"Suffered financial abuse"	Affirmed	Financial, psychological, emotional and **other types of abuse** including deprivation and neglect are affirmed.
"He has a history of *violence*"<br>"patient used to *hit* her partner"<br>"Is not as verbally *abus*ive as she used to be"	Affirmed	**Historical abuse** is affirmed.

Patient status

Definition

The **perpetrator** is the person using physical force or power, the **victim** is the person this is used against. A **witness** is someone who directly observes acts of violence through sight and/or sound.

Annotation examples and criteria:

**Patient Status Decisions**		
**Examples**	**Classification**	**Explanation**
"he stabbed someone who had *attack*ed him",	Affirmed, perpetrator	Patient status may be any combination of victim and perpetrator and witness.

For peer review only

"Arrested following violence and fire setting . . . having been raped 3 years ago"	and/or victim and/or witness	
"They often argued and fought", "got into a fight", "became involved in a fight", "they had fought"	See explanation	Generally for vague **"involvement" in fights** patient status should be marked as "other". If there is clear initiation of the fight they would be the perpetrator, if there is explicit harm or intended harm to a party they would be the victim or perpetrator accordingly.
"tried to rob him . . . he *fought* back and they fled", "talked about his brother bullying him as a child until he *fought* back", "he felt another youth was threatening to him and *fought* back violently"	Perpetrator and victim	Where there is **retaliatory violence or self defence** mark the patient as both perpetrator and victim
"his son got *beat* up on the way home from school"	Other	**Indirect witnesses** or where it is unclear if they saw/heard/witnessed themselves should be marked as other.
"Watched his father being killed"	Witness	Clearly **direct witnesses** should be marked as witness.

Violence type

Definition

Domestic violence is violence between family members, intimate partners, ex-intimate partners and household members.

Physical violence is violence that uses physical force, or results in or has a high likelihood of resulting in physical injury to the victim.

Sexual violence includes any unwanted sexual act, unwanted sexual comments or advances, unwanted attempts to obtain a sexual act, directed acts against a person's sexuality using coercion and acts to traffic. It includes (but is not limited to) rape, sexual harrassment, sexual assault, forced marriage, FGM, and reproductive coercion and control.

Domestic violence is based on the UK government domestic abuse statutory guidance framework 2020, but adjusted to be applicable to all ages (rather than being restricted to over 16s) and includes people who are household members through choice (but not those in institutional, residential, detention or in-patient settings). Thresholds for considering violence physical are partially adapted from UK law, for instance "offences against the person" (1861 Act) describes crimes which are committed by direct physical harm or force and includes spitting as a form of assault and poisoning as a non-fatal, non-sexual offence.

Annotation examples and criteria:

For peer review only

**Violence Type Decisions**		
**Examples**	**Classification**	**Explanation**
**Violence Type Decisions (Physical)**		
"physical discipline" which was not perceived though as physical *abuse.*" "hit or made to kneel by father . . . did not see this as *abus*ive"	See explanation	Varies on keyword - these would be negated based on the negation of "abusive" keyword, but affirmed if the keyword was "physical discipline" or "hit".
"false imprisonment"/"abduction"	Physical = yes	
"spitting"	Physical = yes	Legally considered physical assault
"spiked" (drink) or poisoning	Physical = yes	Legally considered assault by poisoning
"Tried to *hit*", "Had to be stopped from *assaulting*"	Physical = yes	**Attempts to inflict physical violence** should be marked as physical
"*Rape*", "sexual *assault*"	Physical = yes, sexual = yes	**Physical sexual violence** should be marked as both physical and sexual
**Violence Type Decisions (Domestic)**		
"Partner's son", "brother's girlfriend", "father"	Domestic = yes	**Familial relationships** (biological or adoptive) are considered domestic
"patient stabbed his roommate"	Domestic = yes	Those living together by choice (**households**) are domestic e.g. flatmates and cohabitees
"*abuse* she suffered whilst in care"	Domestic = no	**Residential and institutional settings** are not domestic including inpatient wards, prisons and care homes
"Hitting her ex-girlfriend"	Domestic = yes	**Partners and ex-partners** are considered domestic
**Violence Type Decisions (Sexual)**		

For peer review only

Sexual harassment	Sexual = yes, physical = no	**Sexual harrassment** is considered to be sexual violence.
“talked to female staff about his sexual fantasies”, “indecent exposure”	Sexual = yes	Mark as sexual violence if interpersonal (e.g. directed at or caused harm to another person)
“Patient was sexually disinhibited”, “Patient showed sexually inappropriate behaviour”	Irrelevant	Mark as irrelevant if not person-directed or no clear harm to another person.

For peer review only

Appendix 3: Guideline development discussion

Definitions of violence

An affirmed instance of violence - what should this mean?

Our NLP definition: Violence is the use of physical force or power, threatened or actual, against another person that results in - or has a high likelihood of resulting in - harm.

Harm in this instance does not refer only to physical injury, but also covers abuses of power such as violent threats and intimidation, acts which result in emotional and psychological harm, neglect, sexual abuse, financial abuse and coercion and control. It does not include violence against the self.

This definition was developed from the WHO definition of violence (Krug, 2002), but adjusted using deductive reasoning based on the annotated extracts and guideline development discussions, and therefore differs in two key ways. The first is that it only identifies interpersonal violence, and the second is that it does not require the violence to be intentional in order to be affirmed. These two changes are because there are various limitations to how violence can be affirmed in a way that can be applied reliably to EHR data.

It must be interpersonal because violence that is perpetrated systemically, state violence and structural violence is harder to identify, and tends to be poorly recorded. For example the quotation that a patient "found the way they were brought to the hospital very traumatic and therefore felt very *abus*ed by mental health services" is marked as irrelevant in this approach, as there is no identifiable interpersonal violence, however "*beat*en by prison guards" would be affirmed. Violence against whole communities is also poorly captured without an interpersonal element. We chose not to include self-directed violence.

This definition excludes acts of violence that are hypothetical ("risk of *violence*") and those that are fantasies or ideological ("expressed a lot of interest in *violence*, nazism"). It includes plans for violence ("he told his mother he plans to *hit* them with a baseball bat") and attempts made at violence which are thwarted ("had to be stopped from *assaulting*"). Whilst traditional definitions of violence place importance on the concept of intentionality, in EHRs generally the data recorded is of incidents and histories, not motivations. Intentions may be repressed, denied, disagreed upon or not recorded at all. Therefore whilst intentionality is considered part of violence and hence plans and attempts are affirmed, it was considered not necessary for intentionality to be clear in order for the instance of violence to be affirmed in the NLP approach.

Annotation was additionally complicated by the mental status of patients and resultantly the reliability of the annotators (or even the original record authors) to determine whether a patient is hallucinating or delusional when reporting a violent event. A violent event cannot be assumed to be false because of its perceived improbability, and even when some aspects are impossible other aspects may be part of a real experience. Guidelines on this must choose whether to err on the side of affirming violence that did not happen against the risk of misassigning true experiences of patient-reported violence as irrelevant. The problem with the latter is that it may create a situation where patients with delusional or hallucinatory experiences are less likely to have instances of violence identified, and we considered that the resultant harms from this outweighed the overestimation of violence by including some false events. For instance, the example "she repeatedly called the police making allegations

of being posioned on the ward . . . she also made allegations of physical and sexual *abuse* by patients and staff" was not be assumed to be a delusional experience, as this risked masking real violent events. Contrastingly however "the voices . . . threaten to harm her and sometimes she feels them *hit* her" is explicitly a hallucinatory experience, and therefore not interpersonal and not affirmed.

Other difficulties with identifying violence reflect the entanglement of multiple viewpoints within the text. How should we classify a violent act that the author is asserting happened but the patient denies? Or similarly, an allegation by one party and a denial by another in the same text, or even an allegation and later denial by the same patient? (For instance, "allegation made by patient of repeated sexual *assault* . . . on detailed questioning the patient denied being repeatedly assaulted.") How should legal terms be interpreted - someone who is charged, or acquitted, or who's case against them is dropped? Our approach tended towards affirming the identification of violent acts where there was a degree of uncertainty or disagreement as to whether they occurred or not, and only excluding those where everyone was in agreement there had been no violence, for the same concerns about harms resulting from the classification of reported violence as false. This overall approach should lend itself to an output where the application is more likely to produce false positives than false negatives.

Thresholds for considering something as violence could also be challenging. Our definition includes actions or inaction that results in harm, but what may cause harm for one person may not cause harm to others. As a general rule the guidelines do not include violent acts against property or objects as these are not interpersonal, but destroying a valued object to punish someone or throwing something in order to incite fear are both interpersonal violent acts that should be identified and affirmed. Violence towards animals was similarly excluded from the definition for not being interpersonal. There was occasionally difficulty in differentiating between aggressive behaviour and violence. The majority of aggressive behaviour, if directed at another person, fulfils the definition of violence and was appropriately accounted for by these guidelines.

Defining patient status - who did what?

Our NLP definition: The **perpetrator** is the person using physical force or power, the **victim** is the person this is used against. A **witness** is someone who directly observes acts of violence through sight and/or sound.

Identifying the role of the patient (presupposing you can identify who the patient is in the text) was on average a straightforward process, and usually explicitly stated. There are two exceptions we found to this; instances where there was some level of mutuality in the violence, and instances where the violence was retaliatory or undertaken as self-defence. To resolve these problems, patients could be assigned both as the perpetrator and the victim in scenarios where they both enacted and received violence. It has been argued that self-defence should not be considered violence (Hamby, 2017) but extracts from EHRs often included disagreement as to whether an event was truly self-defence or not, and thus following the rules for disagreements between people or the authors of the texts, were marked as affirmed.

Types of violence

For peer review only

Our NLP definition of physical violence is violence that uses physical force, or results in or has a high likelihood of resulting in, physical injury to the victim.

Where there was uncertainty about whether an act should be considered physical violence, such as spitting and poisoning, an exemplar approach was utilised. These utilised categorisations adapted from UK law such as "offences against the person" (1861 Act) which describes crimes which are committed by direct physical harm or force and includes spitting as a form of assault and poisoning as a form of non-fatal, non-sexual offence. False imprisonment and abduction were agreed by discussion to be a form of physical violence due to the physically restricting nature of the act.

One difficulty with establishing whether physical violence should be affirmed is with acts of physical violence that were not considered to result in harm by the victim, such as "hit or made to kneel by father . . . did not see this as *abus*ive". There are two potential approaches here; either choosing which to prioritise between actions and harms, or using the specific keywords to make two separate judgements. With the latter approach, the example would appear twice, once as affirmed for "hit" and once as negated for "abusive".

Our NLP definition of domestic violence is violence between family members, intimate partners, ex-intimate partners and household members.

Our definition of domestic violence is designed to capture a relational aspect between the victim and perpetrator; that is it represents a family, intimate partner and/or domestic (household) relationship.

Some legal definitions of domestic violence (e.g. South African Domestic Violence Act 1998) include housemates and flatmates who share or have recently shared the same residence, whereas others only refer to previous or current intimate partners and family members. Our definition, whilst including household members, only includes those who live together through choice. Therefore it includes housemates and cohabitees but not those in care homes, prisons, residential or inpatient psychiatric settings.

The UK government definition does not include those under the age of 16 within their cross-governmental definition of domestic violence, but we chose to include children and adolescents in our approach, in part because it is not always possible to determine the age of the patient from the extract but also because we felt it was not appropriate to exclude them.

The rationale for identifying domestic violence specifically is that the interventions are distinct from those designed for community violence (violence perpetrated by strangers or acquaintances) (Krug, 2002).

Our NLP definition of sexual violence includes any unwanted sexual act, unwanted sexual comments or advances, unwanted attempts to obtain a sexual act and acts to traffic. It includes rape, sexual harrassment, sexual assault, forced marriage, stalking and reproductive coercion and control.

Forms of sexual violence that fulfil the definition for physical violence, such as rape and sexual assault, were recorded as both physical and sexual violence.

A challenge with defining sexual violence in psychiatric EHRs are the symptoms of sexual disinhibition and sexually inappropriate behaviour. Whilst the approach does not consider intentionality necessary for the affirmation of a violent act, these acts and behaviours were

For peer review only

not considered sexual violence unless they were directed towards another person (as opposed to generalised) or they explicitly caused harm to another person

For peer review only

BMJ Open

Can natural language processing models extract and classify instances of interpersonal violence in mental healthcare electronic records: an applied evaluative study

Journal:	*BMJ Open*
Manuscript ID	bmjopen-2021-052911.R1
Article Type:	Original research
Date Submitted by the Author:	18-Oct-2021
Complete List of Authors:	Botelle, Riley; Guy's King's and Saint Thomas' School of Medicine<br>Bhavsar, Vishal; King's College London, Section of Women's Mental Health, Department of Health Services and Population Research<br>Mascio, Aurelie; King's College London, Psychological Medicine, Institute of Psychiatry, Psychology and Neuroscience<br>Kadra-Scalzo, Giouliana; King's College London, Psychological Medicine, Institute of Psychiatry, Psychology and Neuroscience<br>Williams, Marcus; Guy's King's and Saint Thomas' School of Medicine<br>Roberts, Angus; King's College London, Biostatistics and Health Informatics; Health Data Research UK<br>Velupillai, Sumithra; King's College London, Psychological Medicine, Institute of Psychiatry, Psychology and Neuroscience<br>Stewart, Robert; King's College London, Psychological Medicine, Institute of Psychiatry, Psychology and Neuroscience; South London and Maudsley Mental Health NHS Trust
**Primary Subject Heading**:	Health informatics
Secondary Subject Heading:	Mental health, Public health, Research methods
Keywords:	Health informatics < BIOTECHNOLOGY & BIOINFORMATICS, MENTAL HEALTH, PSYCHIATRY, PUBLIC HEALTH

SCHOLARONE™
Manuscripts

I, the Submitting Author has the right to grant and does grant on behalf of all authors of the Work (as defined in the below author licence), an exclusive licence and/or a non-exclusive licence for contributions from authors who are: i) UK Crown employees; ii) where BMJ has agreed a CC-BY licence shall apply, and/or iii) in accordance with the terms applicable for US Federal Government officers or employees acting as part of their official duties; on a worldwide, perpetual, irrevocable, royalty-free basis to BMJ Publishing Group Ltd ("BMJ") its licensees and where the relevant Journal is co-owned by BMJ to the co-owners of the Journal, to publish the Work in this journal and any other BMJ products and to exploit all rights, as set out in our licence.

The Submitting Author accepts and understands that any supply made under these terms is made by BMJ to the Submitting Author unless you are acting as an employee on behalf of your employer or a postgraduate student of an affiliated institution which is paying any applicable article publishing charge ("APC") for Open Access articles. Where the Submitting Author wishes to make the Work available on an Open Access basis (and intends to pay the relevant APC), the terms of reuse of such Open Access shall be governed by a Creative Commons licence – details of these licences and which Creative Commons licence will apply to this Work are set out in our licence referred to above.

Other than as permitted in any relevant BMJ Author's Self Archiving Policies, I confirm this Work has not been accepted for publication elsewhere, is not being considered for publication elsewhere and does not duplicate material already published. I confirm all authors consent to publication of this Work and authorise the granting of this licence.

For peer review only

Can natural language processing models extract and classify instances of interpersonal violence in mental healthcare electronic records: an applied evaluative study

Riley Botelle (1), Vishal Bhavsar (2), Giouliana Kadra-Scalzo (3), Aurelie Mascio (3), Marcus Williams (1), Angus Roberts (4,5), Sumithra Velupillai (3) and Robert Stewart (3,6)

1. GKT School of Medical Education, King's College London
2. Section of Women's Mental Health, Department of Health Services and Population Research, King's College London.
3. Psychological Medicine, Institute of Psychiatry, Psychology and Neuroscience, King's College London
4. Biostatistics and Health Informatics, King's College London
5. Health Data Research UK
6. South London and Maudsley NHS Foundation Trust, London

Corresponding author's details: riley.botelle@gmail.com

Funding: This project received no specific funding. The Clinical Record Interactive Search (CRIS) system was funded and developed by the National Institute for Health Research (NIHR) Biomedical Research Centre at South London and Maudsley NHS Foundation Trust and King's College London and by a joint infrastructure grant from Guy's and St Thomas' Charity and the Maudsley Charity (grant number BRC-2011-10035). RS, SV, AR and GKS receive salary support from the National Institute for Health Research (NIHR) Biomedical Research Centre at South London and Maudsley NHS Foundation Trust and King's College London. RS is a NIHR Senior Investigator. GKS has received support from the Early Career Research Award, funded by the NIHR Maudsley BRC. VB receives salary support from King's College London via a secondment to Lambeth Council. AR receives support from Health Data Research UK, an initiative funded by UK Research and Innovation, Department of Health and Social Care (England) and the devolved administrations, and leading medical research charities. The views expressed are those of the author(s) and not necessarily those of the NHS, the NIHR or the Department of Health. Funders had no role in the study design; in the collection, analysis and interpretation of the data; in the writing of the report; and in the decision to submit the paper for publication.

Competing Interests: RS has received research support in the last 36 months from Janssen, Takeda and GSK. GKS has received funding from Janssen and Lundbeck. SV has received funding from Janssen. AM has received funding from Takeda California, Inc.

Data Availability: The CRIS database has received ethical approval for secondary analysis: Oxford REC C, reference 18/SC/0372. On request, and after appropriate arrangements, the data and modelling employed in this study can be viewed within the secure system firewall.

Word count: 3769 words, 2 figures, 7 tables

For peer review only

Abstract

Objective: This paper evaluates the application of a natural language processing (NLP) model for extracting clinical text referring to interpersonal violence using electronic health records (EHRs) from a large mental healthcare provider.

Design: A multidisciplinary team iteratively developed guidelines for annotating clinical text referring to violence. Keywords were used to generate a dataset which was annotated (i.e. classified as affirmed, negated or irrelevant) for: presence of violence, patient status (i.e. as perpetrator, witness and/or victim of violence) and violence type (domestic, physical and/or sexual). An NLP approach using a pre-trained transformer model (BioBERT) was fine-tuned on the annotated dataset and evaluated using 10-fold cross-validation.

Setting: We used the Clinical Records Interactive Search (CRIS) database, comprising over 500,000 de-identified EHRs of patients within the South London and Maudsley NHS Foundation Trust, a specialist mental healthcare provider serving an urban catchment area.

Participants: Searches of CRIS were carried out based on 17 pre-defined keywords. Randomly selected text fragments were taken from the results for each keyword, amounting to 3771 text fragments from the records of 2832 patients.

Outcome measures: We estimated precision, recall and F1 score for each NLP model. We examined sociodemographic and clinical variables in patients giving rise to the text data, and frequencies for each annotated violence characteristic.

Results: Binary classification models were developed for six labels (violence presence, perpetrator, victim, domestic, physical and sexual). Among annotations affirmed for the presence of any violence, 78% (1724) referred to physical violence, 61% (1350) referred to patients as perpetrator, and 33% (731) to domestic violence. NLP models' precision ranged from 89% (perpetrator) to 98% (sexual); recall ranged from 89% (victim, perpetrator) to 97% (sexual).

Conclusions: State of the art NLP models can extract and classify clinical text on violence from EHRs at acceptable levels of scale, efficiency and accuracy.

Key terms: natural language processing, NLP, violence, victimisation, perpetrator, electronic health records

Strengths and limitations of this study:

- Previous NLP models for extracting violence from mental health EHRs have focused on single forms of violence, rather than capturing violence more broadly as this methodology does.
- This study fills a gap where newer fine-tuned transformer-based NLP models such as Bio-BERT have not yet been extensively researched in mental health applications.
- The methodology used can estimate the occurrence of clinical references to violence in EHRs but it cannot be used to estimate the prevalence of violent events without further assumptions.

For peer review only

Introduction

Interpersonal violence, defined as the intentional use of physical force or power, threatened or actual, against another person [1], causes significant mental and physical morbidity [2–4]. Interpersonal violence may further be distinguished as domestic, physical and sexual violence. By its definition, interpersonal violence involves one or more perpetrator(s), one or more victim(s), and may also involve witnesses.

People with mental illness are more likely to experience violent victimisation compared to the general population [5]. For example, women with pre-existing mental illness are significantly more likely to experience victimization compared to the general population with 15–45% of patients reporting experiences of victimization in the past year, and 40–90% reporting lifetime victimization [5,6]. Domestic violence victimisation is also more frequently reported by people with mental illness, with 27% of women and 13% of men with severe mental illnesses (SMI) reporting experiences of domestic violence in the past year, compared to 9% and 5% respectively in general population samples [6]. Individuals with established mental disorders also experience greater occurrence of community and sexual violence compared to the general population [7]. Evidence also suggests associations between a diagnosis of SMI and higher perpetration of violence, compared to the general population [8]. Literature examining witnessing of violence is sparse, nevertheless there are some evidence indicating greater rates of witnessed violence among people with mental illness, and a deterimental impact of witnessed violence on mental health [9,10]. Adults who have experienced victimization, and more specifically physical or sexual assault are at greater risk of mental disorders including PTSD, depression, and psychosis [11,12]. The impact of violence is mutlifold with significant economic, service and personal costs. For example, within mental health settings, violence occurs most frequently on inpatient psychiatric units, with an estimated cost of £20.5 million per year [13].

Because of the consistent correlation between violence and psychiatric morbidity, mental health services are important settings for understanding and improving societal responses to violence. In particular, a large proportion of individuals in contact with mental health services have a history of violence exposure, including through victimisation, perpetration, and/or witnessing interpersonal violence. Despite this, health services' data on interpersonal violence (including the role of patient as perpetrator, victim, or witness, whether violence being referred to is domestic, physical and/or sexual) are inconsistent. Not all forms of violence are enquired about routinely by professionals, and some forms of violence are not routinely assigned diagnostic codes [14] and so are not easily identifiable in electronic records.

Electronic health records kept by mental health services offer a valuable resource to understand how and why interpersonal violence occurs in this population and examine how

For peer review only

services respond to violence presentation (both as a victim and perpetrator) in relation to treatment and support. Improved understanding of interpersonal violence experienced by people using mental health services, and the response of professionals to violence, could improve care quality and patient safety. Mental health services can collect and record data on interpersonal violence, but structured data (e.g. on violent incidents) are predominantly collected on individuals in inpatient settings, and not all forms of violence experienced and reported by patients may be recorded in this way [15]. We have previously employed text-processing rules to extract violence information from unstructured clinical text from electronic health records, with a focus on physical violence [16].

Natural language processing (NLP) methods offer a flexible automated approach to extracting text data from large bodies of unstructured text [17,18]. NLP models have been developed for the extraction of information on diagnosis, symptoms, and treatment from clinical text [15–17, 19]. Early NLP investigations using systems such as MedLEE relied on pattern matching and logical rules [20]. Developments in machine learning led to further advances and broad coverage applications, such as cTAKES [21] and CLAMP [22]. In mental health research, NLP models have been developed to extract constructs such as phenotype mentions [23], and symptoms of severe mental illness [22]. Recent advances in deep neural network algorithms for NLP modelling, and particularly in transformer-based language models such as BERT [24] have shown promising results, also in the biomedical domain with, for example, the BioBERT model [25], as shown in comparative analyses [26,27]. This research indicated that using a BioBERT fine-tuned algorithm outperformed most other algorithms. However, while these state-of-the-art approaches generate greater accuracy, they have not yet been extensively researched in mental health services. This paper does not evaluate or compare models but instead builds on these previous comparative analyses to evaluate the application of an existing model (BioBERT) onto a real-world problem.

Previous NLP approaches to capturing violence in mental health clinical text have had significant limitations such as focusing on single forms forms of violence i.e. physical assault victimisation [28], rather than capturing a broad range of possibly co-existing violence characteristics (e.g. perpetration, victimisation, witnessing), forms of harm (physical, sexual), and the nature of the relationship between victim and perpetrator (domestic, community) in the same NLP approach. Additionally, previous literature has utilised limited data e.g. only inpatient data. Unstructured data is challenging to examine due to complexity and volume, as evidenced by paucity of usage in previous studies.

Objective

For peer review only

To develop and evaluate natural language processing models for the extraction and classification of references to interpersonal violence from clinical text drawn from EHRs at a large mental health provider.

Methods

Data source

Data were drawn from the Clinical Record Interactive Search (CRIS), a database of de-identified EHRs from the South London and Maudsley (SLaM) NHS Foundation Trust. SLaM provides specialist mental health services to around 1.3 million residents of four boroughs in South-East London (Lambeth, Southwark, Lewisham and Croydon). The CRIS database was developed in 2008 and allows researchers to access structured data (such as demographic data from forms) and unstructured data (such as free text entered by clinicians based on clinical encounters) for mental health research [28–31]. Currently there are over 500,000 patient records represented in CRIS, spanning 2007-present. CRIS applies a robust data security and governance model for data access and has been approved as a database for secondary analysis (Oxford Research Ethics Committee, reference 18/SC/0372).

Patient and Public Involvement statement

The CRIS database was developed with extensive service user involvement and adheres to strict governance frameworks managed by service users. Data are used in an entirely de-identified and secure format and all patients have the choice to opt-out of their de-identified data being used [32]. Each research project is reviewed by a service-user led oversight committee of the National Institute of Health Research Biomedical Research Centre.

Methodological framework for annotation

Figure 1: Process of annotation, development and evaluation of NLP models

Figure 1 summarises the annotation process. To generate annotation data for NLP model development we generated a list of violence-related keywords based on the literature, clinical experience, and informatics expertise (Appendix 1). An embedding model (Word2Vec trained on all CRIS records) was used to generate additional synonyms that were subsequently reviewed and included in the list of keywords. Furthermore, the embedding model was used to capture misspellings for each keyword.

For each keyword (17 in total; 7 nouns and 10 verbs), CRIS text containing that keyword was randomly sampled, extracting fragments containing the keyword and 300 text characters either side. We aimed to select 300 text fragments for each noun keyword, and 200 for each verb keyword. These text fragments were then annotated into labels for

For peer review only

reference to violence by two clinical medical students (RB and MW). Initial label definitions, results and queries were reviewed and discussed by a multidisciplinary team which met weekly to formulate a set of further labels for NLP development based on the violence and mental health literature. Final labels are described further below. Weekly meetings also adjudicated on disagreements between annotators. We developed annotation guidelines (Appendix 2) which were iteratively developed based on discussion and queries raised by annotation. Discussions and rationale are detailed in Appendix 3. Inter-annotator agreement was estimated on a subset, using % agreement and Cohen's kappa.

Labels used for annotating text fragments

Annotations were carried out for seven labels which were developed based on the WHO definition of interpersonal violence [1]. For each text label described below and exemplified in Table 1, annotations classified text fragments as follows:

1. *Violence presence*: we annotated for the presence of any reference to **violence** in the text fragment, classifying fragments into: affirmed (where the characteristic was present), negated (characteristic absent), or irrelevant (where the keyword was employed in the text to refer to a context other than violence).
2. *Patient status*: we assigned three non-exclusive labels for the status of the patient within the text fragment, classified into whether the patient was perpetrator, victim, and/or witness, to the text fragments where violence presence was annotated as affirmed. The label **perpetrator** was affirmed where the patient was referred to as the person using physical force or power, and the label **victim** was affirmed where the fragment referred to the patient as the person violence was used against. The label **witness** was affirmed where the fragment referred to the patient as having observed violence through seeing or hearing violence occurring and this was the primary description, i.e. they were not a victim or perpetrator of that violence.
3. *Violence type*: we also assigned labels classifying violence by forms of harm (physical, sexual), and based on the relationship between the victim and perpetrator (domestic). The label **physical** was affirmed where the text fragment referred to violence which used physical force, or resulted in or had a high likelihood of resulting in physical injury. The label **sexual** was affirmed where the text fragment referring to violence included unwanted sexual acts, unwanted sexual comments or advances, or unwanted attempts to obtain a sexual act. This included references to rape, sexual harrassment, sexual assault, forced marriage, stalking and reproductive coercion and control. The

For peer review only - http://bmjopen.bmj.com/site/about/guidelines.xhtml

label **domestic** was affirmed where text referred to violence between family members, intimate partners, ex-intimate partners and household members. As with *patient status*, these labels were also non-exclusive, so that text fragments could include any combination of affirmed labels listed above.

Table 1: Examples of text fragments, with keywords italicised, extracted for annotation in this study, alongside corresponding labels and assigned annotations.

**Example of text fragment**	**Label**	**Annotation**
"They were *abused* in their childhood"	Violence presence, victim	Affirmed
"Patient used to *hit* her partner"	Violence presence, perpetrator; physical, domestic	Affirmed
"Patient stabbed his roommate"	Violence presence, perpetrator; physical, domestic	Affirmed
"Expressed a lot of interest in *violence*, nazism"	Violence presence	Irrelevant
"No *violence* or aggression noted"	Violence presence	Negated

The labels listed above were annotated in the following steps. First, each text extract was annotated for the violence presence label, classifying the fragment of text as affirmed, negated or irrelevant. If this label was affirmed, the fragment was further annotated with labels for patient status (victim, perpetrator, and/or witness) and violence type (domestic, physical, sexual). This is represented in Figure 2.

Figure 2: Flowchart of extract annotation process

NLP model development

For the development of NLP models, we used the pre-trained BioBERT model [25] and fine-tuned it on the annotated dataset. Each set was generated independently (ensuring no overlap). Several datasets were used for: model fine-tuning, model initial testing (development stage) and model blind testing. We aimed to produce seven binary classification models for each annotated label. We evaluated the models with 10-fold cross validation, comprising 10% annotated text extracts for testing, and 90% text extracts in training, in each fold. We estimated standard markers of NLP performance: precision (or positive predictive value), recall (or sensitivity) and F1 score (the harmonic mean of precision and recall), using weighted averages to take into account the dataset's imbalance (i.e.

For peer review only

differing numbers of extracts generated for each keyword). Reported scores corresponded to the mean across the 10 test sets. A separate test was run on 1411 sentences extracted randomly from the CRIS database (sentences not used to train the model or test the development). This separate blind test set was randomly sampled using the clinical records not previously used for training, and the same list of 27 keywords. This set was then manually annotated following the same guidelines as the training set and compared against the output generated by the NLP model. Weighted loss functions (cross entropy loss with custom weight parameters) were used to account for unbalanced datasets.

For descriptive purposes we examined sociodemographic and diagnostic characteristics of patients whose records gave rise to the text extracts used for NLP development, and also assessed two way overlap of affirmed non-exclusive annotation labels.

Results

Sampling of text for NLP development resulted in 14 of the 17 keywords being sampled as planned. Three keywords generated a smaller number of selected fragments: 'rape' 188; 'fought' 124 and 'strangul' 59, resulting in a final annotation dataset of 3771 text extracts. Fine-tuning of the pre-trained BioBERT model using the annotation dataset resulted in six binary classification models - for one annotation label (witness) we were unable to generate a model due to insufficient data size (n=53). The performance of each of the six NLP models is reported in Table 2. Two types of testing were carried out to evaluate the model's suitability. A 10 fold cross validation was conducted on the annotated dataset (comprising 3771 text extracts), for which the average performance on the test sets is reported. Precision ranged from 89% (for the perpetrator label) to 98% (sexual) and recall ranged from 89% (victim, perpetrator) to 97% (sexual). Inter-annotator agreement was high: 82-96% (60-85% Cohen's kappa) for the six annotation labels. Additionally, a separate blind test consisting of 1000 newly annotated sentences not used for model training or fine-tuning was assessed. For this blind test we used a confidence level of 90% (meaning only sentences that the model classified with 90% or above confidence were included, in order to eliminate "confusing statements", which made up 1% of the dataset). This 90% threshold was then used for application deployment.

Table 2: NLP model performances on the annotated dataset (3,771 text extracts) and well as a blind test set with a 90% probability threshold (100 sentences) for the six labels.

	**Training set (average score on 10-fold cross-validation)**	**Blind test set**

For peer review only

Annotation label	Precision	Recall	F1-score	F1-score
**Violence presence**	93%	93%	93%	95%
**Patient status: Perpetrator**	89%	89%	89%	85%
**Patient status: Victim**	91%	89%	91%	90%
**Violence type: Domestic**	94%	94%	94%	93%
**Violence type: Physical**	91%	92%	91%	98%
**Violence type: Sexual**	98%	97%	97%	93%

Table 3: Proportion of each label in the annotation dataset – affirmed or negated/irrelevant. Each text extract was first annotated for the violence presence label, then if this was affirmed, further annotated for the other labels related to patient status and violence type (see figure 1 for further details). Therefore denominator totals for the violence presence label is larger than that for the other labels.

Annotation label	Affirmed, N (%)	Negated or irrelevant, N (%)	Total
**Violence presence**	2199 (58)	1572 (42)	3771
**Patient status: Perpetrator**	1350 (61)	849 (39)	2199
**Patient status: Victim**	731 (33)	1468 (67)	2199
**Violence type: Domestic**	723 (33)	1476 (67)	2199
**Violence type: Physical**	1724 (78)	475 (22)	2199
**Violence type: Sexual**	353 (16)	1846 (84)	2199

Table 3 shows the breakdown of annotation labels across the annotated dataset at an annotation level. Overall, 58% (2,199) of the text extracts were affirmed for violence presence. Of these, the proportion of affirmed examples for patient status ranged from 33% (victim) to 61% (perpetrator), and for violence type from 16% (sexual) to 78% (physical). Some affirmed labels overlapped, because a fragment of text could have contained multiple references to different types of violence (e.g. both physical and sexual violence) and different patient statuses (e.g. as both perpetrator and victim). Table 4 describes these overlaps. For instance, the number of examples where *perpetrator* was affirmed (n=1350) overlapped with affirmed for *physical* violence in almost 80% of the examples (n=1078), while the overlap with *victim* was rarer (8%, n=113).

Table 4: Overlap of labels present in affirmed annotations, showing the number and percentage of annotations that shared different labels.

For peer review only

	Perpetrator, N (%)	Victim, N (%)	Sexual, N (%)	Physical, N (%)
**Perpetrator**	-	-	-	-
**Victim**	113 (8.4)	-	-	-
**Sexual**	150 (11.1)	199 (27.2)	-	-
**Physical**	1078 (79.9)	616 (84.3)	304 (86.1)	-
**Domestic**	331 (24.5)	318 (43.5)	104 (29.5)	593 (34.4)
Column total	*1350 (100.0)*	*731 (100.0)*	*353 (100.0)*	*1724 (100.0)*

Table 5 and Table 6 show the model-to-annotator agreement and a model error analysis respectively. Both measures indicate high agreement between the models and annotators.

Table 5: Kappa agreement between manually and automatically assigned categories in training and testing sets.

Annotation label	Model-to-annotator agreement
**Violence presence**	98.1%
**Patient status: Perpetrator**	97.4%
**Patient status: Victim**	96.2%
**Violence type: Domestic**	98.7%
**Violence type: Physical**	98.3%
**Violence type: Sexual**	96.8%

Table 6: Model error analysis on training and testing sets

Annotation label	Model error analysis	False positives
**Violence presence**	34	24
**Patient status: Perpetrator**	47	35

For peer review only

**Patient status: Victim**	45	5
**Violence type: Domestic**	15	10
**Violence type: Physical**	34	7
**Violence type: Sexual**	21	11

Demographic features of the patients included in the annotated dataset sample are presented in Table 7. The sample represented in total 2,832 patients, of whom 57% were female, 45% were aged 40-60 at the time the fragment was extracted, 66% were single, 52% were of White ethnic background, and 36% were diagnosed with psychotic disorders (ICD-10 codes F20-29) [33]. This NLP approach captures references to violence occurring in a large body of patients presenting to a mental health service who are not necessarily presenting for violence-related reasons.

Table 7: Characteristics of patients whose text extracts were annotated as part of this study.

		**Frequency, N (%)**
**Age**		
	**<20**	167 (5.9)
	**20<40**	791 (27.9)
	**40<60**	1273 (45.0)
	**60<80**	458 (16.2)
	**80<**	141 (5.0)
	**Missing**	2 (0.1)
**Gender**		
	**Male**	1216 (42.9)
	**Female**	1614 (57.0)
	**Missing**	2 (0.1)
**Marital status**		
	**Single**	1865 (65.9)
	**Married/cohabiting**	344 (12.2)
	**Divorced/separated**	262 (9.3)
	**Widowed**	85 (3.0)

For peer review only

Missing	276 (9.8)
**Ethnicity**	
White	1482 (52.3)
Black	104 (3.7)
Asian	160 (5.7)
Mixed	885 (31.3)
Other	90 (3.2)
Missing	111 (3.9)
**ICD-10 Diagnosis**	
F0-9: Organic, including symptomatic, mental disorders	185 (6.5)
F10-19: Mental and behavioural disorders due to psychoactive substance use	94 (3.3)
F20-29: Schizophrenia, schizotypal and delusional disorders	1031 (36.4)
F30-39: Mood [affective] disorders	451 (15.9)
F40-49: Neurotic, stress-related and somatoform disorders	203 (7.2)
F50-59: Behavioural syndromes associated with physiological disturbances and physical factors	18 (0.6)
F60-69: Disorders of adult personality and behaviour	236 (8.3)
F70-79: Mental retardation	53 (1.9)
F80-89: Disorders of psychological development	98 (3.5)
F90-99: Behavioural and emotional disorders with onset usually occurring in	211 (7.6)

For peer review only

childhood and adolescence and unspecified mental disorder	
**No axis 1 diagnosis**	25 (0.9)
**G: Diseases of the nervous system, X: Intentional self-harm, assault or Z: Factors influencing health status and contact with health services***	163 (5.8)
**Missing**	64 (2.3)
**Total**	**2832** (100.0)

*ICD-10 categories G and X were combined with ICD-10 category Z due to small numbers of participants (n<10) in these categories, in order to limit identification of participants.

Discussion

To our knowledge, this is the first study that has used a NLP approach to code free-text data from a large and diverse source of electronic mental health records to ascertain violence according to presence, agent and type. There is limited previous research examining NLP to extract violence-related information in EHRs but includes utilising text rules to ascertain violent behaviour as antecedents to supervised confinement [16] and the employment of a bag of words machine learning approach to extract information on physical assault victimisation in CRIS data [28]. Our approach captures a much broader range of experiences. We successfully developed an annotated dataset of clinical text references to interpersonal violence, using a multidisciplinary clinical academic group. We used this dataset to develop binary classification NLP models for extracting and classifying clinical text fragments referring to interpersonal violence in mental health EHRs, including for patient status (perpetrator, victim) and violence type (domestic, physical, sexual). Models were developed with a state-of-the-art NLP algorithm (fine-tuned BioBERT) and displayed very good performance based on accepted evaluation criteria. A planned NLP model for extracting references to witnessed violence was not successfully developed due to an insufficient sample size.

This study had several limitations that need to be borne in mind when interpreting the findings. Although agreement between annotators was generally good, some disagreements occurred. Examples of disagreements included ascertaining the status of the patient as perpetrator/victim/witness in a text fragment and whether “fighting” was considered

interpersonal violence or a colloquial term for verbal arguments. As our focus was on interpersonal violence, we did not annotate or develop models for individuals forced to fight in armed conflict, for example. Keywords used in this study were selected to capture as many instances of interpersonal violence in clinical text as possible but may not have captured all violence categories of “hidden violence”. For instance, keywords related to female genital mutilation, forced marriage, trafficking, neglect, sensory deprivation, harrassment, stalking, or reproductive coercion and control were not included, and would need separate analysis for NLP development. These specific forms of violence could, however, be readily addressed using an “add-on” to the approach presented here. Similarly, emotional and psychological violence were not included in the list of keywords, and capturing these characteristics through NLP is likely to be more complex because of the broader way in which this is likely to be described in clinical text. Annotation was carried out on the basis of the meaning and sense of the text fragments sample in this study, rather than entire EHR documents. It is possible that annotations based on entire documents would have delivered slightly different results, but this would have been challenging to implement given the quantity of documents that would be needed to be manually labeled in order to capture enough examples. Lastly, we present an NLP approach to extracting clinical text fragments which refer to violence from mental health records. Given the sufficiently accurate performance reported in this study, this approach can extract references to violence where it is written down in clinical records, but is restricted to these recorded instances, and cannot be considered a method for measuring prevalence of all experienced violence (without further assumptions which are likely to depend on the situation). This is likely to continue to require asking patients themselves, or linking data from other sources such as hospital data or crime records. Indeed, these approaches could be helpful in understanding the processes by which violence is identified by clinicians and recorded in clinical notes.

This study had some specific advantages. Annotations were derived from rich and diverse free-text data from service-users’ clinical notes, as opposed to structured data. The data are derived from progress notes entered by wide array of clinical groups and professionals, therefore increasing the chances of detecting violence information. Furthermore, given the longitudinal nature of our dataset, information was recorded over a sixteen-year period (2007-2019), which further increases our ability to detect experiences of violence, if recorded in the clinical notes. All annotations were coded using human annotators, with high inter-annotator agreement, which support the robustness of our approach. Lastly, we used a state-of-the-art NLP pre-trained transformer model, BioBERT, which has been shown to outperform methods more traditionally used for symptoms detection such as support vector machine [26] and this allowed us to develop fine-tuned

For peer review only

models with very promising results. The BioBERT model is readily deployable and interpretable, with all scripts made publicly available on GitHub. BioBERT fine-tuning does typically not need significant computer resources. The fine-tuned model is relatively light (450Mb) and easy to deploy in clinical settings.

NLP offers a reliable, automated, scalable approach to extracting summary information from EHRs. Reasonably accurate methods for extracting clinical text referring to interpersonal violence can support further research on correlates of clinically-identified violence, and how professionals and services respond to violence. Evaluation of NLP-derived violence indicators in relation to linked external data on hospital admissions, GP registers, and crimes data could allow some assessment of the reliability with which clinicians record violence when it occurs. Based on the inter-operability of NLP algorithms across different EHR formats, models developed in this study could be feasibly applied to mental health EHRs in other sites (including CRIS databases elsewhere in the UK) and other free-text containing EHRs. The main limitation to porting this model to other sites would be differences in language that may lead to a slightly different list of keywords. However, the current list already captures many common terms related to violence and was reviewed by clinicians that work in several NHS Trusts to ensure generalisability. Furthermore, we have developed an NLP tool that is easily adaptable and allows quick fine-tuning and deployment of the model if needed.

There is also a significant clinical need for methods which can accurately summarise relevant historic information for clinicians to review and use in decision making at the point of care. This is particularly relevant in the arena of violence reduction and mental health, where serious incident reviews and charitable organizations have called for renewed attention to the need for accurate summaries of previous violence, and improved information sharing with other agencies [34]. By summarising previous exposure to violence, clinical information systems might improve the efficiency of clinical encounters, e.g. by reducing clinical time taken up with collecting information on previous violence. Future research might also consider the clinical benefits of accurate summaries of previous interpersonal violence which might be reported to treating clinicians in real time, to aid decision making. We suggest that attention is warranted into the ethical and regulatory challenges of translating NLP methods for violence into practice.

We were unable to model witnessing violence using NLP in this study. This could form the focus of future research based on the possible impact of witnessing violence on health [35]. The infrequency of witnessed violence during the annotation process may reflect that clinicians are not frequently enquiring about these experiences, or they are not being recorded.

For peer review only

We did not consider temporality of violence mentions, but this is something that we hope to integrate in future to improve the usability of the models.

Conclusion

We have demonstrated that it is possible to use state-of-the-art NLP methods to extract clinical text referring to violence (including distinguishing patients as perpetrators and victims, as well as violence types such as physical, domestic, and sexual) at scale with acceptable accuracy in mental health EHRs. This could support further research into the pathways by which violence is identified in clinical practice, and the effectiveness of systems for identifying, assessing and managing interpersonal violence. However, while NLP approaches might offer a sufficiently accurate method for summarising violence-related information to aid clinical decision making, considerable ethical and regulatory questions remain.

Author contributions:

RB drafted the first version of the manuscript. RB and MW annotated the dataset. AM developed the NLP models. VB, GKS, RS provided clinical and epidemiological input in relation to the motivation for the work, annotation guideline development and definitions. SV and AR provided technical input and guidance in relation to the NLP model development. All authors drafted and/or critically revised the manuscript and approved the final version of the manuscript.

Ethics Statement:

The CRIS database has received ethical approval for secondary analysis: Oxford REC C, reference 18/SC/0372. The CRIS database was developed with extensive service user involvement and adheres to strict governance frameworks managed by service users. Data are used in an entirely de-identified and secure format and all patients have the choice to opt-out of their de-identified data being used.

For peer review only

References

1 Krug EG, Mercy JA, Dahlberg LL, *et al.* The world report on violence and health. *The Lancet* 2002;**360**:1083–8. doi:10.1016/S0140-6736(02)11133-0

2 Reza A, Mercy JA, Krug E. Epidemiology of violent deaths in the world. *Injury Prevention* 2001;**7**:104–11. doi:10.1136/ip.7.2.104

3 Olofsson N, Lindqvist K, Shaw BA, *et al.* Long-term health consequences of violence exposure in adolescence: a 26–year prospective study. *BMC Public Health* 2012;**12**:411. doi:10.1186/1471-2458-12-411

4 López-Martínez AE, Serrano-Ibáñez ER, Ruiz-Párraga GT, *et al.* Physical Health Consequences of Interpersonal Trauma: A Systematic Review of the Role of Psychological Variables. *Trauma, Violence, & Abuse* 2018;**19**:305–22. doi:10.1177/1524838016659488

5 Maniglio R. Severe mental illness and criminal victimization: a systematic review. *Acta Psychiatrica Scandinavica* 2009;**119**:180–91. doi:https://doi.org/10.1111/j.1600-0447.2008.01300.x

6 Khalifeh H, Moran P, Borschmann R, *et al.* Domestic and sexual violence against patients with severe mental illness. *Psychol Med* 2015;**45**:875–86. doi:10.1017/S0033291714001962

7 Khalifeh H, Johnson S, Howard LM, *et al.* Violent and non-violent crime against adults with severe mental illness. *British Journal of Psychiatry* 2015;**206**:275–82. doi:10.1192/bjp.bp.114.147843

8 Fazel S, Gulati G, Linsell L, *et al.* Schizophrenia and violence: Systematic review and meta-analysis. *PLoS Medicine* 2009;**6**. doi:10.1371/journal.pmed.1000120

9 Kadra G, Dean K, Hotopf M, *et al.* Investigating Exposure to Violence and Mental Health in a Diverse Urban Community Sample: Data from the South East London Community Health (SELCoH) Survey. *PLOS ONE* 2014;**9**:e93660. doi:10.1371/journal.pone.0093660

10 Bhavsar V, Dean K, Hatch SL, *et al.* Psychiatric symptoms and risk of victimisation: a population-based study from Southeast London. *Epidemiology and Psychiatric Sciences* 2019;**28**:168–78. doi:10.1017/S2045796018000537

11 Resnick HS, Acierno R, Kilpatrick DG. Health impact of interpersonal violence. 2: Medical and mental health outcomes. *Behav Med* 1997;**23**:65–78. doi:10.1080/08964289709596730

12 Raune D, Bebbington P, Dunn G, *et al.* Event attributes and the content of psychotic experiences in first-episode psychosis. *PSYCHOL MED* 2006;**36**:221–30.

13 Flood C, Bowers L, Parkin D. Estimating the costs of conflict and containment on adult acute inpatient psychiatric wards. *Nurs Econ* 2008;**26**:325–30, 324.

14 Walby S. Improving the statistics on violence against women. *Statistical Journal of the United Nations ECE* 2005;**22**:193–216. doi:10.3233/SJU-2005-223-402

15 Robson D, Spaducci G, McNeill A, *et al.* Effect of implementation of a smoke-free policy on physical violence in a psychiatric inpatient setting: an interrupted time series analysis. *The Lancet Psychiatry* 2017;**4**:540–6. doi:10.1016/S2215-0366(17)30209-2

16 Cullen AE, Bowers L, Khondoker M, *et al.* Factors associated with use of psychiatric intensive care and seclusion in adult inpatient mental health services. *Epidemiology and Psychiatric Sciences* 2018;**27**:51–61. doi:10.1017/S2045796016000731

17 Ford E, Carroll JA, Smith HE, *et al.* Extracting information from the text of electronic medical records to improve case detection: a systematic review. *Journal of the American Medical Informatics Association* 2016;**23**:1007–15. doi:10.1093/jamia/ocv180

For peer review only

18 Kreimeyer K, Foster M, Pandey A, *et al.* Natural language processing systems for capturing and standardizing unstructured clinical information: A systematic review. *Journal of Biomedical Informatics* 2017;**73**:14–29. doi:10.1016/j.jbi.2017.07.012
19 Wang Y, Wang L, Rastegar-Mojarad M, *et al.* Clinical information extraction applications: A literature review. *Journal of Biomedical Informatics* 2018;**77**:34–49. doi:10.1016/j.jbi.2017.11.011
20 Friedman C, Alderson PO, Austin JH, *et al.* A general natural-language text processor for clinical radiology. *J Am Med Inform Assoc* 1994;**1**:161–74.
21 Savova GK, Masanz JJ, Ogren PV, *et al.* Mayo clinical Text Analysis and Knowledge Extraction System (cTAKES): architecture, component evaluation and applications. *J Am Med Inform Assoc* 2010;**17**:507–13. doi:10.1136/jamia.2009.001560
22 Soysal E, Wang J, Jiang M, *et al.* CLAMP – a toolkit for efficiently building customized clinical natural language processing pipelines. *J Am Med Inform Assoc* 2017;**25**:331–6. doi:10.1093/jamia/ocx132
23 Wu H, Hodgson K, Dyson S, *et al.* Efficient Reuse of Natural Language Processing Models for Phenotype-Mention Identification in Free-text Electronic Medical Records: A Phenotype Embedding Approach. *JMIR Med Inform* 2019;**7**:e14782. doi:10.2196/14782
24 Devlin J, Chang M-W, Lee K, *et al.* BERT: Pre-training of Deep Bidirectional Transformers for Language Understanding. In: *Proceedings of the 2019 Conference of the North American Chapter of the Association for Computational Linguistics: Human Language Technologies, Volume 1 (Long and Short Papers)*. Minneapolis, Minnesota: : Association for Computational Linguistics 2019. 4171–86. doi:10.18653/v1/N19-1423
25 Lee J, Yoon W, Kim S, *et al.* BioBERT: a pre-trained biomedical language representation model for biomedical text mining. *Bioinformatics* 2020;**36**:1234–40. doi:10.1093/bioinformatics/btz682
26 Mascio A, Kraljevic Z, Bean D, *et al.* Comparative Analysis of Text Classification Approaches in Electronic Health Records. *arXiv:200506624 [cs]* Published Online First: 8 May 2020.http://arxiv.org/abs/2005.06624 (accessed 18 Jan 2021).
27 Si Y, Wang J, Xu H, *et al.* Enhancing clinical concept extraction with contextual embeddings. *Journal of the American Medical Informatics Association : JAMIA* 2019;**26**. doi:10.1093/jamia/ocz096
28 Bhavsar V, Sanyal J, Patel R, *et al.* The association between neighbourhood characteristics and physical victimisation in men and women with mental disorders. *BJPsych open* 2020;**6**:e73. doi:10.1192/bjo.2020.52
29 Jackson RG, Patel R, Jayatilleke N, *et al.* Natural language processing to extract symptoms of severe mental illness from clinical text: the Clinical Record Interactive Search Comprehensive Data Extraction (CRIS-CODE) project. *BMJ Open* 2017;**7**:e012012. doi:10.1136/bmjopen-2016-012012
30 Viani N, Botelle R, Kerwin J, *et al.* A natural language processing approach for identifying temporal disease onset information from mental healthcare text. *Scientific Reports* 2021;**11**:757. doi:10.1038/s41598-020-80457-0
31 Perera G, Broadbent M, Callard F, *et al.* Cohort profile of the South London and Maudsley NHS Foundation Trust Biomedical Research Centre (SLaM BRC) Case Register: current status and recent enhancement of an Electronic Mental Health Record-derived data resource. *BMJ Open* 2016;**6**:e008721. doi:10.1136/bmjopen-2015-008721
32 Clinical Record Interactive Search (CRIS). https://www.slam.nhs.uk/quality-and-research/clinical-record-interactive-search-cris/ (accessed 2 Feb 2021).

For peer review only

33 World Health Organization, editor. *International statistical classification of diseases and related health problems*. 10th revision, 2nd edition. Geneva: : World Health Organization 2004.

34 Effectiveness of anonymised information sharing and use in health service, police, and local government partnership for preventing violence related injury: experimental study and time series analysis | The BMJ. https://www.bmj.com/content/342/bmj.d3313 (accessed 28 Jan 2021).

35 Clark C, Ryan L, Kawachi I, *et al.* Witnessing Community Violence in Residential Neighborhoods: A Mental Health Hazard for Urban Women. *J Urban Health* 2008;**85**:22–38. doi:10.1007/s11524-007-9229-8

Figure legends:

Figure 1: Process of annotation, development and evaluation of NLP models

Figure 2: Flowchart of extract annotation process

For peer review only

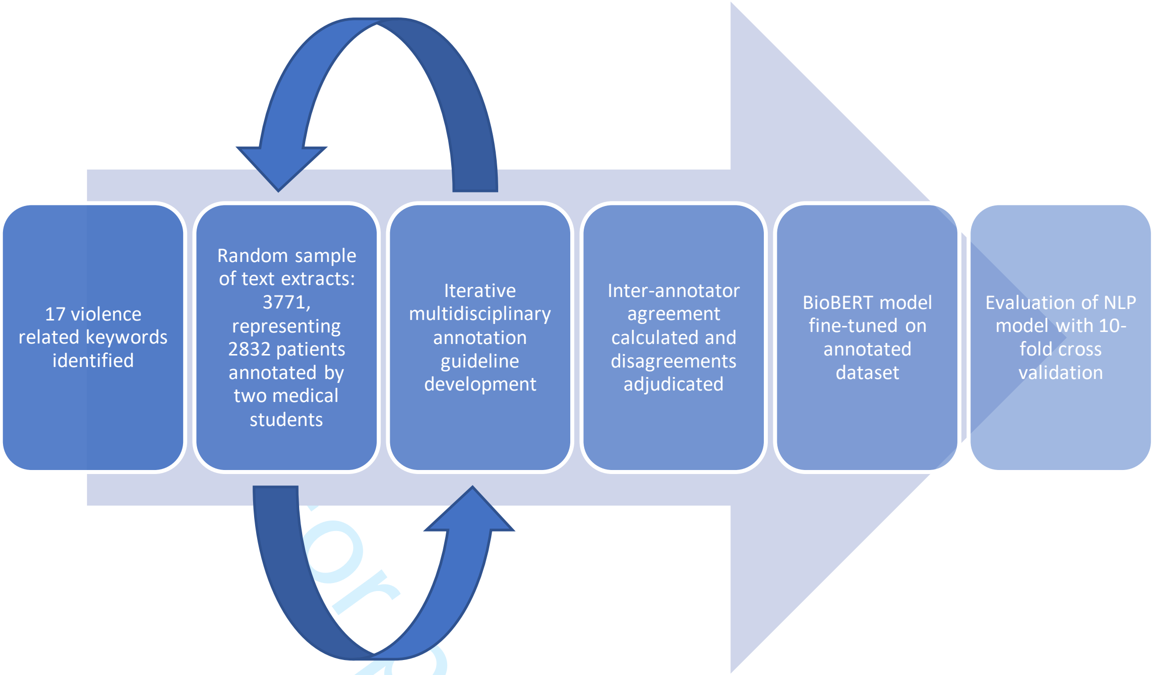

For peer review only

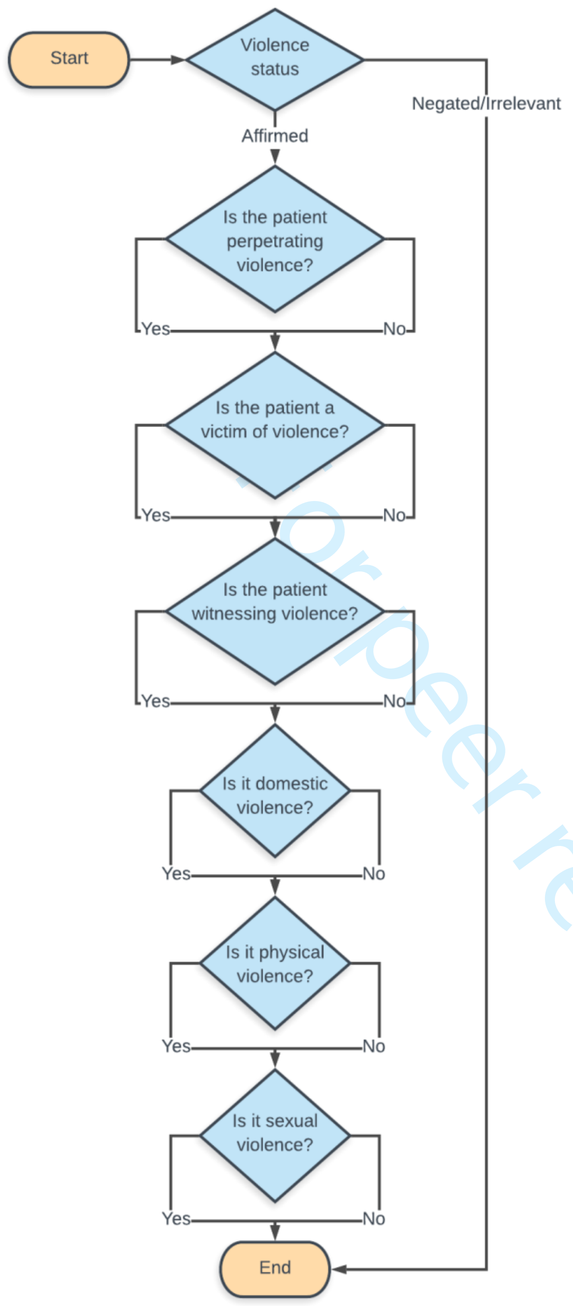

Figure 2: Flowchart of extract annotation process

Appendix 1

Keywords

Nouns

% abus%
% attack%
% beat%
% violenc%
% hit%
% rape%
% assault%

Verbs:

% fight%
% fought%
% slap%
% chok%
% push%
% punch%
% strangul%
% strangl%
% threw%
% struck%

For peer review only

Appendix 2: Guidelines

Guidance for violence annotations

Development team: Aurelie Mascio, Sumithra Velupillai, Riley Botelle, Marcus Williams, Rob Stewart, Vishal Bhavsar, Giouliana Kadra

Annotations were made for:

a) *violence* status: **affirmed** or negated or irrelevant. When annotated as affirmed, further annotations were made for:

b) *patient* status: perpetrator and/or victim and/or witness and/or other, and

c) violence *type*: physical and/or sexual and/or domestic (or none of the above).

NOTE: there was not a sufficient amount of examples to develop a robust classification model for *witness*, thus there is no corresponding NLP application for this category.

For peer review only

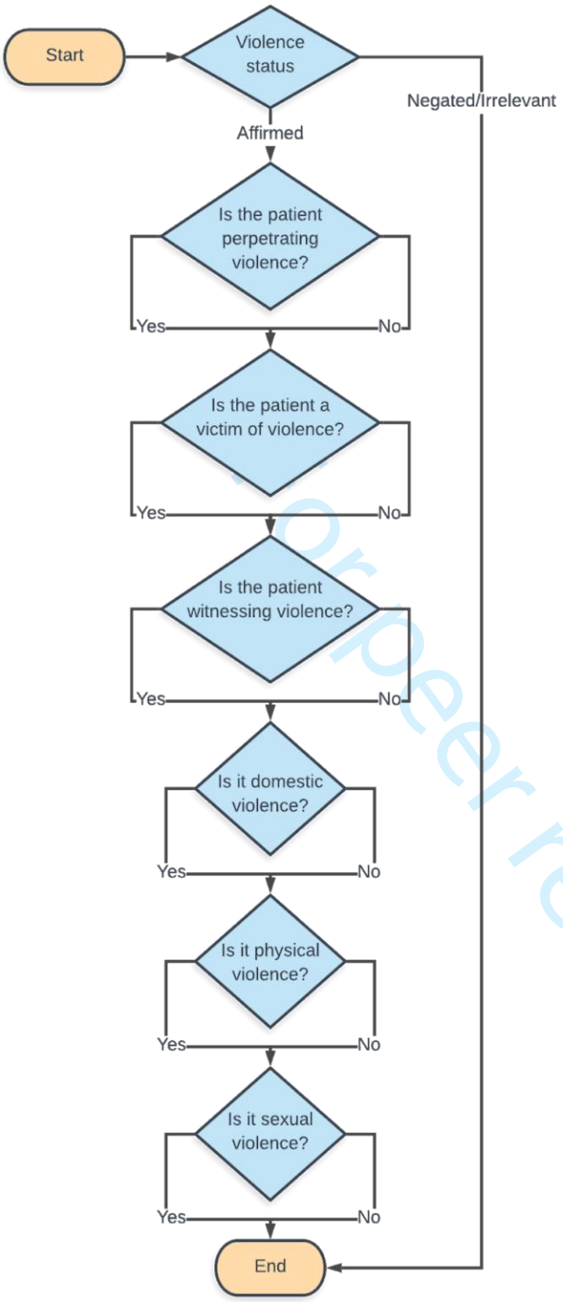

Violence status

Definition

Violence is the use of physical force or power, threatened or actual, against another person that results in - or has a high likelihood of resulting in - injury, death, psychological harm, maldevelopment, or deprivation. This does not just cover physical violence, but also violent threats, neglect, racial, digital, sexual abuse, financial abuse and coercion and control.

Our definitions of violence and sexual violence largely align with the WHO definition of violence (see also: WHO 2002 report), but with the criteria for intentionality removed due to an inability to determine this in most extracts. The violence definitions are specifically interpersonal, rather than including violence to self.

Annotation examples and criteria:

For peer review only

Violence Status Decisions		
**Examples**	**Classification**	**Explanation**
"They were *abused* in their childhood"	Affirmed	
"His brother would *beat* him up if he found out", "risk of *violence*"	Irrelevant	**Hypothetical scenarios** are irrelevant
"Expressed a lot of interest in *violence*, nazism"	Irrelevant	**Fantasies and ideological violence** is irrelevant
"Patient is *hitting* himself"	Irrelevant	**Self injury** is irrelevant
"Patient is *abusive* towards their pet"	Irrelevant	**Violence towards animals** is irrelevant
"Patient shows *violent* behaviour such as breaking objects and punching walls"	Irrelevant	**Violence directed towards objects**/property is irrelevant, affirmed only if clear intention to cause harm or fear to others.
"I have enclosed a leaflet for sexual abuse services" "referred to the domestic *violence* advisor"	Irrelevant	**Referrals** are irrelevant.
Learning difficulties/REM sleep behaviour disorder/violence without intent to harm	Affirmed	Violence is affirmed regardless of **intent**.
"Acquitted as accomplice to gang *rape* (negated) . . . convicted of ABH (affirmed)"	See explanation	When there are **multiple incidents** within the text use the keyword to determine if negated or affirmed.
"Denied the allegations he *hit* members of staff" "Questioned by police recently for allegedly *hit*ting his ex-girlfriend however he denied he was present." "Allegation made by patient of repeated sexual *assault* . . . on detailed questioning the patient denied being repeatedly assaulted"	See explanation	Denying incident = negated<br>Allegation = affirmed<br>Allegation and denial (**contrasting claims**) = affirmed<br>Not guilty = negated<br>Charges dropped = affirmed<br>Acquitted = negated<br>If confusing or unsure mark as irrelevant
"He told his mother he plans to hit them with a baseball bat"<br>"He made a gesture to attack staff"	Affirmed	**Plans** for violence are affirmed, similarly gestures or 'appearances' of violence are affirmed
"Patient was *abus*ing staff verbally" "patient made threats to kill and *attack* staff"	Affirmed	**Verbal/threats** of violence are affirmed as violence status

		(but are not affirmed as physical violence, see below?)
"No *violence* or aggression noted"	Negated	
"Found the way they were brought to the hospital very traumatic and therefore felt very *abus*ed by mental health services"	Irrelevant	**Institutional and structural violence** is irrelevant. Interpersonal violence by individuals within institutions (e.g. beaten by police, abused by prison guards) would still be affirmed
"she repeatedly called the police making allegations of being posioned on the ward . . . she also made allegations of physical and sexual *abuse* by patients and staff" "the voices . . . threaten to harm her and sometimes she feels them *hit* her"	Affirmed or Irrelevant	For allegations where the patient is clearly recorded as **hallucinating or delusional** within the fragment, mark as irrelevant. If unclear if delusional or a real event, mark as affirmed.
"Forced to *fight*" (in a war/army), "was not tortured but witnessed dead bodies and forced to move frequently due to *fight*ing"	Irrelevant	**Military service** should not be captured - irrelevant Collective violence should not be captured - irrelevant
"Suffered financial abuse"	Affirmed	Financial, psychological, emotional and **other types of abuse** including deprivation and neglect are affirmed.
"He has a history of *violence*" "patient used to *hit* her partner" "Is not as verbally *abus*ive as she used to be"	Affirmed	**Historical abuse** is affirmed.

Patient status

Definition

The **perpetrator** is the person using physical force or power, the **victim** is the person this is used against. A **witness** is someone who directly observes acts of violence through sight and/or sound.

Annotation examples and criteria:

**Patient Status Decisions**		
**Examples**	**Classification**	**Explanation**
"he stabbed someone who had *attack*ed him",	Affirmed, perpetrator	Patient status may be any combination of victim and perpetrator and witness.

"Arrested following violence and fire setting . . . having been raped 3 years ago"	and/or victim and/or witness	
"They often argued and fought", "got into a fight", "became involved in a fight", "they had fought"	See explanation	Generally for vague **"involvement" in fights** patient status should be marked as "other". If there is clear initiation of the fight they would be the perpetrator, if there is explicit harm or intended harm to a party they would be the victim or perpetrator accordingly.
"tried to rob him . . . he *fought* back and they fled", "talked about his brother bullying him as a child until he *fought* back", "he felt another youth was threatening to him and *fought* back violently"	Perpetrator and victim	Where there is **retaliatory violence or self defence** mark the patient as both perpetrator and victim
"his son got *beat* up on the way home from school"	Other	**Indirect witnesses** or where it is unclear if they saw/heard/witnessed themselves should be marked as other.
"Watched his father being killed"	Witness	Clearly **direct witnesses** should be marked as witness.

Violence type

Definition

Domestic violence is violence between family members, intimate partners, ex-intimate partners and household members.

Physical violence is violence that uses physical force, or results in or has a high likelihood of resulting in physical injury to the victim.

Sexual violence includes any unwanted sexual act, unwanted sexual comments or advances, unwanted attempts to obtain a sexual act, directed acts against a person's sexuality using coercion and acts to traffic. It includes (but is not limited to) rape, sexual harrassment, sexual assault, forced marriage, FGM, and reproductive coercion and control.

Domestic violence is based on the UK government domestic abuse statutory guidance framework 2020, but adjusted to be applicable to all ages (rather than being restricted to over 16s) and includes people who are household members through choice (but not those in institutional, residential, detention or in-patient settings). Thresholds for considering violence physical are partially adapted from UK law, for instance "offences against the person" (1861 Act) describes crimes which are committed by direct physical harm or force and includes spitting as a form of assault and poisoning as a non-fatal, non-sexual offence.

Annotation examples and criteria:

For peer review only

Violence Type Decisions		
**Examples**	**Classification**	**Explanation**
**Violence Type Decisions (Physical)**		
"physical discipline" which was not perceived though as physical *abuse.*" "hit or made to kneel by father . . . did not see this as *abus*ive"	See explanation	Varies on keyword - these would be negated based on the negation of "abusive" keyword, but affirmed if the keyword was "physical discipline" or "hit".
"false imprisonment"/"abduction"	Physical = yes	
"spitting"	Physical = yes	Legally considered physical assault
"spiked" (drink) or poisoning	Physical = yes	Legally considered assault by poisoning
"Tried to *hit*", "Had to be stopped from *assaulting*"	Physical = yes	**Attempts to inflict physical violence** should be marked as physical
"*Rape*", "sexual *assault*"	Physical = yes, sexual = yes	**Physical sexual violence** should be marked as both physical and sexual
**Violence Type Decisions (Domestic)**		
"Partner's son", "brother's girlfriend", "father"	Domestic = yes	**Familial relationships** (biological or adoptive) are considered domestic
"patient stabbed his roommate"	Domestic = yes	Those living together by choice (**households**) are domestic e.g. flatmates and cohabitees
"*abuse* she suffered whilst in care"	Domestic = no	**Residential and institutional settings** are not domestic including inpatient wards, prisons and care homes
"Hitting her ex-girlfriend"	Domestic = yes	**Partners and ex-partners** are considered domestic
**Violence Type Decisions (Sexual)**		

For peer review only

Sexual harassment	Sexual = yes, physical = no	**Sexual harrassment** is considered to be sexual violence.
“talked to female staff about his sexual fantasies”, “indecent exposure”	Sexual = yes	Mark as sexual violence if interpersonal (e.g. directed at or caused harm to another person)
“Patient was sexually disinhibited”, “Patient showed sexually inappropriate behaviour”	Irrelevant	Mark as irrelevant if not person-directed or no clear harm to another person.

For peer review only

Appendix 3: Guideline development discussion

Definitions of violence

An affirmed instance of violence - what should this mean?

Our NLP definition: Violence is the use of physical force or power, threatened or actual, against another person that results in - or has a high likelihood of resulting in - harm.

Harm in this instance does not refer only to physical injury, but also covers abuses of power such as violent threats and intimidation, acts which result in emotional and psychological harm, neglect, sexual abuse, financial abuse and coercion and control. It does not include violence against the self.

This definition was developed from the WHO definition of violence (Krug, 2002), but adjusted using deductive reasoning based on the annotated extracts and guideline development discussions, and therefore differs in two key ways. The first is that it only identifies interpersonal violence, and the second is that it does not require the violence to be intentional in order to be affirmed. These two changes are because there are various limitations to how violence can be affirmed in a way that can be applied reliably to EHR data.

It must be interpersonal because violence that is perpetrated systemically, state violence and structural violence is harder to identify, and tends to be poorly recorded. For example the quotation that a patient "found the way they were brought to the hospital very traumatic and therefore felt very *abus*ed by mental health services" is marked as irrelevant in this approach, as there is no identifiable interpersonal violence, however "*beat*en by prison guards" would be affirmed. Violence against whole communities is also poorly captured without an interpersonal element. We chose not to include self-directed violence.

This definition excludes acts of violence that are hypothetical ("risk of *violence*") and those that are fantasies or ideological ("expressed a lot of interest in *violence*, nazism"). It includes plans for violence ("he told his mother he plans to *hit* them with a baseball bat") and attempts made at violence which are thwarted ("had to be stopped from *assaulting*"). Whilst traditional definitions of violence place importance on the concept of intentionality, in EHRs generally the data recorded is of incidents and histories, not motivations. Intentions may be repressed, denied, disagreed upon or not recorded at all. Therefore whilst intentionality is considered part of violence and hence plans and attempts are affirmed, it was considered not necessary for intentionality to be clear in order for the instance of violence to be affirmed in the NLP approach.

Annotation was additionally complicated by the mental status of patients and resultantly the reliability of the annotators (or even the original record authors) to determine whether a patient is hallucinating or delusional when reporting a violent event. A violent event cannot be assumed to be false because of its perceived improbability, and even when some aspects are impossible other aspects may be part of a real experience. Guidelines on this must choose whether to err on the side of affirming violence that did not happen against the risk of misassigning true experiences of patient-reported violence as irrelevant. The problem with the latter is that it may create a situation where patients with delusional or hallucinatory experiences are less likely to have instances of violence identified, and we considered that the resultant harms from this outweighed the overestimation of violence by including some false events. For instance, the example "she repeatedly called the police making allegations

For peer review only - http://bmjopen.bmj.com/site/about/guidelines.xhtml

of being posioned on the ward . . . she also made allegations of physical and sexual *abuse* by patients and staff" was not be assumed to be a delusional experience, as this risked masking real violent events. Contrastingly however "the voices . . . threaten to harm her and sometimes she feels them *hit* her" is explicitly a hallucinatory experience, and therefore not interpersonal and not affirmed.

Other difficulties with identifying violence reflect the entanglement of multiple viewpoints within the text. How should we classify a violent act that the author is asserting happened but the patient denies? Or similarly, an allegation by one party and a denial by another in the same text, or even an allegation and later denial by the same patient? (For instance, "allegation made by patient of repeated sexual *assault* . . . on detailed questioning the patient denied being repeatedly assaulted.") How should legal terms be interpreted - someone who is charged, or acquitted, or who's case against them is dropped? Our approach tended towards affirming the identification of violent acts where there was a degree of uncertainty or disagreement as to whether they occurred or not, and only excluding those where everyone was in agreement there had been no violence, for the same concerns about harms resulting from the classification of reported violence as false. This overall approach should lend itself to an output where the application is more likely to produce false positives than false negatives.

Thresholds for considering something as violence could also be challenging. Our definition includes actions or inaction that results in harm, but what may cause harm for one person may not cause harm to others. As a general rule the guidelines do not include violent acts against property or objects as these are not interpersonal, but destroying a valued object to punish someone or throwing something in order to incite fear are both interpersonal violent acts that should be identified and affirmed. Violence towards animals was similarly excluded from the definition for not being interpersonal. There was occasionally difficulty in differentiating between aggressive behaviour and violence. The majority of aggressive behaviour, if directed at another person, fulfils the definition of violence and was appropriately accounted for by these guidelines.

Defining patient status - who did what?

Our NLP definition: The **perpetrator** is the person using physical force or power, the **victim** is the person this is used against. A **witness** is someone who directly observes acts of violence through sight and/or sound.

Identifying the role of the patient (presupposing you can identify who the patient is in the text) was on average a straightforward process, and usually explicitly stated. There are two exceptions we found to this; instances where there was some level of mutuality in the violence, and instances where the violence was retaliatory or undertaken as self-defence. To resolve these problems, patients could be assigned both as the perpetrator and the victim in scenarios where they both enacted and received violence. It has been argued that self-defence should not be considered violence (Hamby, 2017) but extracts from EHRs often included disagreement as to whether an event was truly self-defence or not, and thus following the rules for disagreements between people or the authors of the texts, were marked as affirmed.

Types of violence

For peer review only

Our NLP definition of physical violence is violence that uses physical force, or results in or has a high likelihood of resulting in, physical injury to the victim.

Where there was uncertainty about whether an act should be considered physical violence, such as spitting and poisoning, an exemplar approach was utilised. These utilised categorisations adapted from UK law such as "offences against the person" (1861 Act) which describes crimes which are committed by direct physical harm or force and includes spitting as a form of assault and poisoning as a form of non-fatal, non-sexual offence. False imprisonment and abduction were agreed by discussion to be a form of physical violence due to the physically restricting nature of the act.

One difficulty with establishing whether physical violence should be affirmed is with acts of physical violence that were not considered to result in harm by the victim, such as "hit or made to kneel by father . . . did not see this as *abus*ive". There are two potential approaches here; either choosing which to prioritise between actions and harms, or using the specific keywords to make two separate judgements. With the latter approach, the example would appear twice, once as affirmed for "hit" and once as negated for "abusive".

Our NLP definition of domestic violence is violence between family members, intimate partners, ex-intimate partners and household members.

Our definition of domestic violence is designed to capture a relational aspect between the victim and perpetrator; that is it represents a family, intimate partner and/or domestic (household) relationship.

Some legal definitions of domestic violence (e.g. South African Domestic Violence Act 1998) include housemates and flatmates who share or have recently shared the same residence, whereas others only refer to previous or current intimate partners and family members. Our definition, whilst including household members, only includes those who live together through choice. Therefore it includes housemates and cohabitees but not those in care homes, prisons, residential or inpatient psychiatric settings.

The UK government definition does not include those under the age of 16 within their cross-governmental definition of domestic violence, but we chose to include children and adolescents in our approach, in part because it is not always possible to determine the age of the patient from the extract but also because we felt it was not appropriate to exclude them.

The rationale for identifying domestic violence specifically is that the interventions are distinct from those designed for community violence (violence perpetrated by strangers or acquaintances) (Krug, 2002).

Our NLP definition of sexual violence includes any unwanted sexual act, unwanted sexual comments or advances, unwanted attempts to obtain a sexual act and acts to traffic. It includes rape, sexual harrassment, sexual assault, forced marriage, stalking and reproductive coercion and control.

Forms of sexual violence that fulfil the definition for physical violence, such as rape and sexual assault, were recorded as both physical and sexual violence.

A challenge with defining sexual violence in psychiatric EHRs are the symptoms of sexual disinhibition and sexually inappropriate behaviour. Whilst the approach does not consider intentionality necessary for the affirmation of a violent act, these acts and behaviours were

For peer review only

not considered sexual violence unless they were directed towards another person (as opposed to generalised) or they explicitly caused harm to another person

For peer review only

BMJ Open

Can natural language processing models extract and classify instances of interpersonal violence in mental healthcare electronic records: an applied evaluative study

Journal:	*BMJ Open*
Manuscript ID	bmjopen-2021-052911.R2
Article Type:	Original research
Date Submitted by the Author:	29-Nov-2021
Complete List of Authors:	Botelle, Riley; Guy's King's and Saint Thomas' School of Medicine<br>Bhavsar, Vishal; King's College London, Section of Women's Mental Health, Department of Health Services and Population Research<br>Kadra-Scalzo, Giouliana; King's College London, Psychological Medicine, Institute of Psychiatry, Psychology and Neuroscience<br>Mascio, Aurelie; King's College London, Psychological Medicine, Institute of Psychiatry, Psychology and Neuroscience<br>Williams, Marcus; Guy's King's and Saint Thomas' School of Medicine<br>Roberts, Angus; King's College London, Biostatistics and Health Informatics; Health Data Research UK<br>Velupillai, Sumithra; King's College London, Psychological Medicine, Institute of Psychiatry, Psychology and Neuroscience<br>Stewart, Robert; King's College London, Psychological Medicine, Institute of Psychiatry, Psychology and Neuroscience; South London and Maudsley Mental Health NHS Trust
**Primary Subject Heading**:	Health informatics
Secondary Subject Heading:	Mental health, Public health, Research methods
Keywords:	Health informatics < BIOTECHNOLOGY & BIOINFORMATICS, MENTAL HEALTH, PSYCHIATRY, PUBLIC HEALTH

SCHOLARONE™
Manuscripts

I, the Submitting Author has the right to grant and does grant on behalf of all authors of the Work (as defined in the below author licence), an exclusive licence and/or a non-exclusive licence for contributions from authors who are: i) UK Crown employees; ii) where BMJ has agreed a CC-BY licence shall apply, and/or iii) in accordance with the terms applicable for US Federal Government officers or employees acting as part of their official duties; on a worldwide, perpetual, irrevocable, royalty-free basis to BMJ Publishing Group Ltd ("BMJ") its licensees and where the relevant Journal is co-owned by BMJ to the co-owners of the Journal, to publish the Work in this journal and any other BMJ products and to exploit all rights, as set out in our licence.

The Submitting Author accepts and understands that any supply made under these terms is made by BMJ to the Submitting Author unless you are acting as an employee on behalf of your employer or a postgraduate student of an affiliated institution which is paying any applicable article publishing charge ("APC") for Open Access articles. Where the Submitting Author wishes to make the Work available on an Open Access basis (and intends to pay the relevant APC), the terms of reuse of such Open Access shall be governed by a Creative Commons licence – details of these licences and which Creative Commons licence will apply to this Work are set out in our licence referred to above.

Other than as permitted in any relevant BMJ Author's Self Archiving Policies, I confirm this Work has not been accepted for publication elsewhere, is not being considered for publication elsewhere and does not duplicate material already published. I confirm all authors consent to publication of this Work and authorise the granting of this licence.

For peer review only

Can natural language processing models extract and classify instances of interpersonal violence in mental healthcare electronic records: an applied evaluative study

Riley Botelle (1), Vishal Bhavsar (2), Giouliana Kadra-Scalzo (3), Aurelie Mascio (3), Marcus Williams (1), Angus Roberts (4,5), Sumithra Velupillai (3) and Robert Stewart (3,6)

1. GKT School of Medical Education, King's College London
2. Section of Women's Mental Health, Department of Health Services and Population Research, King's College London.
3. Psychological Medicine, Institute of Psychiatry, Psychology and Neuroscience, King's College London
4. Biostatistics and Health Informatics, King's College London
5. Health Data Research UK
6. South London and Maudsley NHS Foundation Trust, London

Corresponding author's details: riley.botelle@gmail.com

Funding: This project received no specific funding. The Clinical Record Interactive Search (CRIS) system was funded and developed by the National Institute for Health Research (NIHR) Biomedical Research Centre at South London and Maudsley NHS Foundation Trust and King's College London and by a joint infrastructure grant from Guy's and St Thomas' Charity and the Maudsley Charity (grant number BRC-2011-10035). RS, SV, AR and GKS receive salary support from the National Institute for Health Research (NIHR) Biomedical Research Centre at South London and Maudsley NHS Foundation Trust and King's College London. RS is a NIHR Senior Investigator. GKS has received support from the Early Career Research Award, funded by the NIHR Maudsley BRC. VB receives salary support from King's College London via a secondment to Lambeth Council. AR receives support from Health Data Research UK, an initiative funded by UK Research and Innovation, Department of Health and Social Care (England) and the devolved administrations, and leading medical research charities. The views expressed are those of the author(s) and not necessarily those of the NHS, the NIHR or the Department of Health. Funders had no role in the study design; in the collection, analysis and interpretation of the data; in the writing of the report; and in the decision to submit the paper for publication.

Competing Interests: RS has received research support in the last 36 months from Janssen, Takeda and GSK. GKS has received funding from Janssen and Lundbeck. SV has received funding from Janssen. AM has received funding from Takeda California, Inc.

Data Availability: The CRIS database has received ethical approval for secondary analysis: Oxford REC C, reference 18/SC/0372. On request, and after appropriate arrangements, the data and modelling employed in this study can be viewed within the secure system firewall.

Word count: 3769 words, 2 figures, 7 tables

For peer review only

Abstract

Objective: This paper evaluates the application of a natural language processing (NLP) model for extracting clinical text referring to interpersonal violence using electronic health records (EHRs) from a large mental healthcare provider.

Design: A multidisciplinary team iteratively developed guidelines for annotating clinical text referring to violence. Keywords were used to generate a dataset which was annotated (i.e. classified as affirmed, negated or irrelevant) for: presence of violence, patient status (i.e. as perpetrator, witness and/or victim of violence) and violence type (domestic, physical and/or sexual). An NLP approach using a pre-trained transformer model (BioBERT) was fine-tuned on the annotated dataset and evaluated using 10-fold cross-validation.

Setting: We used the Clinical Records Interactive Search (CRIS) database, comprising over 500,000 de-identified EHRs of patients within the South London and Maudsley NHS Foundation Trust, a specialist mental healthcare provider serving an urban catchment area.

Participants: Searches of CRIS were carried out based on 17 pre-defined keywords. Randomly selected text fragments were taken from the results for each keyword, amounting to 3771 text fragments from the records of 2832 patients.

Outcome measures: We estimated precision, recall and F1 score for each NLP model. We examined sociodemographic and clinical variables in patients giving rise to the text data, and frequencies for each annotated violence characteristic.

Results: Binary classification models were developed for six labels (violence presence, perpetrator, victim, domestic, physical and sexual). Among annotations affirmed for the presence of any violence, 78% (1724) referred to physical violence, 61% (1350) referred to patients as perpetrator, and 33% (731) to domestic violence. NLP models' precision ranged from 89% (perpetrator) to 98% (sexual); recall ranged from 89% (victim, perpetrator) to 97% (sexual).

Conclusions: State of the art NLP models can extract and classify clinical text on violence from EHRs at acceptable levels of scale, efficiency and accuracy.

Key terms: natural language processing, NLP, violence, victimisation, perpetrator, electronic health records

Strengths and limitations of this study:

- Previous NLP models for extracting violence from mental health EHRs have focused on single forms of violence, rather than capturing violence more broadly as this methodology does.
- This study fills a gap where newer fine-tuned transformer-based NLP models such as Bio-BERT have not yet been extensively researched in mental health applications.
- The methodology used can estimate the occurrence of clinical references to violence in EHRs but it cannot be used to estimate the prevalence of violent events without further assumptions.

For peer review only

Introduction

Interpersonal violence, defined as the intentional use of physical force or power, threatened or actual, against another person [1], causes significant mental and physical morbidity [2–4]. Interpersonal violence may further be distinguished as domestic, physical and sexual violence. By its definition, interpersonal violence involves one or more perpetrator(s), one or more victim(s), and may also involve witnesses.

People with mental illness are more likely to experience violent victimisation compared to the general population [5]. For example, women with pre-existing mental illness are significantly more likely to experience victimization compared to the general population with 15–45% of patients reporting experiences of victimization in the past year, and 40–90% reporting lifetime victimization [5,6]. Domestic violence victimisation is also more frequently reported by people with mental illness, with 27% of women and 13% of men with severe mental illnesses (SMI) reporting experiences of domestic violence in the past year, compared to 9% and 5% respectively in general population samples [6]. Individuals with established mental disorders also experience greater occurrence of community and sexual violence compared to the general population [7]. Evidence also suggests associations between a diagnosis of SMI and higher perpetration of violence, compared to the general population [8]. Literature examining witnessing of violence is sparse, nevertheless there are some evidence indicating greater rates of witnessed violence among people with mental illness, and a deterimental impact of witnessed violence on mental health [9,10]. Adults who have experienced victimization, and more specifically physical or sexual assault are at greater risk of mental disorders including PTSD, depression, and psychosis [11,12]. The impact of violence is mutlifold with significant economic, service and personal costs. For example, within mental health settings, violence occurs most frequently on inpatient psychiatric units, with an estimated cost of £20.5 million per year [13].

Because of the consistent correlation between violence and psychiatric morbidity, mental health services are important settings for understanding and improving societal responses to violence. In particular, a large proportion of individuals in contact with mental health services have a history of violence exposure, including through victimisation, perpetration, and/or witnessing interpersonal violence. Despite this, health services' data on interpersonal violence (including the role of patient as perpetrator, victim, or witness, whether violence being referred to is domestic, physical and/or sexual) are inconsistent. Not all forms of violence are enquired about routinely by professionals, and some forms of violence are not routinely assigned diagnostic codes [14] and so are not easily identifiable in electronic records.

Electronic health records kept by mental health services offer a valuable resource to understand how and why interpersonal violence occurs in this population and examine how

For peer review only

services respond to violence presentation (both as a victim and perpetrator) in relation to treatment and support. Improved understanding of interpersonal violence experienced by people using mental health services, and the response of professionals to violence, could improve care quality and patient safety. Mental health services can collect and record data on interpersonal violence, but structured data (e.g. on violent incidents) are predominantly collected on individuals in inpatient settings, and not all forms of violence experienced and reported by patients may be recorded in this way [15]. We have previously employed text-processing rules to extract violence information from unstructured clinical text from electronic health records, with a focus on physical violence [16].

Natural language processing (NLP) methods offer a flexible automated approach to extracting text data from large bodies of unstructured text [17,18]. NLP models have been developed for the extraction of information on diagnosis, symptoms, and treatment from clinical text [15–17, 19]. Early NLP investigations using systems such as MedLEE relied on pattern matching and logical rules [20]. Developments in machine learning led to further advances and broad coverage applications, such as cTAKES [21] and CLAMP [22]. In mental health research, NLP models have been developed to extract constructs such as phenotype mentions [23], and symptoms of severe mental illness [22]. Recent advances in deep neural network algorithms for NLP modelling, and particularly in transformer-based language models such as BERT [24] have shown promising results, also in the biomedical domain with, for example, the BioBERT model [25], as shown in comparative analyses [26,27]. This research indicated that using a BioBERT fine-tuned algorithm outperformed most other algorithms. However, while these state-of-the-art approaches generate greater accuracy, they have not yet been extensively researched in mental health services. This paper does not evaluate or compare models but instead builds on these previous comparative analyses to evaluate the application of an existing model (BioBERT) onto a real-world problem.

Previous NLP approaches to capturing violence in mental health clinical text have had significant limitations such as focusing on single forms forms of violence i.e. physical assault victimisation [28], rather than capturing a broad range of possibly co-existing violence characteristics (e.g. perpetration, victimisation, witnessing), forms of harm (physical, sexual), and the nature of the relationship between victim and perpetrator (domestic, community) in the same NLP approach. Additionally, previous literature has utilised limited data e.g. only inpatient data. Unstructured data is challenging to examine due to complexity and volume, as evidenced by paucity of usage in previous studies.

Objective

For peer review only

To develop and evaluate natural language processing models for the extraction and classification of references to interpersonal violence from clinical text drawn from EHRs at a large mental health provider.

Methods

Data source

Data were drawn from the Clinical Record Interactive Search (CRIS), a database of de-identified EHRs from the South London and Maudsley (SLaM) NHS Foundation Trust. SLaM provides specialist mental health services to around 1.3 million residents of four boroughs in South-East London (Lambeth, Southwark, Lewisham and Croydon). The CRIS database was developed in 2008 and allows researchers to access structured data (such as demographic data from forms) and unstructured data (such as free text entered by clinicians based on clinical encounters) for mental health research [28–31]. Currently there are over 500,000 patient records represented in CRIS, spanning 2007-present. CRIS applies a robust data security and governance model for data access and has been approved as a database for secondary analysis (Oxford Research Ethics Committee, reference 18/SC/0372).

Patient and Public Involvement statement

The CRIS database was developed with extensive service user involvement and adheres to strict governance frameworks managed by service users. Data are used in an entirely de-identified and secure format and all patients have the choice to opt-out of their de-identified data being used [32]. Each research project is reviewed by a service-user led oversight committee of the National Institute of Health Research Biomedical Research Centre.

Methodological framework for annotation

Figure 1: Process of annotation, development and evaluation of NLP models

Figure 1 summarises the annotation process. To generate annotation data for NLP model development we generated a list of violence-related keywords based on the literature, clinical experience, and informatics expertise (Appendix 1). An embedding model (Word2Vec trained on all CRIS records) was used to generate additional synonyms that were subsequently reviewed and included in the list of keywords. Furthermore, the embedding model was used to capture misspellings for each keyword.

For each keyword (17 in total; 7 nouns and 10 verbs), CRIS text containing that keyword was randomly sampled, extracting fragments containing the keyword and 300 text characters either side. We aimed to select 300 text fragments for each noun keyword, and 200 for each verb keyword. These text fragments were then annotated into labels for

For peer review only

reference to violence by two clinical medical students (RB and MW). Initial label definitions, results and queries were reviewed and discussed by a multidisciplinary team which met weekly to formulate a set of further labels for NLP development based on the violence and mental health literature. Final labels are described further below. Weekly meetings also adjudicated on disagreements between annotators. We developed annotation guidelines (Appendix 2) which were iteratively developed based on discussion and queries raised by annotation. Discussions and rationale are detailed in Appendix 3. Inter-annotator agreement was estimated on a subset, using % agreement and Cohen's kappa.

<u>Labels used for annotating text fragments</u>

Annotations were carried out for seven labels which were developed based on the WHO definition of interpersonal violence [1]. For each text label described below and exemplified in Table 1, annotations classified text fragments as follows:

1. *Violence presence*: we annotated for the presence of any reference to **violence** in the text fragment, classifying fragments into: affirmed (where the characteristic was present), negated (characteristic absent), or irrelevant (where the keyword was employed in the text to refer to a context other than violence).
2. *Patient status*: we assigned three non-exclusive labels for the status of the patient within the text fragment, classified into whether the patient was perpetrator, victim, and/or witness, to the text fragments where violence presence was annotated as affirmed. The label **perpetrator** was affirmed where the patient was referred to as the person using physical force or power, and the label **victim** was affirmed where the fragment referred to the patient as the person violence was used against. The label **witness** was affirmed where the fragment referred to the patient as having observed violence through seeing or hearing violence occurring and this was the primary description, i.e. they were not a victim or perpetrator of that violence.
3. *Violence type*: we also assigned labels classifying violence by forms of harm (physical, sexual), and based on the relationship between the victim and perpetrator (domestic). The label **physical** was affirmed where the text fragment referred to violence which used physical force, or resulted in or had a high likelihood of resulting in physical injury. The label **sexual** was affirmed where the text fragment referring to violence included unwanted sexual acts, unwanted sexual comments or advances, or unwanted attempts to obtain a sexual act. This included references to rape, sexual harrassment, sexual assault, forced marriage, stalking and reproductive coercion and control. The

For peer review only - http://bmjopen.bmj.com/site/about/guidelines.xhtml

label **domestic** was affirmed where text referred to violence between family members, intimate partners, ex-intimate partners and household members. As with *patient status*, these labels were also non-exclusive, so that text fragments could include any combination of affirmed labels listed above.

Table 1: Examples of text fragments, with keywords italicised, extracted for annotation in this study, alongside corresponding labels and assigned annotations.

Example of text fragment	Label	Annotation
"They were *abused* in their childhood"	Violence presence, victim	Affirmed
"Patient used to *hit* her partner"	Violence presence, perpetrator; physical, domestic	Affirmed
"Patient stabbed his roommate"	Violence presence, perpetrator; physical, domestic	Affirmed
"Expressed a lot of interest in *violence*, nazism"	Violence presence	Irrelevant
"No *violence* or aggression noted"	Violence presence	Negated

The labels listed above were annotated in the following steps. First, each text extract was annotated for the violence presence label, classifying the fragment of text as affirmed, negated or irrelevant. If this label was affirmed, the fragment was further annotated with labels for patient status (victim, perpetrator, and/or witness) and violence type (domestic, physical, sexual). This is represented in Figure 2.

Figure 2: Flowchart of extract annotation process

NLP model development

For the development of NLP models, we used the pre-trained BioBERT model [25] and fine-tuned it on the annotated dataset. Each set was generated independently (ensuring no overlap). Three datasets were used for: model testing and training (development stage, 3771 sentences) model fine-tuning (1411 sentences), and model blind testing (100 sentences). We aimed to produce seven binary classification models for each annotated label. We evaluated the models with 10-fold cross validation, comprising 10% annotated text extracts for testing, and 90% text extracts in training, in each fold. We estimated standard markers of NLP performance: precision (or positive predictive value), recall (or sensitivity) and F1 score (the harmonic mean of precision and recall), using weighted averages to take

For peer review only

into account the dataset's imbalance (i.e. differing numbers of extracts generated for each keyword). Reported scores corresponded to the mean across the 10 test sets. The model fine-tuning test set was run on 1411 sentences extracted randomly from the CRIS database (sentences not used to train the model or test the development). This set was randomly sampled using the clinical records not previously used for training, and the same list of 27 keywords. This set was then manually annotated following the same guidelines as the training set and compared against the output generated by the NLP model. Weighted loss functions (cross entropy loss with custom weight parameters) were used to account for unbalanced datasets. The blind model testing set of 100 sentences were generated to review sample generation, annotation and model assessment.

For descriptive purposes we examined sociodemographic and diagnostic characteristics of patients whose records gave rise to the text extracts used for NLP development, and also assessed two way overlap of affirmed non-exclusive annotation labels.

Results

Sampling of text for NLP development resulted in 14 of the 17 keywords being sampled as planned. Three keywords generated a smaller number of selected fragments: 'rape' 188; 'fought' 124 and 'strangul' 59, resulting in a final annotation dataset of 3771 text extracts. Fine-tuning of the pre-trained BioBERT model using the annotation dataset resulted in six binary classification models - for one annotation label (witness) we were unable to generate a model due to insufficient data size (n=53). The performance of each of the six NLP models is reported in Table 2. Two types of testing were carried out to evaluate the model's suitability. A 10 fold cross validation was conducted on the training and testing dataset (comprising 3771 text extracts), for which the average performance on the test sets is reported. Precision ranged from 89% (for the perpetrator label) to 98% (sexual) and recall ranged from 89% (victim, perpetrator) to 97% (sexual). Inter-annotator agreement was high: 82-96% (60-85% Cohen's kappa) for the six annotation labels. Additionally, a separate blind test consisting of 100 newly annotated sentences not used for model training or fine-tuning was assessed. For this blind test we used a confidence level of 90% (meaning only sentences that the model classified with 90% or above confidence were included, in order to eliminate "confusing statements", which made up 1% of the dataset). This 90% threshold was then used for application deployment.

Table 2: NLP model performances on the training and testing dataset (3,771 text extracts) and well as a blind test set with a 90% probability threshold (100 sentences) for the six labels.

For peer review only

	Training set (average score on 10-fold cross-validation)			Blind test set
**Annotation label**	**Precision**	**Recall**	**F1-score**	**F1-score**
**Violence presence**	93%	93%	93%	95%
**Patient status: Perpetrator**	89%	89%	89%	85%
**Patient status: Victim**	91%	89%	91%	90%
**Violence type: Domestic**	94%	94%	94%	93%
**Violence type: Physical**	91%	92%	91%	98%
**Violence type: Sexual**	98%	97%	97%	93%

Table 3: Proportion of each label in the training and testing dataset – affirmed or negated/irrelevant. Each text extract was first annotated for the violence presence label, then if this was affirmed, further annotated for the other labels related to patient status and violence type (see figure 1 for further details). Therefore denominator totals for the violence presence label is larger than that for the other labels.

**Annotation label**	**Affirmed, N (%)**	**Negated or irrelevant, N (%)**	**Total**
**Violence presence**	2199 (58)	1572 (42)	3771
**Patient status: Perpetrator**	1350 (61)	849 (39)	2199
**Patient status: Victim**	731 (33)	1468 (67)	2199
**Violence type: Domestic**	723 (33)	1476 (67)	2199
**Violence type: Physical**	1724 (78)	475 (22)	2199
**Violence type: Sexual**	353 (16)	1846 (84)	2199

Table 3 shows the breakdown of annotation labels across the training and testing dataset at an annotation level. Overall, 58% (2,199) of the text extracts were affirmed for violence presence. Of these, the proportion of affirmed examples for patient status ranged from 33% (victim) to 61% (perpetrator), and for violence type from 16% (sexual) to 78% (physical). Some affirmed labels overlapped, because a fragment of text could have contained multiple references to different types of violence (e.g. both physical and sexual violence) and different patient statuses (e.g. as both perpetrator and victim). Table 4 describes these overlaps. For instance, the number of examples where *perpetrator* was affirmed (n=1350) overlapped with affirmed for *physical* violence in almost 80% of the examples (n=1078), while the overlap with *victim* was rarer (8%, n=113).

For peer review only

Table 4: Overlap of labels present in affirmed annotations, showing the number and percentage of annotations that shared different labels.

	Perpetrator, N (%)	Victim, N (%)	Sexual, N (%)	Physical, N (%)
**Perpetrator**	-	-	-	-
**Victim**	113 (8.4)	-	-	-
**Sexual**	150 (11.1)	199 (27.2)	-	-
**Physical**	1078 (79.9)	616 (84.3)	304 (86.1)	-
**Domestic**	331 (24.5)	318 (43.5)	104 (29.5)	593 (34.4)
Column total	*1350 (100.0)*	*731 (100.0)*	*353 (100.0)*	*1724 (100.0)*

Table 5 reports model-to-annotator agreement, that is, agreement between the two annotators combined and the model. Table 6 reports errors made by the model. Total errors represent instances where the model predicted differently to the annotators, whilst "false positives" represent instances where the model classified an instance as violent whilst annotators classified it as irrelevant or negated. "False negatives" are instances that the model classified as irrelevant or negated whilst annotators markers as affirmed. Both tables were computed using the model training and testing set of 3771 sentences and indicate high agreement between the models and annotators.

Table 5: Kappa agreement between manually and automatically assigned categories in the training and testing set (3771 sentences)

Annotation label	Model-to-annotator agreement
**Violence presence**	98.1%
**Patient status: Perpetrator**	97.4%
**Patient status: Victim**	96.2%
**Violence type: Domestic**	98.7%
**Violence type: Physical**	98.3%
**Violence type: Sexual**	96.8%

For peer review only

Table 6: Model error analysis on training and testing set (3771 sentences)

Annotation label	False positives	False negatives	Total number of errors
**Violence presence**	24	10	34
**Patient status: Perpetrator**	35	12	47
**Patient status: Victim**	5	40	45
**Violence type: Domestic**	10	5	15
**Violence type: Physical**	7	27	34
**Violence type: Sexual**	11	10	21

Demographic features of the patients included in the training and testing dataset sample are presented in Table 7. The sample represented in total 2,832 patients, of whom 57% were female, 45% were aged 40-60 at the time the fragment was extracted, 66% were single, 52% were of White ethnic background, and 36% were diagnosed with psychotic disorders (ICD-10 codes F20-29) [33]. This NLP approach captures references to violence occurring in a large body of patients presenting to a mental health service who are not necessarily presenting for violence-related reasons.

Table 7: Characteristics of patients whose text extracts were annotated as part of this study.

	Frequency, N (%)
**Age**	
**<20**	167 (5.9)
**20<40**	791 (27.9)
**40<60**	1273 (45.0)
**60<80**	458 (16.2)
**80<**	141 (5.0)
**Missing**	2 (0.1)
**Gender**	
**Male**	1216 (42.9)

For peer review only

**Female**	1614 (57.0)
**Missing**	2 (0.1)
**Marital status**	
**Single**	1865 (65.9)
**Married/cohabiting**	344 (12.2)
**Divorced/separated**	262 (9.3)
**Widowed**	85 (3.0)
**Missing**	276 (9.8)
**Ethnicity**	
**White**	1482 (52.3)
**Black**	104 (3.7)
**Asian**	160 (5.7)
**Mixed**	885 (31.3)
**Other**	90 (3.2)
**Missing**	111 (3.9)
**ICD-10 Diagnosis**	
**F0-9: Organic, including symptomatic, mental disorders**	185 (6.5)
**F10-19: Mental and behavioural disorders due to psychoactive substance use**	94 (3.3)
**F20-29: Schizophrenia, schizotypal and delusional disorders**	1031 (36.4)
**F30-39: Mood [affective] disorders**	451 (15.9)
**F40-49: Neurotic, stress-related and somatoform disorders**	203 (7.2)
**F50-59: Behavioural syndromes associated with physiological disturbances and physical factors**	18 (0.6)
**F60-69: Disorders of adult**	236 (8.3)

For peer review only

**personality and behaviour**	
**F70-79: Mental retardation**	53 (1.9)
**F80-89: Disorders of psychological development**	98 (3.5)
**F90-99: Behavioural and emotional disorders with onset usually occurring in childhood and adolescence and unspecified mental disorder**	211 (7.6)
**No axis 1 diagnosis**	25 (0.9)
**G: Diseases of the nervous system, X: Intentional self-harm, assault or Z: Factors influencing health status and contact with health services***	163 (5.8)
**Missing**	64 (2.3)
**Total**	**2832** (100.0)

*ICD-10 categories G and X were combined with ICD-10 category Z due to small numbers of participants (n<10) in these categories, in order to limit identification of participants.

Discussion

To our knowledge, this is the first study that has used a NLP approach to code free-text data from a large and diverse source of electronic mental health records to ascertain violence according to presence, agent and type. There is limited previous research examining NLP to extract violence-related information in EHRs but includes utilising text rules to ascertain violent behaviour as antecedents to supervised confinement [16] and the employment of a bag of words machine learning approach to extract information on physical assault victimisation in CRIS data [28]. Our approach captures a much broader range of experiences. We successfully developed an annotated dataset of clinical text references to interpersonal violence, using a multidisciplinary clinical academic group. We used this dataset to develop binary classification NLP models for extracting and classifying clinical text fragments referring to interpersonal violence in mental health EHRs, including for patient status (perpetrator, victim) and violence type (domestic, physical, sexual). Models were developed with a state-of-the-art NLP algorithm (fine-tuned BioBERT) and displayed very

For peer review only

good performance based on accepted evaluation criteria. A planned NLP model for extracting references to witnessed violence was not successfully developed due to an insufficient sample size.

This study had several limitations that need to be borne in mind when interpreting the findings. Although agreement between annotators was generally good, some disagreements occurred. Examples of disagreements included ascertaining the status of the patient as perpetrator/victim/witness in a text fragment and whether “fighting” was considered interpersonal violence or a colloquial term for verbal arguments. As our focus was on interpersonal violence, we did not annotate or develop models for individuals forced to fight in armed conflict, for example. Keywords used in this study were selected to capture as many instances of interpersonal violence in clinical text as possible but may not have captured all violence categories of “hidden violence”. For instance, keywords related to female genital mutilation, forced marriage, trafficking, neglect, sensory deprivation, harrassment, stalking, or reproductive coercion and control were not included, and would need separate analysis for NLP development. These specific forms of violence could, however, be readily addressed using an “add-on” to the approach presented here. Similarly, emotional and psychological violence were not included in the list of keywords, and capturing these characteristics through NLP is likely to be more complex because of the broader way in which this is likely to be described in clinical text. Annotation was carried out on the basis of the meaning and sense of the text fragments sample in this study, rather than entire EHR documents. It is possible that annotations based on entire documents would have delivered slightly different results, but this would have been challenging to implement given the quantity of documents that would be needed to be manually labeled in order to capture enough examples. This model could potentially be improved by additional fine-tuning on a clinical dataset. Lastly, we present an NLP approach to extracting clinical text fragments which refer to violence from mental health records. Given the sufficiently accurate performance reported in this study, this approach can extract references to violence where it is written down in clinical records, but is restricted to these recorded instances, and cannot be considered a method for measuring prevalence of all experienced violence (without further assumptions which are likely to depend on the situation). This is likely to continue to require asking patients themselves, or linking data from other sources such as hospital data or crime records. Indeed, these approaches could be helpful in understanding the processes by which violence is identified by clinicians and recorded in clinical notes.

This study had some specific advantages. Annotations were derived from rich and diverse free-text data from service-users’ clinical notes, as opposed to structured data. The data are derived from progress notes entered by wide array of clinical groups and

For peer review only

professionals, therefore increasing the chances of detecting violence information. Furthermore, given the longitudinal nature of our dataset, information was recorded over a sixteen-year period (2007-2019), which further increases our ability to detect experiences of violence, if recorded in the clinical notes. All annotations were coded using human annotators, with high inter-annotator agreement, which support the robustness of our approach. Lastly, we used a state-of-the-art NLP pre-trained transformer model, BioBERT, which has been shown to outperform methods more traditionally used for symptoms detection such as support vector machine [26] and this allowed us to develop fine-tuned models with very promising results. As this study did not make comparative evaluations, it is possible that a simpler baseline model could also provide a similar level of performance, whilst requiring fewer resources. The BioBERT model is readily deployable and interpretable, with all scripts made publicly available on GitHub. The fine-tuned model is relatively light (450Mb) and easy to deploy in clinical settings. The model was run in this study utilising Graphics Processing Unit (GPU)-accelerated analytics, which may limit replicability.

NLP offers a reliable, automated, scalable approach to extracting summary information from EHRs. Reasonably accurate methods for extracting clinical text referring to interpersonal violence can support further research on correlates of clinically-identified violence, and how professionals and services respond to violence. Evaluation of NLP-derived violence indicators in relation to linked external data on hospital admissions, GP registers, and crimes data could allow some assessment of the reliability with which clinicians record violence when it occurs. Based on the inter-operability of NLP algorithms across different EHR formats, models developed in this study could be feasibly applied to mental health EHRs in other sites (including CRIS databases elsewhere in the UK) and other free-text containing EHRs. The main limitation to porting this model to other sites would be differences in language that may lead to a slightly different list of keywords. However, the current list already captures many common terms related to violence and was reviewed by clinicians that work in several NHS Trusts to ensure generalisability. Furthermore, we have developed an NLP tool that is easily adaptable and allows quick fine-tuning and deployment of the model if needed.

There is also a significant clinical need for methods which can accurately summarise relevant historic information for clinicians to review and use in decision making at the point of care. This is particularly relevant in the arena of violence reduction and mental health, where serious incident reviews and charitable organizations have called for renewed attention to the need for accurate summaries of previous violence, and improved information sharing with other agencies [34]. By summarising previous exposure to violence, clinical information systems might improve the efficiency of clinical encounters, e.g. by reducing clinical time

For peer review only - http://bmjopen.bmj.com/site/about/guidelines.xhtml

taken up with collecting information on previous violence. Future research might also consider the clinical benefits of accurate summaries of previous interpersonal violence which might be reported to treating clinicians in real time, to aid decision making. We suggest that attention is warranted into the ethical and regulatory challenges of translating NLP methods for violence into practice.

We were unable to model witnessing violence using NLP in this study. This could form the focus of future research based on the possible impact of witnessing violence on health [35]. The infrequency of witnessed violence during the annotation process may reflect that clinicians are not frequently enquiring about these experiences, or they are not being recorded.

We did not consider temporality of violence mentions, but this is something that we hope to integrate in future to improve the usability of the models.

Conclusion

We have demonstrated that it is possible to use state-of-the-art NLP methods to extract clinical text referring to violence (including distinguishing patients as perpetrators and victims, as well as violence types such as physical, domestic, and sexual) at scale with acceptable accuracy in mental health EHRs. This could support further research into the pathways by which violence is identified in clinical practice, and the effectiveness of systems for identifying, assessing and managing interpersonal violence. However, while NLP approaches might offer a sufficiently accurate method for summarising violence-related information to aid clinical decision making, considerable ethical and regulatory questions remain.

Author contributions:

RB drafted the first version of the manuscript. RB and MW annotated the dataset. AM developed the NLP models. VB, GKS, RS provided clinical and epidemiological input in relation to the motivation for the work, annotation guideline development and definitions. SV and AR provided technical input and guidance in relation to the NLP model development. All authors drafted and/or critically revised the manuscript and approved the final version of the manuscript.

Ethics Statement:

The CRIS database has received ethical approval for secondary analysis: Oxford REC C, reference 18/SC/0372. The CRIS database was developed with extensive service user involvement and adheres to strict governance frameworks managed by service users. Data are used in an entirely de-identified and secure format and all patients have the choice to opt-out of their de-identified data being used.

For peer review only

References

1 Krug EG, Mercy JA, Dahlberg LL, *et al.* The world report on violence and health. *The Lancet* 2002;**360**:1083–8. doi:10.1016/S0140-6736(02)11133-0

2 Reza A, Mercy JA, Krug E. Epidemiology of violent deaths in the world. *Injury Prevention* 2001;**7**:104–11. doi:10.1136/ip.7.2.104

3 Olofsson N, Lindqvist K, Shaw BA, *et al.* Long-term health consequences of violence exposure in adolescence: a 26–year prospective study. *BMC Public Health* 2012;**12**:411. doi:10.1186/1471-2458-12-411

4 López-Martínez AE, Serrano-Ibáñez ER, Ruiz-Párraga GT, *et al.* Physical Health Consequences of Interpersonal Trauma: A Systematic Review of the Role of Psychological Variables. *Trauma, Violence, & Abuse* 2018;**19**:305–22. doi:10.1177/1524838016659488

5 Maniglio R. Severe mental illness and criminal victimization: a systematic review. *Acta Psychiatrica Scandinavica* 2009;**119**:180–91. doi:https://doi.org/10.1111/j.1600-0447.2008.01300.x

6 Khalifeh H, Moran P, Borschmann R, *et al.* Domestic and sexual violence against patients with severe mental illness. *Psychol Med* 2015;**45**:875–86. doi:10.1017/S0033291714001962

7 Khalifeh H, Johnson S, Howard LM, *et al.* Violent and non-violent crime against adults with severe mental illness. *British Journal of Psychiatry* 2015;**206**:275–82. doi:10.1192/bjp.bp.114.147843

8 Fazel S, Gulati G, Linsell L, *et al.* Schizophrenia and violence: Systematic review and meta-analysis. *PLoS Medicine* 2009;**6**. doi:10.1371/journal.pmed.1000120

9 Kadra G, Dean K, Hotopf M, *et al.* Investigating Exposure to Violence and Mental Health in a Diverse Urban Community Sample: Data from the South East London Community Health (SELCoH) Survey. *PLOS ONE* 2014;**9**:e93660. doi:10.1371/journal.pone.0093660

10 Bhavsar V, Dean K, Hatch SL, *et al.* Psychiatric symptoms and risk of victimisation: a population-based study from Southeast London. *Epidemiology and Psychiatric Sciences* 2019;**28**:168–78. doi:10.1017/S2045796018000537

11 Resnick HS, Acierno R, Kilpatrick DG. Health impact of interpersonal violence. 2: Medical and mental health outcomes. *Behav Med* 1997;**23**:65–78. doi:10.1080/08964289709596730

12 Raune D, Bebbington P, Dunn G, *et al.* Event attributes and the content of psychotic experiences in first-episode psychosis. *PSYCHOL MED* 2006;**36**:221–30.

13 Flood C, Bowers L, Parkin D. Estimating the costs of conflict and containment on adult acute inpatient psychiatric wards. *Nurs Econ* 2008;**26**:325–30, 324.

14 Walby S. Improving the statistics on violence against women. *Statistical Journal of the United Nations ECE* 2005;**22**:193–216. doi:10.3233/SJU-2005-223-402

15 Robson D, Spaducci G, McNeill A, *et al.* Effect of implementation of a smoke-free policy on physical violence in a psychiatric inpatient setting: an interrupted time series analysis. *The Lancet Psychiatry* 2017;**4**:540–6. doi:10.1016/S2215-0366(17)30209-2

16 Cullen AE, Bowers L, Khondoker M, *et al.* Factors associated with use of psychiatric intensive care and seclusion in adult inpatient mental health services. *Epidemiology and Psychiatric Sciences* 2018;**27**:51–61. doi:10.1017/S2045796016000731

17 Ford E, Carroll JA, Smith HE, *et al.* Extracting information from the text of electronic medical records to improve case detection: a systematic review. *Journal of the American Medical Informatics Association* 2016;**23**:1007–15. doi:10.1093/jamia/ocv180

For peer review only

18 Kreimeyer K, Foster M, Pandey A, *et al.* Natural language processing systems for capturing and standardizing unstructured clinical information: A systematic review. *Journal of Biomedical Informatics* 2017;**73**:14–29. doi:10.1016/j.jbi.2017.07.012
19 Wang Y, Wang L, Rastegar-Mojarad M, *et al.* Clinical information extraction applications: A literature review. *Journal of Biomedical Informatics* 2018;**77**:34–49. doi:10.1016/j.jbi.2017.11.011
20 Friedman C, Alderson PO, Austin JH, *et al.* A general natural-language text processor for clinical radiology. *J Am Med Inform Assoc* 1994;**1**:161–74.
21 Savova GK, Masanz JJ, Ogren PV, *et al.* Mayo clinical Text Analysis and Knowledge Extraction System (cTAKES): architecture, component evaluation and applications. *J Am Med Inform Assoc* 2010;**17**:507–13. doi:10.1136/jamia.2009.001560
22 Soysal E, Wang J, Jiang M, *et al.* CLAMP – a toolkit for efficiently building customized clinical natural language processing pipelines. *J Am Med Inform Assoc* 2017;**25**:331–6. doi:10.1093/jamia/ocx132
23 Wu H, Hodgson K, Dyson S, *et al.* Efficient Reuse of Natural Language Processing Models for Phenotype-Mention Identification in Free-text Electronic Medical Records: A Phenotype Embedding Approach. *JMIR Med Inform* 2019;**7**:e14782. doi:10.2196/14782
24 Devlin J, Chang M-W, Lee K, *et al.* BERT: Pre-training of Deep Bidirectional Transformers for Language Understanding. In: *Proceedings of the 2019 Conference of the North American Chapter of the Association for Computational Linguistics: Human Language Technologies, Volume 1 (Long and Short Papers)*. Minneapolis, Minnesota: : Association for Computational Linguistics 2019. 4171–86. doi:10.18653/v1/N19-1423
25 Lee J, Yoon W, Kim S, *et al.* BioBERT: a pre-trained biomedical language representation model for biomedical text mining. *Bioinformatics* 2020;**36**:1234–40. doi:10.1093/bioinformatics/btz682
26 Mascio A, Kraljevic Z, Bean D, *et al.* Comparative Analysis of Text Classification Approaches in Electronic Health Records. *arXiv:200506624 [cs]* Published Online First: 8 May 2020.http://arxiv.org/abs/2005.06624 (accessed 18 Jan 2021).
27 Si Y, Wang J, Xu H, *et al.* Enhancing clinical concept extraction with contextual embeddings. *Journal of the American Medical Informatics Association : JAMIA* 2019;**26**. doi:10.1093/jamia/ocz096
28 Bhavsar V, Sanyal J, Patel R, *et al.* The association between neighbourhood characteristics and physical victimisation in men and women with mental disorders. *BJPsych open* 2020;**6**:e73. doi:10.1192/bjo.2020.52
29 Jackson RG, Patel R, Jayatilleke N, *et al.* Natural language processing to extract symptoms of severe mental illness from clinical text: the Clinical Record Interactive Search Comprehensive Data Extraction (CRIS-CODE) project. *BMJ Open* 2017;**7**:e012012. doi:10.1136/bmjopen-2016-012012
30 Viani N, Botelle R, Kerwin J, *et al.* A natural language processing approach for identifying temporal disease onset information from mental healthcare text. *Scientific Reports* 2021;**11**:757. doi:10.1038/s41598-020-80457-0
31 Perera G, Broadbent M, Callard F, *et al.* Cohort profile of the South London and Maudsley NHS Foundation Trust Biomedical Research Centre (SLaM BRC) Case Register: current status and recent enhancement of an Electronic Mental Health Record-derived data resource. *BMJ Open* 2016;**6**:e008721. doi:10.1136/bmjopen-2015-008721
32 Clinical Record Interactive Search (CRIS). https://www.slam.nhs.uk/quality-and-research/clinical-record-interactive-search-cris/ (accessed 2 Feb 2021).

For peer review only

33 World Health Organization, editor. *International statistical classification of diseases and related health problems*. 10th revision, 2nd edition. Geneva: : World Health Organization 2004.

34 Effectiveness of anonymised information sharing and use in health service, police, and local government partnership for preventing violence related injury: experimental study and time series analysis | The BMJ. https://www.bmj.com/content/342/bmj.d3313 (accessed 28 Jan 2021).

35 Clark C, Ryan L, Kawachi I, *et al.* Witnessing Community Violence in Residential Neighborhoods: A Mental Health Hazard for Urban Women. *J Urban Health* 2008;**85**:22–38. doi:10.1007/s11524-007-9229-8

Figure legends:

Figure 1: Process of annotation, development and evaluation of NLP models

Figure 2: Flowchart of extract annotation process

For peer review only

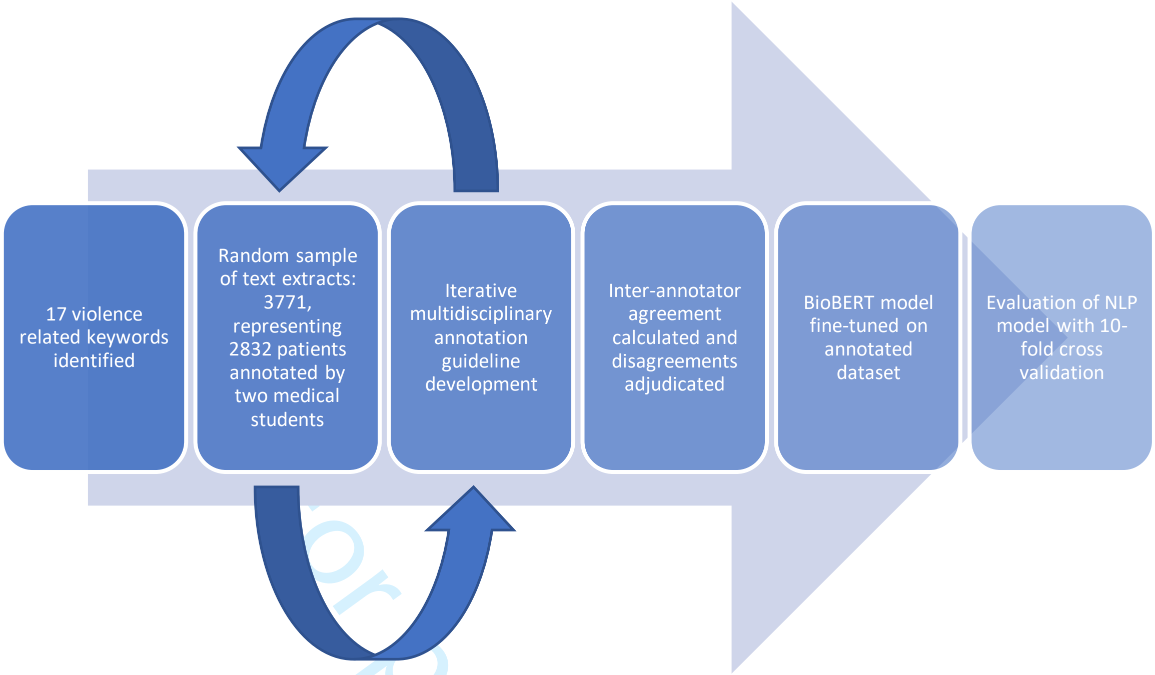

For peer review only

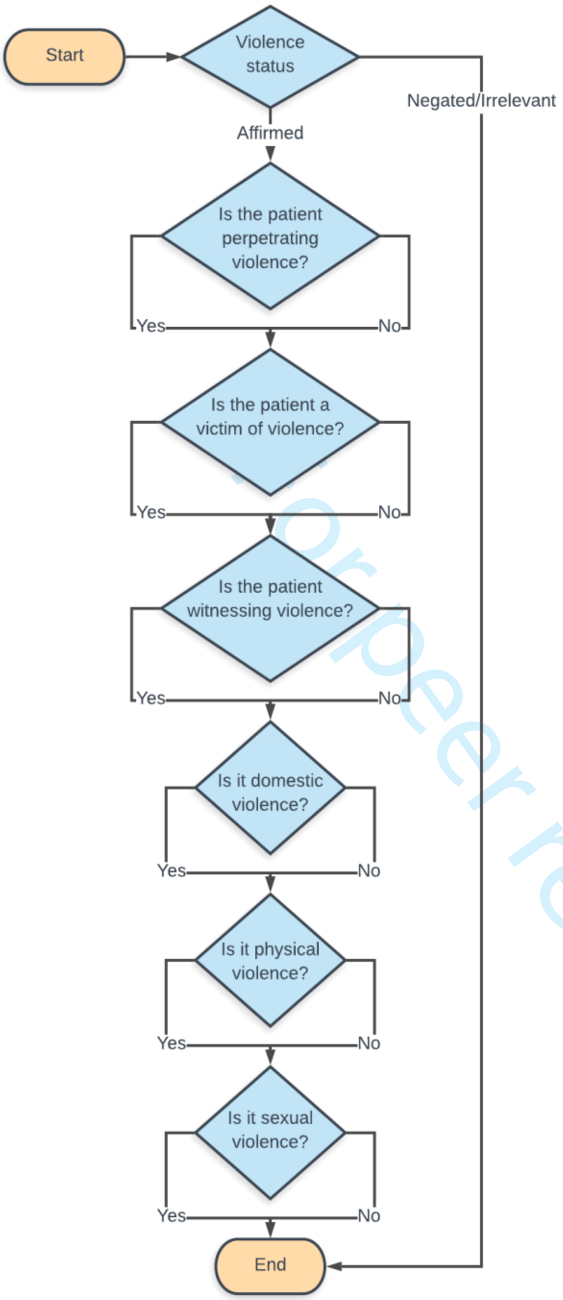

Figure 2: Flowchart of extract annotation process

Appendix 1

Keywords

Nouns

- % abus%
- % attack%
- % beat%
- % violenc%
- % hit%
- % rape%
- % assault%

Verbs:

- % fight%
- % fought%
- % slap%
- % chok%
- % push%
- % punch%
- % strangul%
- % strangl%
- % threw%
- % struck%

For peer review only

Appendix 2: Guidelines

Guidance for violence annotations

Development team: Aurelie Mascio, Sumithra Velupillai, Riley Botelle, Marcus Williams, Rob Stewart, Vishal Bhavsar, Giouliana Kadra

Annotations were made for:

a) *violence* status: **affirmed** or negated or irrelevant. When annotated as affirmed, further annotations were made for:

b) *patient* status: perpetrator and/or victim and/or witness and/or other, and

c) violence *type*: physical and/or sexual and/or domestic (or none of the above).

NOTE: there was not a sufficient amount of examples to develop a robust classification model for *witness*, thus there is no corresponding NLP application for this category.

For peer review only

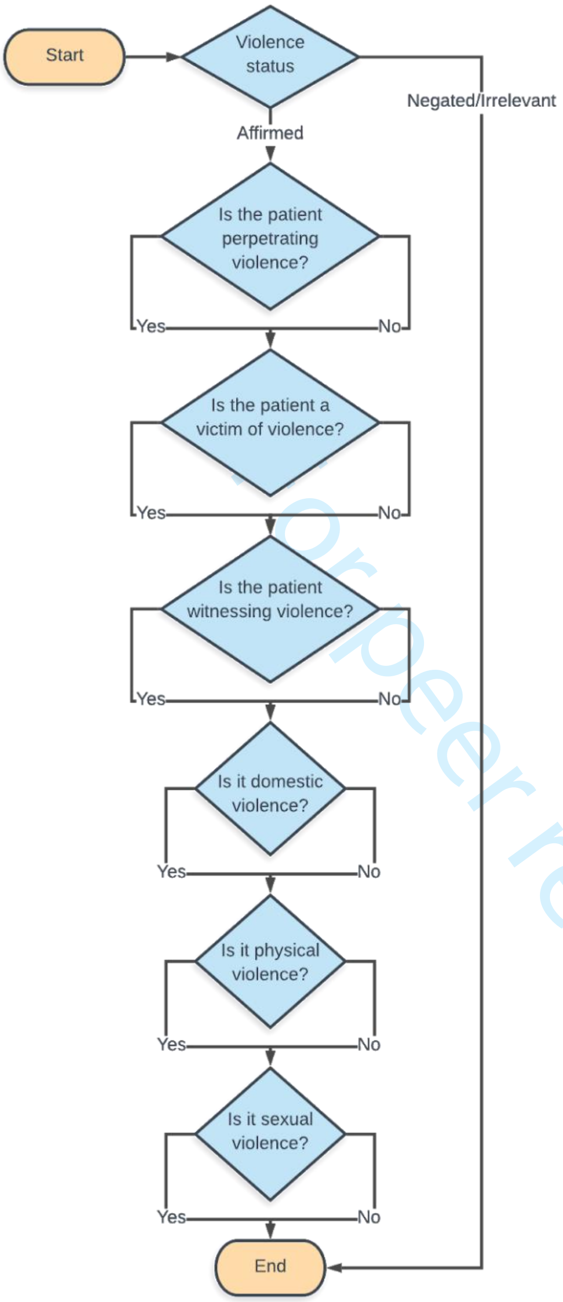

Violence status

Definition

Violence is the use of physical force or power, threatened or actual, against another person that results in - or has a high likelihood of resulting in - injury, death, psychological harm, maldevelopment, or deprivation. This does not just cover physical violence, but also violent threats, neglect, racial, digital, sexual abuse, financial abuse and coercion and control.

Our definitions of violence and sexual violence largely align with the WHO definition of violence (see also: WHO 2002 report), but with the criteria for intentionality removed due to an inability to determine this in most extracts. The violence definitions are specifically interpersonal, rather than including violence to self.

Annotation examples and criteria:

**Violence Status Decisions**		
**Examples**	**Classification**	**Explanation**
"They were *abused* in their childhood"	Affirmed	
"His brother would *beat* him up if he found out", "risk of *violence*"	Irrelevant	**Hypothetical scenarios** are irrelevant
"Expressed a lot of interest in *violence*, nazism"	Irrelevant	**Fantasies and ideological violence** is irrelevant
"Patient is *hitting* himself"	Irrelevant	**Self injury** is irrelevant
"Patient is *abusive* towards their pet"	Irrelevant	**Violence towards animals** is irrelevant
"Patient shows *violent* behaviour such as breaking objects and punching walls"	Irrelevant	**Violence directed towards objects**/property is irrelevant, affirmed only if clear intention to cause harm or fear to others.
"I have enclosed a leaflet for sexual abuse services" "referred to the domestic *violence* advisor"	Irrelevant	**Referrals** are irrelevant.
Learning difficulties/REM sleep behaviour disorder/violence without intent to harm	Affirmed	Violence is affirmed regardless of **intent**.
"Acquitted as accomplice to gang *rape* (negated) . . . convicted of ABH (affirmed)"	See explanation	When there are **multiple incidents** within the text use the keyword to determine if negated or affirmed.
"Denied the allegations he *hit* members of staff" "Questioned by police recently for allegedly *hit*ting his ex-girlfriend however he denied he was present." "Allegation made by patient of repeated sexual *assault* . . . on detailed questioning the patient denied being repeatedly assaulted"	See explanation	Denying incident = negated<br>Allegation = affirmed<br>Allegation and denial (**contrasting claims**) = affirmed<br>Not guilty = negated<br>Charges dropped = affirmed<br>Acquitted = negated<br>If confusing or unsure mark as irrelevant
"He told his mother he plans to hit them with a baseball bat"<br>"He made a gesture to attack staff"	Affirmed	**Plans** for violence are affirmed, similarly gestures or 'appearances' of violence are affirmed
"Patient was *abus*ing staff verbally" "patient made threats to kill and *attack* staff"	Affirmed	**Verbal/threats** of violence are affirmed as violence status

For peer review only

		(but are not affirmed as physical violence, see below?)
"No *violence* or aggression noted"	Negated	
"Found the way they were brought to the hospital very traumatic and therefore felt very *abus*ed by mental health services"	Irrelevant	**Institutional and structural violence** is irrelevant. Interpersonal violence by individuals within institutions (e.g. beaten by police, abused by prison guards) would still be affirmed
"she repeatedly called the police making allegations of being posioned on the ward . . . she also made allegations of physical and sexual *abuse* by patients and staff"<br>"the voices . . . threaten to harm her and sometimes she feels them *hit* her"	Affirmed or Irrelevant	For allegations where the patient is clearly recorded as **hallucinating or delusional** within the fragment, mark as irrelevant. If unclear if delusional or a real event, mark as affirmed.
"Forced to *fight*" (in a war/army), "was not tortured but witnessed dead bodies and forced to move frequently due to *fight*ing"	Irrelevant	**Military service** should not be captured - irrelevant<br>Collective violence should not be captured - irrelevant
"Suffered financial abuse"	Affirmed	Financial, psychological, emotional and **other types of abuse** including deprivation and neglect are affirmed.
"He has a history of *violence*"<br>"patient used to *hit* her partner"<br>"Is not as verbally *abus*ive as she used to be"	Affirmed	**Historical abuse** is affirmed.

Patient status

Definition

The **perpetrator** is the person using physical force or power, the **victim** is the person this is used against. A **witness** is someone who directly observes acts of violence through sight and/or sound.

Annotation examples and criteria:

**Patient Status Decisions**		
**Examples**	**Classification**	**Explanation**
"he stabbed someone who had *attack*ed him",	Affirmed, perpetrator	Patient status may be any combination of victim and perpetrator and witness.

"Arrested following violence and fire setting . . . having been raped 3 years ago"	and/or victim and/or witness	
"They often argued and fought", "got into a fight", "became involved in a fight", "they had fought"	See explanation	Generally for vague **"involvement" in fights** patient status should be marked as "other". If there is clear initiation of the fight they would be the perpetrator, if there is explicit harm or intended harm to a party they would be the victim or perpetrator accordingly.
"tried to rob him . . . he *fought* back and they fled", "talked about his brother bullying him as a child until he *fought* back", "he felt another youth was threatening to him and *fought* back violently"	Perpetrator and victim	Where there is **retaliatory violence or self defence** mark the patient as both perpetrator and victim
"his son got *beat* up on the way home from school"	Other	**Indirect witnesses** or where it is unclear if they saw/heard/witnessed themselves should be marked as other.
"Watched his father being killed"	Witness	Clearly **direct witnesses** should be marked as witness.

Violence type

Definition

Domestic violence is violence between family members, intimate partners, ex-intimate partners and household members.

Physical violence is violence that uses physical force, or results in or has a high likelihood of resulting in physical injury to the victim.

Sexual violence includes any unwanted sexual act, unwanted sexual comments or advances, unwanted attempts to obtain a sexual act, directed acts against a person's sexuality using coercion and acts to traffic. It includes (but is not limited to) rape, sexual harrassment, sexual assault, forced marriage, FGM, and reproductive coercion and control.

Domestic violence is based on the UK government domestic abuse statutory guidance framework 2020, but adjusted to be applicable to all ages (rather than being restricted to over 16s) and includes people who are household members through choice (but not those in institutional, residential, detention or in-patient settings). Thresholds for considering violence physical are partially adapted from UK law, for instance "offences against the person" (1861 Act) describes crimes which are committed by direct physical harm or force and includes spitting as a form of assault and poisoning as a non-fatal, non-sexual offence.

Annotation examples and criteria:

For peer review only

Violence Type Decisions		
**Examples**	**Classification**	**Explanation**
**Violence Type Decisions (Physical)**		
"physical discipline" which was not perceived though as physical *abuse.*" "hit or made to kneel by father . . . did not see this as *abus*ive"	See explanation	Varies on keyword - these would be negated based on the negation of "abusive" keyword, but affirmed if the keyword was "physical discipline" or "hit".
"false imprisonment"/"abduction"	Physical = yes	
"spitting"	Physical = yes	Legally considered physical assault
"spiked" (drink) or poisoning	Physical = yes	Legally considered assault by poisoning
"Tried to *hit*", "Had to be stopped from *assaulting*"	Physical = yes	**Attempts to inflict physical violence** should be marked as physical
"*Rape*", "sexual *assault*"	Physical = yes, sexual = yes	**Physical sexual violence** should be marked as both physical and sexual
**Violence Type Decisions (Domestic)**		
"Partner's son", "brother's girlfriend", "father"	Domestic = yes	**Familial relationships** (biological or adoptive) are considered domestic
"patient stabbed his roommate"	Domestic = yes	Those living together by choice (**households**) are domestic e.g. flatmates and cohabitees
"*abuse* she suffered whilst in care"	Domestic = no	**Residential and institutional settings** are not domestic including inpatient wards, prisons and care homes
"Hitting her ex-girlfriend"	Domestic = yes	**Partners and ex-partners** are considered domestic
**Violence Type Decisions (Sexual)**		

For peer review only

Sexual harassment	Sexual = yes, physical = no	**Sexual harrassment** is considered to be sexual violence.
“talked to female staff about his sexual fantasies”, “indecent exposure”	Sexual = yes	Mark as sexual violence if interpersonal (e.g. directed at or caused harm to another person)
“Patient was sexually disinhibited”, “Patient showed sexually inappropriate behaviour”	Irrelevant	Mark as irrelevant if not person-directed or no clear harm to another person.

For peer review only

Appendix 3: Guideline development discussion

Definitions of violence

An affirmed instance of violence - what should this mean?

Our NLP definition: Violence is the use of physical force or power, threatened or actual, against another person that results in - or has a high likelihood of resulting in - harm.

Harm in this instance does not refer only to physical injury, but also covers abuses of power such as violent threats and intimidation, acts which result in emotional and psychological harm, neglect, sexual abuse, financial abuse and coercion and control. It does not include violence against the self.

This definition was developed from the WHO definition of violence (Krug, 2002), but adjusted using deductive reasoning based on the annotated extracts and guideline development discussions, and therefore differs in two key ways. The first is that it only identifies interpersonal violence, and the second is that it does not require the violence to be intentional in order to be affirmed. These two changes are because there are various limitations to how violence can be affirmed in a way that can be applied reliably to EHR data.

It must be interpersonal because violence that is perpetrated systemically, state violence and structural violence is harder to identify, and tends to be poorly recorded. For example the quotation that a patient "found the way they were brought to the hospital very traumatic and therefore felt very *abus*ed by mental health services" is marked as irrelevant in this approach, as there is no identifiable interpersonal violence, however "*beat*en by prison guards" would be affirmed. Violence against whole communities is also poorly captured without an interpersonal element. We chose not to include self-directed violence.

This definition excludes acts of violence that are hypothetical ("risk of *violence*") and those that are fantasies or ideological ("expressed a lot of interest in *violence*, nazism"). It includes plans for violence ("he told his mother he plans to *hit* them with a baseball bat") and attempts made at violence which are thwarted ("had to be stopped from *assaulting*"). Whilst traditional definitions of violence place importance on the concept of intentionality, in EHRs generally the data recorded is of incidents and histories, not motivations. Intentions may be repressed, denied, disagreed upon or not recorded at all. Therefore whilst intentionality is considered part of violence and hence plans and attempts are affirmed, it was considered not necessary for intentionality to be clear in order for the instance of violence to be affirmed in the NLP approach.

Annotation was additionally complicated by the mental status of patients and resultantly the reliability of the annotators (or even the original record authors) to determine whether a patient is hallucinating or delusional when reporting a violent event. A violent event cannot be assumed to be false because of its perceived improbability, and even when some aspects are impossible other aspects may be part of a real experience. Guidelines on this must choose whether to err on the side of affirming violence that did not happen against the risk of misassigning true experiences of patient-reported violence as irrelevant. The problem with the latter is that it may create a situation where patients with delusional or hallucinatory experiences are less likely to have instances of violence identified, and we considered that the resultant harms from this outweighed the overestimation of violence by including some false events. For instance, the example "she repeatedly called the police making allegations

For peer review only - http://bmjopen.bmj.com/site/about/guidelines.xhtml

of being posioned on the ward . . . she also made allegations of physical and sexual *abuse* by patients and staff" was not be assumed to be a delusional experience, as this risked masking real violent events. Contrastingly however "the voices . . . threaten to harm her and sometimes she feels them *hit* her" is explicitly a hallucinatory experience, and therefore not interpersonal and not affirmed.

Other difficulties with identifying violence reflect the entanglement of multiple viewpoints within the text. How should we classify a violent act that the author is asserting happened but the patient denies? Or similarly, an allegation by one party and a denial by another in the same text, or even an allegation and later denial by the same patient? (For instance, "allegation made by patient of repeated sexual *assault* . . . on detailed questioning the patient denied being repeatedly assaulted.") How should legal terms be interpreted - someone who is charged, or acquitted, or who's case against them is dropped? Our approach tended towards affirming the identification of violent acts where there was a degree of uncertainty or disagreement as to whether they occurred or not, and only excluding those where everyone was in agreement there had been no violence, for the same concerns about harms resulting from the classification of reported violence as false. This overall approach should lend itself to an output where the application is more likely to produce false positives than false negatives.

Thresholds for considering something as violence could also be challenging. Our definition includes actions or inaction that results in harm, but what may cause harm for one person may not cause harm to others. As a general rule the guidelines do not include violent acts against property or objects as these are not interpersonal, but destroying a valued object to punish someone or throwing something in order to incite fear are both interpersonal violent acts that should be identified and affirmed. Violence towards animals was similarly excluded from the definition for not being interpersonal. There was occasionally difficulty in differentiating between aggressive behaviour and violence. The majority of aggressive behaviour, if directed at another person, fulfils the definition of violence and was appropriately accounted for by these guidelines.

Defining patient status - who did what?

Our NLP definition: The **perpetrator** is the person using physical force or power, the **victim** is the person this is used against. A **witness** is someone who directly observes acts of violence through sight and/or sound.

Identifying the role of the patient (presupposing you can identify who the patient is in the text) was on average a straightforward process, and usually explicitly stated. There are two exceptions we found to this; instances where there was some level of mutuality in the violence, and instances where the violence was retaliatory or undertaken as self-defence. To resolve these problems, patients could be assigned both as the perpetrator and the victim in scenarios where they both enacted and received violence. It has been argued that self-defence should not be considered violence (Hamby, 2017) but extracts from EHRs often included disagreement as to whether an event was truly self-defence or not, and thus following the rules for disagreements between people or the authors of the texts, were marked as affirmed.

Types of violence

For peer review only

Our NLP definition of physical violence is violence that uses physical force, or results in or has a high likelihood of resulting in, physical injury to the victim.

Where there was uncertainty about whether an act should be considered physical violence, such as spitting and poisoning, an exemplar approach was utilised. These utilised categorisations adapted from UK law such as “offences against the person” (1861 Act) which describes crimes which are committed by direct physical harm or force and includes spitting as a form of assault and poisoning as a form of non-fatal, non-sexual offence. False imprisonment and abduction were agreed by discussion to be a form of physical violence due to the physically restricting nature of the act.

One difficulty with establishing whether physical violence should be affirmed is with acts of physical violence that were not considered to result in harm by the victim, such as “hit or made to kneel by father . . . did not see this as *abus*ive”. There are two potential approaches here; either choosing which to prioritise between actions and harms, or using the specific keywords to make two separate judgements. With the latter approach, the example would appear twice, once as affirmed for “hit” and once as negated for “abusive”.

Our NLP definition of domestic violence is violence between family members, intimate partners, ex-intimate partners and household members.

Our definition of domestic violence is designed to capture a relational aspect between the victim and perpetrator; that is it represents a family, intimate partner and/or domestic (household) relationship.

Some legal definitions of domestic violence (e.g. South African Domestic Violence Act 1998) include housemates and flatmates who share or have recently shared the same residence, whereas others only refer to previous or current intimate partners and family members. Our definition, whilst including household members, only includes those who live together through choice. Therefore it includes housemates and cohabitees but not those in care homes, prisons, residential or inpatient psychiatric settings.

The UK government definition does not include those under the age of 16 within their cross-governmental definition of domestic violence, but we chose to include children and adolescents in our approach, in part because it is not always possible to determine the age of the patient from the extract but also because we felt it was not appropriate to exclude them.

The rationale for identifying domestic violence specifically is that the interventions are distinct from those designed for community violence (violence perpetrated by strangers or acquaintances) (Krug, 2002).

Our NLP definition of sexual violence includes any unwanted sexual act, unwanted sexual comments or advances, unwanted attempts to obtain a sexual act and acts to traffic. It includes rape, sexual harrassment, sexual assault, forced marriage, stalking and reproductive coercion and control.

Forms of sexual violence that fulfil the definition for physical violence, such as rape and sexual assault, were recorded as both physical and sexual violence.

A challenge with defining sexual violence in psychiatric EHRs are the symptoms of sexual disinhibition and sexually inappropriate behaviour. Whilst the approach does not consider intentionality necessary for the affirmation of a violent act, these acts and behaviours were

For peer review only

not considered sexual violence unless they were directed towards another person (as opposed to generalised) or they explicitly caused harm to another person

For peer review only
